# Host Genetic Factors and Vaccine-Induced Immunity to HBV Infection: Haplotype Analysis

**DOI:** 10.1371/journal.pone.0012273

**Published:** 2010-08-18

**Authors:** Kelli K. Ryckman, Katherine Fielding, Adrian V. Hill, Maimuna Mendy, Pura Rayco-Solon, Giorgio Sirugo, Marianne A. van der Sande, Pauline Waight, Hilton C. Whittle, Andrew J. Hall, Scott M. Williams, Branwen J. Hennig

**Affiliations:** 1 Center for Human Genetic Research, Vanderbilt University, Nashville, Tennessee, United States of America; 2 Department of Pediatrics, University of Iowa, Iowa City, Iowa, United States of America; 3 London School of Hygiene and Tropical Medicine, London, United Kingdom; 4 Welcome Trust Centre for Human Genetics and The Jenner Institute, Oxford University, Oxford, United Kingdom; 5 Medical Research Council Laboratories, Banjul, The Gambia; 6 Medical Research Council International Nutrition Group, London School of Hygiene and Tropical Medicine, UK and Medical Research Council, Keneba, The Gambia; 7 Unita' di Genetica Medica, Ospedale S. Pietro FBF, Rome, Italy; 8 National Institute for Public Health and the Environment (RIVM), Bilthoven, The Netherlands; Albert Einstein Institute for Research and Education, Brazil

## Abstract

Hepatitis B virus (HBV) infection remains a significant health burden world-wide, although vaccines help decrease this problem. We previously identified associations of single nucleotide polymorphisms in several candidate genes with vaccine-induced peak antibody level (anti-HBs), which is predictive of long-term vaccine efficacy and protection against infection and persistent carriage; here we report on a haplotype-based analysis. A total of 688 SNPs from 117 genes were examined for a two, three and four sliding window haplotype analysis in a Gambian cohort. Analysis was performed on 197 unrelated individuals, 454 individuals from 174 families, and the combined sample (N = 651). Global and individual haplotype association tests were carried out (adjusted for covariates), employing peak anti-HBs level as outcome. Five genes (*CD44, CD58, CDC42, IL19* and *IL1R1*) had at least one significant haplotype in the unrelated or family analysis as well as the combined analysis. Previous single locus results were confirmed for *CD44* (combined global p = 9.1×10^−5^ for rs353644-rs353630-rs7937602) and *CD58* (combined global p = 0.008 for rs1414275-rs11588376-rs1016140). Haplotypes in *CDC42, IL19* and *IL1R1* also associated with peak anti-HBs level. We have identified strong haplotype effects on HBV vaccine-induced antibody level in five genes, three of which, *CDC42*, *IL19* and *IL1R1*, did not show evidence of association in a single SNP analyses and corroborated the majority of these effects in two datasets. The haplotype analysis identified associations with HBV vaccine-induced immunity in several new genes.

## Introduction

Hepatitis B virus (HBV) infection remains a significant health burden world-wide, especially in parts of Asia and Sub-Saharan Africa[1]. Approximately 2 billion people have been infected with HBV and of these 350 million live with persistent infection. Some 600,000 deaths annually result from HBV infection. Infant immunisation against infection has been available since the early 1980s and the vaccine is now routinely administered across many regions where the disease is endemic. Vaccination in infancy is 95% effective and ameliorates most of the consequences of HBV exposure. For example, long-term studies in The Gambia have shown that infant immunisation is safe and that a good vaccine-induced immunity, i.e. high peak antibody (anti-HBs) level, correlates with vaccine efficacy long-term [2]. Thus, vaccination is thought to reduce the risk of developing later complications, including hepatocellular carcinoma. However, the efficacy of the vaccine is not universal, vaccine failure has been observed in approximately 5% of vaccinees[3], and infection, as shown by seroconversion, can occur despite vaccination (“breakthrough” infection) [2]. Vaccine-induced immunity is influenced by a number of factors including type of vaccine, administration route, age, gender, UV light exposure, smoking, co-infections, and nutritional factors [4]. It is also thought that the immune response to vaccination (as well as susceptibility to disease and disease progression) is partially affected by host genetics, but our understanding of the role of these factors is very limited. Family and twin studies indicate that human genetic variation modulates HBV vaccine-induced immunity, with heritability estimates in Gambian twins ranging between 63–85% [5]–[11]. Case-control association studies have also emphasized the influence of genetic variation on vaccine-induced immunity, but these studies have primarily concentrated on the HLA region, but most of these reports are based on a small number candidate SNPs/genes [12]–[15]. There are two exceptions: A recent study in Indonesians based on the analysis of over 5000 SNPs across 914 genes in HBV vaccine responders versus non-responders [16]; our own study assessed 715 SNPs across 133 genes and their effect on peak HBV vaccine-induced antibody level in infant vaccinees from The Gambia [17] However, in both of these cases only single SNP association analyses were performed.

In a previous report we tested for association with vaccine-induced peak anti-HBs, which is predictive of long-term vaccine efficacy, as well as protection against infection and persistent carriage, and identified single SNP associations in *CD58* (rs1414275, rs1016140), *IFNG* (rs2069727), *MAPK8* (rs3827680, rs10857565), *IL10RA* (rs2508450, rs2229113) and CD44 (rs353644, rs7937602), and multi-marker associations in *IFNG*, *MAPK8*, *IL10RA* at the Geometric Mean Titer (GMT) level of >1.5 or <0.6 at p≤0.001 level (for details see table 3 and supplementary table S2 in [17]). Here we extend our previous investigation, performing a more in depth analysis of our data from the same Gambian population of infant vaccinees, to include an analysis of haplotypes and their possible associations with vaccine response. This analysis was performed under the assumption that in some cases haplotypes can detect associations with genes that single SNP analyses cannot [18]. This assumption is based on the fact that haplotypes define functional units of genes and variation is structured into haplotypes that are likely to be transmitted as units; in addition employing haplotype analysis reduces the problem of testing individual associations, potentially making haplotype analysis more powerful to detect associations than individual SNPs. Additionally, it has been shown that haplotype analyses can detect associations if there are multiple risk alleles at a single locus when single marker analyses may not [19]. Therefore, we generated haplotypes and tested for association with our outcome measure (peak anti-HBs level) and compared these findings to previous single marker results.

## Methods

### Study population

Demographic and hepatitis B specific serology data was available from a survey of vaccine efficacy carried out in 2003 in The Gambia, which evaluated the magnitude and duration of protective antibody responses induced by infant HBV vaccination [2]]. The effect of host genetic variation on HBV vaccine-induced immunity were assessed in this same population based on a larger first screen of 715 SNPs in 662 infant vaccines and a much smaller second screen (43 SNPs in 393 individuals) [17]. Of the 662 individuals that were part of the original single locus analysis [17] we excluded 11 due to insufficient genotype data and used the data on the remaining 651 infant vaccines for the present haplotype analysis.

Briefly, study participants were recruited in the West Kiang region in The Gambia who had been included in the HBV vaccine programme, which has been running since 1984 [2]. Only non-infected individuals were included, i.e. confirmed non-immune children <5 years old prior to 1985 and thereafter all those vaccinated at birth (i.e. those deemed anti-HBc negative). Concentrations of hepatitis B surface antibody at ∼11 months of age (peak anti-HBs) and anti-HBc status at follow-up surveys were determined by radioimmunoassay (Sorin Biochemica) or EIA (ETIAB-Corek Plus and ETI-AB-AUK; DiaSorin) in accordance with the manufacturer's instructions [2]. For the genetic study a subset of vaccinees was genotyped, this first screen included all core antibody (anti-HBc) positives and two randomly selected age-group matched anti-HBc negative subjects to allow analysis of “breakthrough infection” as outcome as well as peak vaccine-induced antibody level. However, due to small numbers of anti-HBc positives, the present analysis was based on peak anti-HBs level only.

The study participants were mostly residents of three villages (plus 33 ‘others’ grouped with the village of Kantonkunda), the great majority (625 or 96.01%) were of Mandinka origin, the mean age was 13.4 (+/−5.4), 47.9% were male, six different vaccine regimes had been administered across the study population and all had received 3 or 4 doses, with exception of three individuals (who had received 2 doses). Relatedness within families was established according to maternal ID. Individuals for whom no information on peak anti-HBs level or date, peak anti-HBs measurement time less than one month after the last vaccination, no information on number of doses of vaccine, age >5yrs at time of last vaccination and those belonging to vaccine group 6 (n = 2) were excluded (for further details see [17] Table 1).

**Table 1:** Haplotype effects for in unrelated, family and combined data sets for *CD44* (rs353644-rs353630-rs7937602) a.

		Unrelated (Unadjusted p = 0.07, Adjusted p = 0.002*)				Family (Unadjusted p = 0.004*, Adjusted p = 1.0)				Combined (Unadjusted p = 0.001*, Adjusted p = 9.1×10^−5^*)		
Haplotype	Haplotype	Freq	Add Val (95% CI)	P-value	P-value adjusted	Freq	Add Val (95% CI)	P-value	P-value adjusted	Add Val (95% CI)	P-value	P-value adjusted
**A-A-A**	**1-1-1**	0.37	0.12 (−0.38–0.63)	0.72	0.99	**0.33**	−1.06 (−2.08–0.04)	**0.55**	1.5×10^−3^*	−0.11 (−0.30–0.069)	0.97	0.93
A-A-C	1-1-2	0.16	0.64 (−0.15–1.42)	0.02*	0.82	0.14	2.21 (−0.73–5.15)	0.76	0.02	0.57 (0.041–1.10)	0.02*	0.28
A-G-A	1-2-1	0.27	referent	0.08	0.76	0.33	referent	0.07	0.14	referent	0.71	0.27
G-G-A	2-2-1	0.17	0.41 (−0.27–1.10)	0.91	0.90	0.15	1.03 (−1.43–3.50)	6.4×10^−5^*	0.43	−0.24 (−5.99–5.52)	0.01*	1.0

a Equivalent to SNP IDs 440, 441 and 442 in [17].

Adjusted p-values are corrected for measurement time (between last vaccination and peak antibody level assessment) and vaccine group (six regimes since 1984).

Asterisks denote results significant after correction for multiple testing. Additive values and 95% Confidence Intervals are given for haplotypes after adjusting for measurement time and vaccine group. Correction for multiple testing using false discovery rate (FDR – q = 0.2) was performed separately in the unrelated, family and combined data accounting for 1,376 tests in the unadjusted and 111 tests in the adjusted analysis, respectively ^19^. For specific haplotype tests correction for multiple testing with FDR accounts for the number of haplotypes examined (4 haplotypes). Therefore anything <0.05 in the adjusted or unadjusted analysis was significant after correction for multiple testing.

DNA was extracted from whole blood or PBMCs using a standard salting-out method [20]. Ethical approval was granted by the joint Gambia Government/MRC Ethics Committee, and the LSHTM and Oxford University (OXTREC) Ethics Committees. All subjects and/or legal guardians provided written, informed consent.

### Candidate genes/SNPs and genotyping

Details on the selection of candidate genes/SNPs and genotyping methodology have been described in detail elsewhere [17]. Briefly, candidate genes/SNPs thought to play a role in immunity induced by immunisation against HBV or other vaccines were screened. The gene selection was based on literature searches, previous reported genetic associations, inclusion of gene families and coverage of regulatory pathways. The SNP selection was based on HapMap frequency data, validation, anticipated success rate on the Illumina platform, gene size, distribution across loci including 500 bp up- and down-stream of each gene. SNP genotyping was performed on the Illumina BeadArray platform (www.illumina.com). Assays with a failure rate of >10% and those with a minor allele frequency (MAF) of <1% were excluded; the average call success rate of the remaining markers was 99.69%; only data from the first genetic screen of our earlier study was used for the present analysis. The original study consisted of 715 SNPs in 133 genes. For the haplotype analysis genes were excluded if there was only one SNP in a gene (10 genes, 10 SNPs) and/or if a gene was located on the X chromosome (4 genes, 17 SNPs). X chromosome markers were excluded because these markers are best analyzed using gender stratification and our sample size is too small for this analysis. Hence, a total of 688 SNPs from 119 genes were ultimately included.

Bioinformatics tools were used for SNP function prediction (http://snpinfo.niehs.nih.gov/snpfunc.htm) and (http://fastsnp.ibms.sinica.edu.tw/pages/input_CandidateGeneSearch.jsp) as well as literature searches on associated candidate genes (www.pubgene.org).

To see if there is a common thread across the five genes associated with anti-HBs in this haplotype-based analysis, we used a simple bioinformatics tool, PubGene (www.pubgene.org) that searches PubMed for literature based on a single reference gene and identifies other genes that are found in conjunction with it in the literature.

### Haplotype and statistical analysis

**Figure 1:**
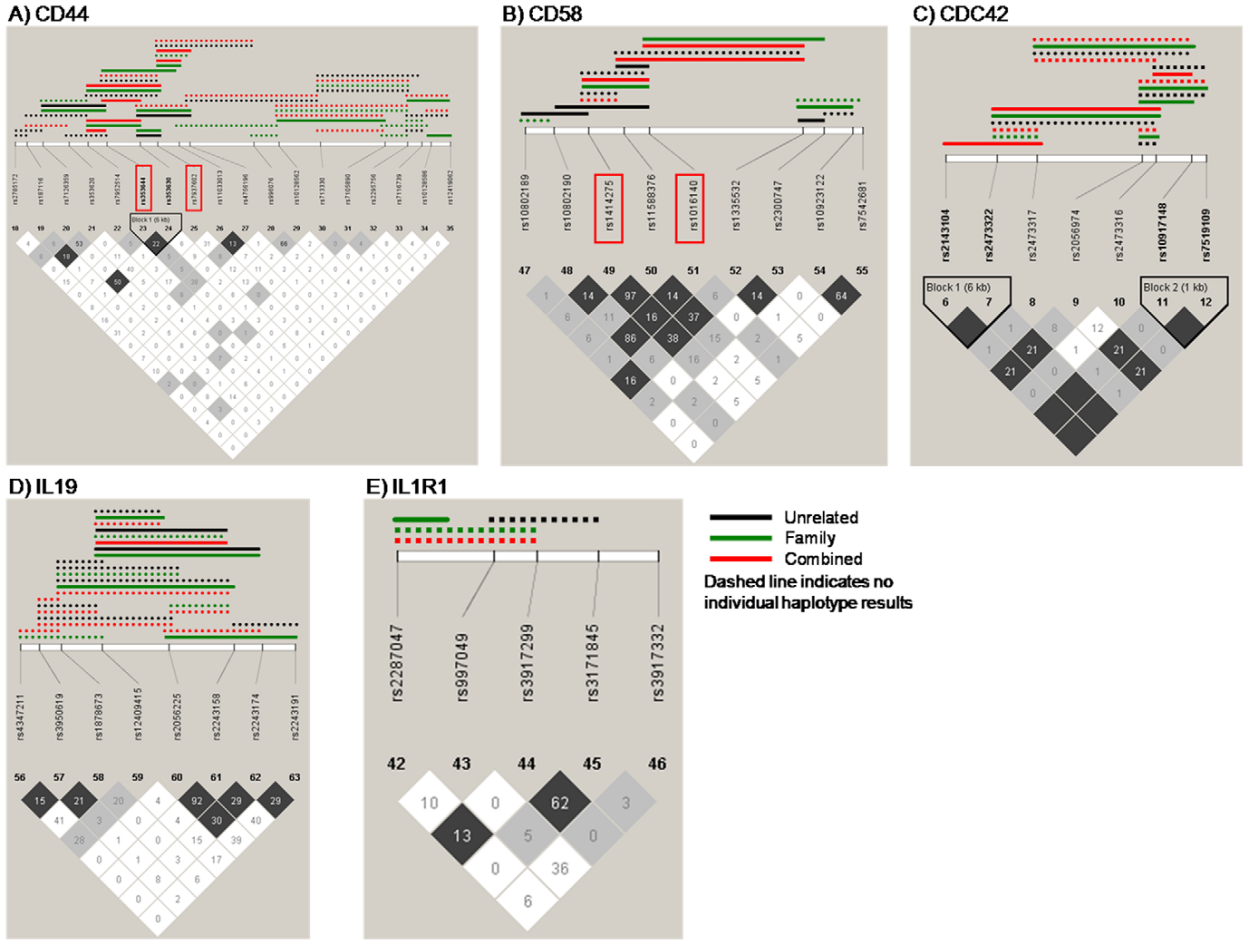
Graphical representation of LD and Haplotype associations with peak anti-HBs level (*adjusted analysis* ^§^). Solid lines indicate significant (p<0.05) global and individual haplotype associations with anti-HBs levels. Dotted lines indicate a significant global association but no individual haplotypic effects. Color of line denotes which study the significant association occurred in – black for the unrelated data, green for the family data and red for the combined data (unrelated and family together). The measure of LD employed was r^2^. Associated genes: A) *CD44*, B) *CD58* C) *CDC42*, D) *IL19* and E) *IL1R1*. ^§^ Analysis adjusted for measurement time (between last vaccination and peak antibody level assessment) and vaccine group (six regimes since 1984).

The study population comprised of both unrelated individuals and individuals from families. The analysis was performed separately on unrelated (197 independent individuals) and family (454 individuals from 174 families) data. Additionally the combined (unrelated and family datasets together – 651 individuals total) data was analyzed. For the combined analysis unrelated subjects were assumed to have two missing parents and parameters are set to give the likelihood contribution for the family. Two, three and four haplotype sliding windows were examined in unrelated, family and combined datasets for association with peak HBV vaccine-induced antibody level, i.e. the natural log of peak anti-HBs, using Unphased version 3.1.1 [21]. This software uses a quasi-Newton algorithm to maximize the likelihood of each haplotype when an individual's phase is unknown. The global test of association is a likelihood ratio test based on the null hypothesis that all of the haplotypes have equal risk. The p-value is determined with the rare haplotypes (<0.05) excluded. Additive values are calculated based on the change in expected trait value due to a given haplotype relative to the referent haplotype. The referent haplotype was chosen as the most frequent haplotype; however, in the case of CD44 (rs353644-rs353630-rs7937602), CD58 (rs1414275-rs11588376-rs1016140) and CDC42 (rs2056974-rs2473316) where the most frequent haplotype was also the most significantly associated with peak anti-HBs level, the referent was chosen as the haplotype least significantly associated in the unrelated, family or combined analyses. The p-value for each individual haplotype is calculated based on a score statistic for the effect of a given haplotype relative to all other haplotypes, including rare haplotypes, pooled together. The global test and the test for each individual haplotype may give different results due to reduced power when all of the individual haplotypes are parsed out. Therefore, more importance should be given to the global test value as this represents the association for the distribution of the haplotypes. No covariates were included in the initial analysis. Genes that had one or more significant haplotypes at p<0.1 in all three datasets (unrelated, family and combined) and at least one haplotype significant at p<0.01 in the combined data were carried forward for further analysis. There were 1,376 tests performed for each haplotype analysis. Correction for multiple testing was performed separately in the unrelated, family and combined data using false discovery rate (FDR – q = 0.2) [22]. Five genes met our selection criteria and were included in follow-up analyses (Table S2), with adjustment for the natural log of measurement time (between last vaccination and peak antibody level assessment) and vaccine group (six regimes since 1984). These covariates were selected because both are known to affect peak anti-HBs level [2], [17]. Number of vaccine doses, gender and village did not or only minimally affect vaccine-induced antibody level and were thus not included as covariates in this analysis, nor was ethnicity (96.01% Mandinka). Correction for multiple testing (111 tests) after adjustment for covariates was performed separately in the unrelated, family and combined data using false discovery rate (FDR – q = 0.2) [22]. Graphical representation of significant results and haplotypes were performed with Haploview (Figure 1 and Figure S1, adjusted and unadjusted, respectively) [23]. Linkage disequilibrium (LD) was calculated in Haploview using the unrelated dataset only, according to default criteria [24].

## Results

### Summary of global haplotype association tests

Peak anti-HBs level was employed as the outcome measure throughout. Of the 117 genes analyzed for haplotype association, there were 92 genes with at least one haplotype that had a p-value <0.1, 80 genes with at least one p-value <0.05 and 57 genes with at least one p-value <0.01 (all unadjusted). Ten genes had at least one haplotype that was significant (<0.1) in all three analyses (unrelated, family and combined). Only five of these ten genes had an association of at least one haplotype with p<0.01 in the combined dataset, these genes were followed-up with adjustment for measurement time and vaccine group. The majority of global association tests were more significant than individual haplotype results for our five top genes. In some cases we see non-significant individual haplotype results, while the global haplotypes are still very significant, which may be due to an issue of power when all of the individual haplotypes are parsed out. We consider the global test results the more important of the values because it represents the association for the distribution of haplotypes in one gene. Findings for *CD44*, *CD58*, *CDC42*, *IL19* and *IL1R1* are described individually below and summarised in Tables 1, 2, 3, 4 and 5. All specific haplotype effects discussed below were significant after correction for multiple testing with FDR (q = 0.2). Previous single locus associations (adjusted for measurement time and vaccine group only) are described in more detail in our previous paper [17] and summarised in Table S1 for ease of comparison.

**Table 2:** Haplotype effects in unrelated, family and combined data sets for *CD58* (rs1414275-rs11588376-rs1016140) ^a^.

		Unrelated (Unadjusted p = 0.01, Adjusted p = 0.02*)				Family (Unadjusted p = 0.001*, Adjusted p = 0.04*)				Combined (Unadjusted p = 7.0×10^−6^*, Adjusted p = 0.008*)		
Haplotype	Haplotype	Freq	Add Val (95% CI)	P-value	P-value adjusted	Freq	Add Val (95% CI)	P-value	P-value adjusted	Add Val (95% CI)	P-value	P-value adjusted
**A-A-A**	**1-1-1**	0.57	0.59 (−0.10–1.27)	0.01*	0.23	0.56	−0.09 (−1.05–0.88)	6.8×10^−4^*	1.0	0.40 (0.09–0.71)	1.6×10^−5^*	2.0×10^−4^*
**G-G-A**	**2-2-1**	0.32	0.10 (−0.44–0.64)	0.28	0.60	0.32	−0.54 (−2.08–1.01)	0.06	1.0	−0.009 (−0.32–0.30)	0.02*	0.002*
G-G-C	2-2-2	0.10	referent	0.01*	0.36	0.12	referent	0.01*	0.65	referent	2.4×10^−4^*	0.09

a Equivalent to SNP IDs 062, 063, and 064 in [17].

Adjusted p-values are corrected for measurement time (between last vaccination and peak antibody level assessment) and vaccine group (six regimes since 1984).

Asterisks denote results significant after correction for multiple testing. Additive values and 95% Confidence Intervals are given for haplotypes after adjusting for measurement time and vaccine group. Correction for multiple testing using false discovery rate (FDR – q = 0.2) was performed separately in the unrelated, family and combined data accounting for 1,376 tests in the unadjusted and 111 tests in the adjusted analysis, respectively ^19^. For specific haplotype tests correction for multiple testing with FDR accounts for the number of haplotypes examined (3 haplotypes). Therefore anything <0.05 in the adjusted or unadjusted analysis was significant after correction for multiple testing.

**Table 3:** Haplotype effects in unrelated, family and combined data sets for *CDC42* (rs2056974-rs2473316) ^a^.

		Unrelated (Unadjusted p = 0.03, Adjusted p = 0.007*)				Family (Unadjusted p = 0.07, Adjusted p = 0.004*)				Combined (Unadjusted p = 0.003*, Adjusted p = 0.006*)		
Haplotype	Haplotype	Freq	Add Val (95% CI)	P-value	P-value adjusted	Freq	Add Val (95% CI)	P-value	P-value adjusted	Add Val (95% CI)	P-value	P-value adjusted
**A-A**	**1-1**	0.77	8.58 (−0.98–18.13)	0.09	0.08	**0.67**	**−1.05 (−2.98**–**0.88)**	**0.10**	5.9×10^−22^*	**0.18 (−0.26**–**0.62)**	0.02*	0.02*
C-A	2-1	0.15	8.09 (−1.43–17.61)	0.008*	0.08	0.19	−2.44 (−5.18–0.30)	0.02*	1.0	−0.21 (−0.79–0.37)	6.3×10^−4^*	0.17
C-G	2-2	0.06	referent	0.43	0.87	0.09	referent	0.78	0.19	referent	0.49	0.89

a Equivalent to SNP IDs 023 and 024 in [17].

Adjusted p-values are corrected for measurement time (between last vaccination and peak antibody level assessment) and vaccine group (six regimes since 1984).

Asterisks denote results significant after correction for multiple testing. Additive values and 95% Confidence Intervals are given for haplotypes after adjusting for measurement time and vaccine group. Correction for multiple testing using false discovery rate (FDR – q = 0.2) was performed separately in the unrelated, family and combined data accounting for 1,376 tests in the unadjusted and 111 tests in the adjusted analysis, respectively ^19^. For specific haplotype tests correction for multiple testing with FDR accounts for the number of haplotypes examined (3 haplotypes). Therefore anything <0.05 in the adjusted or unadjusted analysis was significant after correction for multiple testing.

**Table 4:** Haplotype effects in unrelated, family and combined data sets for *IL19* (rs12409415-rs2056225-rs2243158) ^a^.

		Unrelated (Unadjusted p = 0.15, Adjusted p = 0.008*)				Family (Unadjusted p = 0.02, Adjusted p = 3.2×10^−5^*)				Combined (Unadjusted p = 8.7×10^−4^*, Adjusted p = 0.004*)		
Haplotype	Haplotype	Freq	Add Val (95% CI)	P-value	P-value adjusted	Freq	Add Val (95% CI)	P-value	P-value adjusted	Add Val (95% CI)	P-value	P-value adjusted
A-A-C	1-1-1	0.67	referent	0.15	1.0	0.66	referent	0.06	1.0	referent	0.03*	0.09
**A-T-G**	**1-2-2**	0.13	−0.77 (−1.46–0.08)	0.43	0.54	0.13	8.19 (3.35–13.03)	0.95	1.0	**0.10 (−0.019**–**0.21)**	**0.63**	0.03*
G-A-C	2-1-1	0.12	−1.03 (−1.89–0.16)	0.56	1.0	0.11	1.04 (−1.17–3.26)	0.27	1.0	0.035 (−0.11–0.18)	0.83	0.71
**G-T-G**	**2-2-2**	**0.07**	**0.18 (−0.62**–**0.97)**	0.05*	2.2×10^−4^*	0.09	−101.6 (−193.8–9.49)	0.03*	1.0	0.003 (−0.23–0.24)	0.006*	0.65

a Equivalent to SNP IDs 118, 119, 120 in [17].

Adjusted p-values are corrected for measurement time (between last vaccination and peak antibody level assessment) and vaccine group (six regimes since 1984).

Asterisks denote results significant after correction for multiple testing. Additive values and 95% Confidence Intervals are given for haplotypes after adjusting for measurement time and vaccine group. Correction for multiple testing using false discovery rate (FDR – q = 0.2) was performed separately in the unrelated, family and combined data accounting for 1,376 tests in the unadjusted and 111 tests in the adjusted analysis, respectively ^19^. For specific haplotype tests correction for multiple testing with FDR accounts for the number of haplotypes examined (4 haplotypes). Therefore anything <0.05 in the adjusted or unadjusted analysis was significant after correction for multiple testing.

**Table 5:** Haplotype effects in unrelated, family and combined data sets for *IL1R1* (rs2287047-rs997049-rs3917299) ^a^.

		Unrelated (Unadjusted p = 0.001*, Adjusted p = 0.85)				Family (Unadjusted p = 0.05, Adjusted p = 0.02*)				Combined (Unadjusted p = 0.005*, Adjusted p = 0.02*)		
Haplotype	Haplotype	Freq	Add Val (95% CI)	P-value	P-value adjusted	Freq	Add Val (95% CI)	P-value	P-value adjusted	Add Val (95% CI)	P-value	P-value adjusted
A-A-A	1-1-1	0.41	referent	0.51	0.71	0.42	referent	0.04*	0.32	referent	0.25	0.56
A-A-G	1-1-2	0.18	0.03 (−0.51–0.58)	0.08	0.70	0.14	0.079 (−0.58–0.74)	0.16	0.98	0.08 (−0.14–0.30)	0.02*	0.64
G-A-A	2-1-1	0.29	0.21 (−0.15–0.57)	0.18	0.37	0.28	−0.18 (−0.82–0.45)	0.02*	0.30	−0.11 (−0.32–0.10)	0.48	0.36
**G-T-A**	**2-2-1**	**0.09**	**−0.45 (−1.13**–**0.23)**	**0.53**	0.03*	0.14	0.09 (−0.53–0.71)	0.52	0.75	0.06 (−0.14–0.27)	0.37	0.75

a Equivalent to SNP 145, 146, 147 IDs in [17].

Adjusted p-values are corrected for measurement time (between last vaccination and peak antibody level assessment) and vaccine group (six regimes since 1984).

Asterisks denote results significant after correction for multiple testing. Additive values and 95% Confidence Intervals are given for haplotypes after adjusting for measurement time and vaccine group. Correction for multiple testing using false discovery rate (FDR – q = 0.2) was performed separately in the unrelated, family and combined data accounting for 1,376 tests in the unadjusted and 111 tests in the adjusted analysis, respectively ^19^. For specific haplotype tests correction for multiple testing with FDR accounts for the number of haplotypes examined (4 haplotypes). Therefore anything <0.05 in the adjusted or unadjusted analysis was significant after correction for multiple testing.

***CD44*** **.** In *CD44* fifty haplotype associations with peak anti-HBs level were identified in one or more unadjusted analysis (Table S3, Figure S1). In the adjusted global test the most significant haplotype in the unrelated and combined data sets consisted of three SNPs (rs353644-rs353630-rs7937602; p = 0.002 and p = 9.1×10^−5^, respectively; Figure 1a; Table 1). The strongest individual haplotype was A-A-A (p = 1.5×10^−3^) in the family data and this haplotype was associated with decreased peak anti-HBs level relative to the referent A-G-A haplotype. This haplotype included two SNPs previously found to correlate with peak anti-HBs level in the single locus analysis; heterozygotes for rs353644 had increased antibody level and carriers of the variant allele for rs7937602 decreased levels (Table S1).

***CD58*** **.** Fifty two haplotype associations with peak anti-HBs level were identified for *CD58* in one or more unadjusted analyses (Table S3, Figure S1). In the adjusted global analysis the most significant haplotype in all three data sets (unrelated, family and combined) consisted of three SNPs (rs1414275-rs11588376-rs1016140) (p = 0.02, p = 0.04, and p = 0.008, respectively) (Figure 1b, Table 2). The most significant individual haplotypes were A-A-A (p = 2.0×10^−4^) and G-G-A (p = 0.002) in the combined data and these haplotypes associated with an increased (A-A-A haplotype) and decreased (G-G-A haplotype) anti-HBs level compared to the referent G-G-C haplotype. This haplotype included both rs1414275 and rs1016140, which were associated with decreased peak anti-HBs in the single locus analysis (Table S1; note: rs11588376 and rs1414275 are in almost complete LD r^2^ = 0.99).

***CDC42***. In *CDC42* seventeen haplotype associations with peak anti-HBs level were identified in one or more unadjusted analyses (Table S3, Figure S1). In the adjusted global analysis the most significant haplotype in all three data sets consisted of two SNPs (rs2056974-rs2473316) (unrelated p = 0.007, family p = 0.004, and combined p = 0.006) (Figure 1c, Table 3). The A-A haplotype was the most significantly associated haplotype in the family (p = 5.9×10^−22^) and the combined data (p = 0.02). This haplotype was associated with decreased peak anti-HBs in the family data and increased peak anti-HBs in the combined dataset compared to the referent C-G haplotype. No single locus associations with peak anti-HBs had been identified previously (Table S1).

***IL19*** **.** Twenty four haplotype associations with peak anti-HBs level were identified for *IL19* in one or more unadjusted analyses (Table S3, Figure S1). In the adjusted global analysis the most significant haplotype in all three data sets consisted of three SNPs (rs12409415-rs2056225-rs2243158) (unrelated p = 0.008, family p = 3.2×10^−5^, and combined p = 0.004) (Figure 1d, Table 4). The most significant individual haplotypes were G-T-G (unrelated p = 2.2×10^−4^) and A-T-G (combined p = 0.03), both associated with increased peak anti-HBs level compared to the referent A-A-C haplotype. This haplotype included rs12409415, which showed an indication for association with increased peak anti-HBs in the single locus analysis (Table S1).

***IL1R1*** **.** In *IL1R1* nine haplotype associations with peak anti-HBs level were identified in one or more unadjusted analyses (Table S3, Figure S1). In the adjusted global analysis the most significant haplotype in the family and combined data sets consisted of three SNPs (rs2287047-rs997049-rs3917299) (unrelated p = 0.001, family p = 0.02, and combined p = 0.02) (Figure 1e, Table 5). The most significant individual haplotype was G-T-A (unrelated p = 0.03), which was associated with decreased anti-HBs level compared to the referent A-A-A haplotype. In the single locus analysis there was an indication for association of a different SNP, rs3917332, with increased peak anti-HBs identified previously (p = 0.12, Table S1).

## Discussion

Peak anti-HBs level induced by vaccination against HBV infection is indicative of long-term vaccine efficacy [2]. We have identified significant haplotype effects with peak HBV vaccine-induced antibody level in five genes (*CD44*, *CD58*, *CDC42*, *IL19* and *IL1R1*) in a sample cohort of infant vaccinees (N = 651) from The Gambia. These analyses were adjusted for measurement time (between last vaccination and peak anti-HBs measurement) and vaccine group, which are known to affect peak anti-HBs [2], [17]. The majority of these effects were found in the separate analyses of unrelated and family data. We previously reported correlations in single SNPs and multi-marker analyses within *CD44*, *CD58*, IFNG, MAPK8, IL10RA and to a lesser extent ITGAL in the same study population. Our current analyses show that haplotypes can detect associations with genes that single SNP analysis cannot, supporting the original motivation for undertaking these analyses.

There are two differences in our current haplotype analysis compared to our earlier single SNP and multi-marker logistic regression models: First, in the present analysis we adjusted for measurement time (inverse relationship) and vaccine groups (six regimes since 1984) only because these factors exert strong effects on vaccine-induced antibody level [2]. We did not, as previously done, adjust for number of doses, age group, sex and village; however, these covariates do not affect peak anti-HBs level, except for a borderline effect of village. We thus deemed it unnecessary to include these factors in our adjusted haplotype analysis. Secondly, our haplotype analysis was run separately for unrelated and family data initially; these two samples were then also analyzed together (combined analysis), thus mirroring the single locus analysis. In general, the global tests for the combined analysis reflected the results obtained for the unrelated and family data, indicating that combining unrelated and family data in the same model is appropriate. In addition, our current results were corrected for multiple testing, using FDR.

Although we did find strong associations with the two genes (*CD44* and *CD58*) that were also detected with single SNPs, we found additional genes for which single SNP analyses failed to detect associations. CD44 is a cell-surface glycoprotein which affects IFNγ expression that is involved in a wide variety of cellular functions including lymphocyte activation, recirculation and homing. A recent study in HBV transgenic and knockout mice reported that CD44 affects the interaction between liver sinus endothelial cells and cytotoxic T lymphocytes as well as IFNγ levels [12]. Immune responses to HBV vaccination may therefore be altered by variation in the *CD44* gene. Our three-SNP haplotype association (rs353644-rs353630-rs7937602, Table 1) confirms earlier findings of two individual SNPs (rs353644 and rs7937602, Table S1) and was more significant than the single locus associations, indicating that the haplotype is a better predictor for peak anti-HBs level than the single markers alone. Similarly, two previous single locus associations (rs1414275 and rs1016140, Table S1) between variation in *CD58* (LFA3) and peak anti-HBs level were confirmed in the haplotype analysis, comprising three SNPs (rs1414275-rs11588376-rs1016140, Table 2). CD58, together with other T cell co-stimulatory molecules, has been shown to increase the quantity and magnitude in the avidity of T cells in recombinant poxvirus vaccine models in mice [25]. It is not clear whether these effects were due to the generation of more effector cells from naive T cell populations, the expansion of memory T cell populations, or both. Comparable responses may occur in response to HBV vaccination, and it is possible that *CD58* gene variation reduces such responses, in line with our observation of consistently decreased peak anti-HBs level in carriers of the variant alleles for the associated SNPs. We should point out that effects due to haplotype and single SNPs are not identical because the increases or decreases in peak anti-HBs for haplotypes is described relative to a referent haplotype. Therefore, directionality of the association may differ from the results for the associated allele at a single SNP because different effective referents are used.

Our previous single locus analyses (Table S1) showed no (*CDC42*) or merely trends of association (*IL19* and *IL1R1*) with level of peak anti-HBs. The haplotype analysis was thus able to detect significant novel associations for these three genes with vaccine-induced antibody level. CDC42 is a Rho GTPase that regulates signalling pathways and thus cellular functions, including cell morphology, migration, endocytosis and cell cycle progression. Activation/blocking of Rho GTPases, including CDC42, appears to affect infectivity, replication and possible metastatic effects of HBV [26] and HCV [27]. Our results suggest that there may be subtle modulation of HBV vaccine-induced immunity based on two polymorphisms (rs2056974-rs2473316) in *CDC42*. A homologue of IL10, IL19, is a key immune-regulatory cytokine, which activates monocytes to release IL6 and TNFα; it can promote Th2 immune deviation through a positive feedback loop resulting in more IL4- and fewer IFNγ-producing cells. It's own production is regulated by IFNγ [28]. Our haplotype results imply that the three-SNP haplotype rs12409415-rs2056225-rs2243158 confers protection by leading to an increased peak anti-HBs level. Finally, IL1R1 is a receptor for IL1α, IL1β and the IL1 receptor antagonist and thus involved in a multitude of cytokine-induced and inflammatory responses. Haplotypes in *IL1R1* have been correlated with fever after smallpox vaccination [29]. Whether this translates into alterations in vaccine-induced antibody level was not described. We did not see a clear pattern of increased or decreased anti-HBs level with a three-SNP haplotype (rs2287047-rs997049-rs3917299) and out of the five associated genes *IL1R1* showed the least significant associations.

Previous single locus associations in *MAPK8, IL10RA* and *IFNG* (and to a lesser extent *ITGAL*) were not confirmed in the haplotype analysis. It has been proposed that an analysis based on haplotype analysis is more powerful than that based on individual SNPs in the presence of multiple susceptibility alleles [19]. This argument may be supported by our data that provides evidence of association in some but not all of our haplotye analyses in these genes. For example, several haplotypes in IL10RA are at or near significant levels meeting our criteria, but all of the haplotypes showing suggestive evidence of haplotype association contain the marker rs4252279 (Table S3). This may indicate that only a single variant near that SNP affects anti-HBV level in response to vaccination [19].

All of our associated polymorphisms are either intronic or located in/near the 3′ or 5′ UTR regions, and are thus unlikely to be of direct relevance; for *CD44, CD58, CDC42, IL19* cluster together (see Table S1). In *IL1R1* the haplotype correlated with peak anti-HBs level spanned almost the whole length of the gene. This suggests that for the former four genes there may be a single functional variant located nearby, whereas for *IL1R1* there may be several polymorphisms that affect an increase or decrease of vaccine-induced antibody level. The possible functional implications of SNPs were assessed using freely available bioinformatics tools (http://snpinfo.niehs.nih.gov/snpfunc.htm and (http://fastsnp.ibms.sinica.edu.tw/pages/input_CandidateGeneSearch.jsp). None of the associated SNPs appears to affect transcription factor binding sites, splicing, introduce a stop codon or similar. The only exception was *CD44* rs1016140 which lies in a transcription factor-binding site and thus could affect gene expression. This emphasizes the assumption that functional variants were not genotyped, but are in LD with associated haplotypes.

To see if there is a common thread across the five genes associated with anti-HBs in this haplotype-based analysis, we used a simple bioinformatics tool, PubGene (www.pubgene.org) that searches PubMed for literature based on a single reference gene and identifies other genes that are found in conjunction with it in the literature. Of note, four of the genes we identified in our haplotype analysis, *CD44*, *IL19*, *IL1R1* and *CD58*, all have one other gene that is frequently co-referenced – IFNγ. This may suggest that the effects of these genes are mediated via a common pathway, although we recognize that this approach is highly biased by the current state of the literature and potentially by our candidate gene selection comprising genes implicated in immune-regulatory processes with the inclusion of gene families and coverage of regulatory pathways. Also, we found that at least one of the genes identified in the single SNP analyses, ITGAL, is associated with CD58 in the literature.

The only other larger scale report investigating candidate SNPs/genes (mostly non-HLA) in relation to HBV vaccine response was published recently [16]. They reported on associations with SNPs in BTNL2, C5, CCL15, FOXP1, HLA-B, HLA-DRA, HLA-DRQB1, IL6ST, KLRF1, LILRB4, LY6H, MBL2, TGFB2, TGFB3, and TNFSF15. There are significant differences between our and this recent study: The population background (Gambians compared to Indonesians), the timing of anti-HBs level measurement (median 9 weeks (i.e. peak) compared to 6 months post vaccination), the number of doses of vaccine administered (mostly 3 or 4 compared to 2 doses), the outcome measures (peak anti-HBs level quantitatively compared to vaccine responders/non-responders categorised into those with anti-HBs >100IU/l or <10IU/l), and the age at vaccination (infancy compared to >5 years). Therefore, a direct comparison is not possible. However, we repeated the above exercise of searching the literature for links between genes flagged up in our first report (MAPK8, ITGAL, IL10RA, IFNG, CD44, CD58) [17], this current haplotype report (CD44, IL19, IL1R1, CD58, CDC42) and the study by Davila *et al* (see gene list above) [16]. We noted that each of these genes (with exception of BTNL2 and KLRF1) has been co-referenced with one or more of the others and IFNG remains the strongest link, followed by CD44 (see Figure S2. Although this analysis is only based on a literature search, it serves to reinforce the idea that several genes affecting HBV vaccine-induced immunity fall into a common pathway. It would be interesting to see whether screening a larger number of IFNG SNPs (associated in both Gambians and Indonesians in the first stage analyses) and using an anti-HBs level in a more comparable manner might shed more light on the possible effect host genetic variation in this gene on HBV vaccine-induced antibody level. Similarly, a further screen including the hits from the Indonesian study not previously screened in Gambians would be warranted.

In conclusion, in this current analysis we identified haplotype associations with peak HBV vaccine-induced antibody level in *CD44* and *CD58* that include individual SNPs previously identified to associate with this outcome. Additionally, haplotypes in *CDC42*, *IL19* and *IL1R1* associated with peak anti-HBs level. These haplotypes did not include SNPs previously identified to associate with peak anti-HBs level in a single locus analysis at the GMT >1.5 or <0.6 and p≤0.001 level. Therefore, novel associations with HBV vaccine-induced immunity that would have been missed with the traditional single locus analysis have been identified in this haplotype screen. Biological mechanisms and pathways underlying immune-responses induced by vaccination identified through genetic association studies (e.g. possibly IFNγ associated molecules in our example), will ultimately help to further the development of vaccines and reduce disease burden globally.

## Supporting Information

Figure S1 Graphical representation of LD and Haplotype associations with anti-HBs level (unadjusted analysis) Solid lines indicate significant (p<0.05) global and individual haplotype associations with anti-HBs levels. Dotted lines indicate a significant global association but no individual haplotypic effects. Color of line denotes which study the significant association occurred in: black for the unrelated data, green for the family data and red for the combined data (unrelated and family together). The measure of LD employed was r2. Associated genes: A) CD44, B) CD58 C) CDC42, D) IL19 and E) IL1R1.
(0.72 MB TIF)

Figure S2 Graphical representation of bionetwork of candidate genes associated with HBV vaccine-induced antibody level in our previous analysis (Hennig et al 2008) and the current haplotype analysis in Gambians or the report by Davila et al 2010 in Indonesians. A simple bioinformatics tool, PubGene (www.pubgene.org), was used to search PubMed based on reference genes and identifying other genes that are found in conjunction with it in the literature. All candidate genes were shown to be linked in the literature with exception of BTNL2 and KLRF1. IFNG and CD44 were found to have the highest number of co-references with other candidate genes associated with anti-HBs. A) Centered on IFNG (connection count 26, article count 49278), B) Centered on CD44 (connection count 17, article count 6717). Hennig BJ, Fielding K, Broxholme J, Diatta M, Mendy M, Moore C et al. Host genetic factors and vaccine-induced immunity to hepatitis B virus infection. PLoS ONE 2008; 3(3): e1898. Davila S, Froeling FE, Tan A, Bonnard C, Boland GJ, et al. (2010) New genetic associations detected in a host response study to hepatitis B vaccine. Genes Immun. 2010 Apr;11(3):232-8.
(0.92 MB TIF)

Table S1 Single SNP analysis results for CD44, CD58, CDC42, IL19 and IL1R1 (adjusted for measurement time and vaccine group only).
(0.06 MB DOC)

Table S2 Adjusted § global p-values for five genes (CD44, CD58, CDC42, IL19, IL1R1) that were followed up in the haplotype analysis.
(0.13 MB DOC)

Table S3 Global unadjusted haplotype associations with anti-HBs level in unrelated, family and combined data for all 117 genes assessed as part of the haplotype analysis.
(1.15 MB DOC)
