# Correction: Host Genetic Factors and Vaccine-Induced Immunity to HBV Infection: Haplotype Analysis

**DOI:** 10.1371/annotation/11e67751-207c-4313-b239-3dd32d8ac126

**Published:** 2011-02-25

**Authors:** Kelli K. Ryckman, Katherine Fielding, Adrian V. Hill, Maimuna Mendy, Pura Rayco-Solon, Giorgio Sirugo, Marianne A. van der Sande, Pauline Waight, Hilton C. Whittle, Andrew J. Hall, Scott M. Williams, Branwen J. Hennig

The following information was missing from the Funding section: "The study was supported by funding from the NIHR Oxford Biomedical Research Centre programme."

