# Supplementary figures and images for: Host Genetic Factors and Vaccine-Induced Immunity to HBV Infection: Haplotype Analysis
Source: PLoS One. 2010 Aug 18;5(8):e12273. doi: 10.1371/journal.pone.0012273 (PMC2923624; doi:10.1371/journal.pone.0012273)

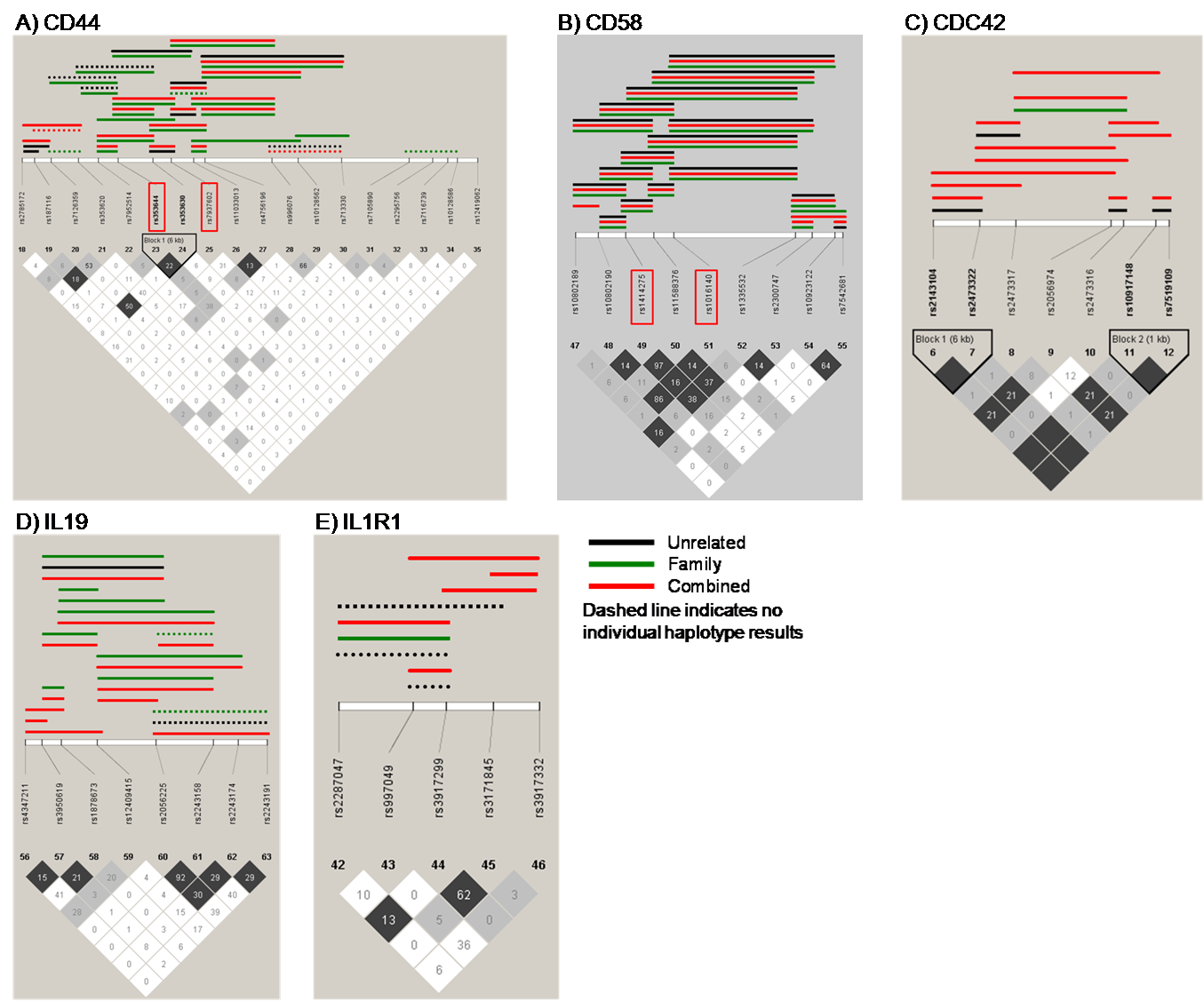

Supplement: Figure S1 — Graphical representation of LD and Haplotype associations with anti-HBs level (unadjusted analysis) Solid lines indicate significant (p<0.05) global and individual haplotype associations with anti-HBs levels. Dotted lines indicate a significant global association but no individual haplotypic effects. Color of line denotes which study the significant association occurred in: black for the unrelated data, green for the family data and red for the combined data (unrelated and family together). The measure of LD employed was r2. Associated genes: A) CD44, B) CD58 C) CDC42, D) IL19 and E) IL1R1. (0.72 MB TIF) [file pone.0012273.s001.tif]

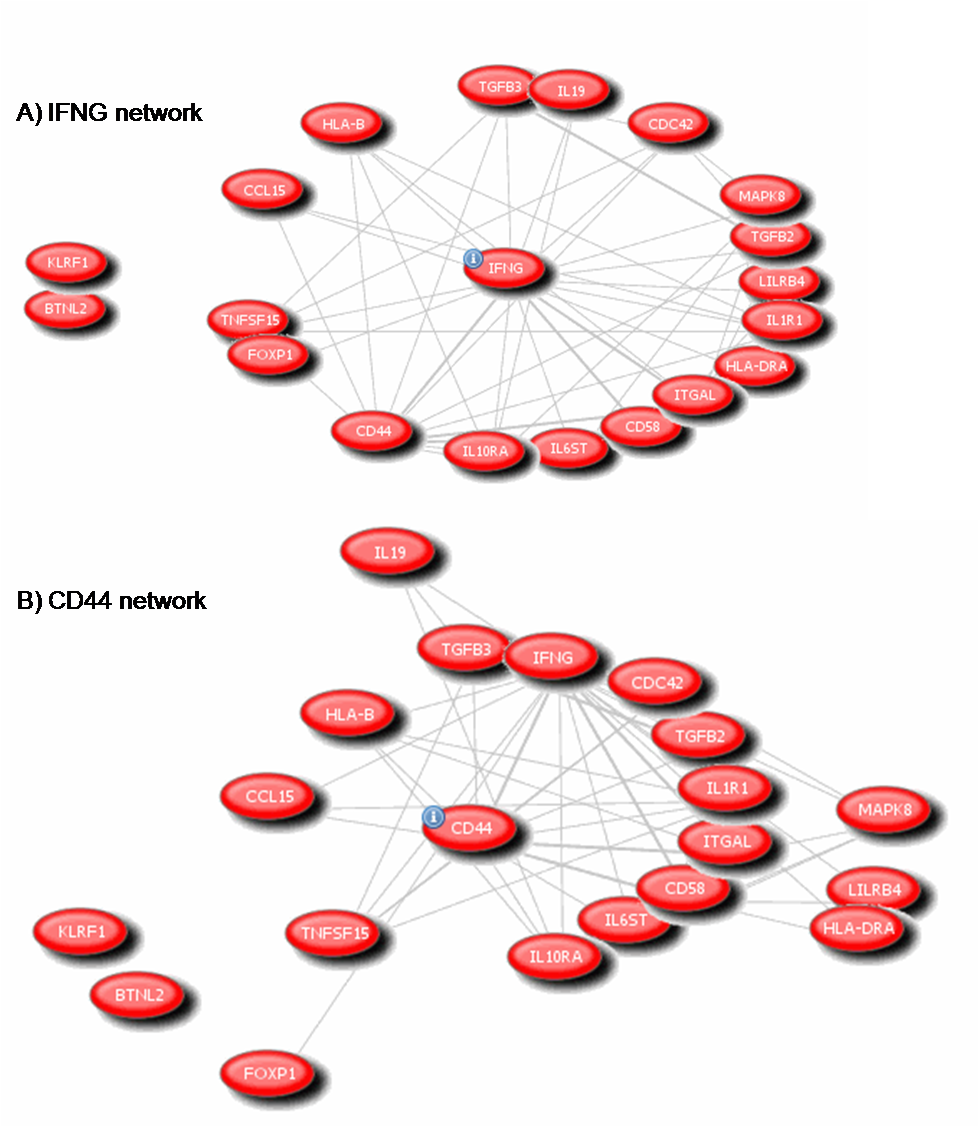

Supplement: Figure S2 — Graphical representation of bionetwork of candidate genes associated with HBV vaccine-induced antibody level in our previous analysis (Hennig et al 2008) and the current haplotype analysis in Gambians or the report by Davila et al 2010 in Indonesians. A simple bioinformatics tool, PubGene (www.pubgene.org), was used to search PubMed based on reference genes and identifying other genes that are found in conjunction with it in the literature. All candidate genes were shown to be linked in the literature with exception of BTNL2 and KLRF1. IFNG and CD44 were found to have the highest number of co-references with other candidate genes associated with anti-HBs. A) Centered on IFNG (connection count 26, article count 49278), B) Centered on CD44 (connection count 17, article count 6717). Hennig BJ, Fielding K, Broxholme J, Diatta M, Mendy M, Moore C et al. Host genetic factors and vaccine-induced immunity to hepatitis B virus infection. PLoS ONE 2008; 3(3): e1898. Davila S, Froeling FE, Tan A, Bonnard C, Boland GJ, et al. (2010) New genetic associations detected in a host response study to hepatitis B vaccine. Genes Immun. 2010 Apr;11(3):232-8. (0.92 MB TIF) [file pone.0012273.s002.tif]
