# Supplementary material for: Host Genetic Factors and Vaccine-Induced Immunity to HBV Infection: Haplotype Analysis
Source: PLoS One. 2010 Aug 18;5(8):e12273. doi: 10.1371/journal.pone.0012273 (PMC2923624; doi:10.1371/journal.pone.0012273)
Supplement: Table S1 — Single SNP analysis results for CD44, CD58, CDC42, IL19 and IL1R1 (adjusted for measurement time and vaccine group only). (0.06 MB DOC) [file pone.0012273.s003.doc]

| **SNP_ID** | **Location** | **ID in Hennig et al 2008** | **Gene** | **GMTratio** | **P-value** | **95%CU** | |
| --- | --- | --- | --- | --- | --- | --- | --- |
| **rs353644** | intron 1 | s440_12 | CD44 | **1.71** | **0.001** | 1.25 | 2.34 |
| **rs353644** |  | s440_22 | CD44 | 0.87 | 0.832 | 0.24 | 3.15 |
| **rs353630** | intron 1 | s441_12 | CD44 | **1.58** | **0.026** | 1.06 | 2.37 |
| **rs353630** |  | s441_22 | CD44 | 1.28 | 0.321 | 0.79 | 2.06 |
| **rs7937602** | intron 1 | s442gp_33 | CD44 | **0.58** | **0.005** | 0.40 | 0.85 |
| **rs1414275** | intron 3 | s062_12 | CD58 | **0.51** | **6.89E-05** | 0.37 | 0.71 |
| **rs1414275** |  | s062_22 | CD58 | **0.45** | **3.33E-04** | 0.29 | 0.69 |
| **rs11588376** | intron 3 | s063_12 | CD58 | **0.51** | **7.94E-05** | 0.37 | 0.71 |
| **rs11588376** |  | s063_22 | CD58 | **0.46** | **6.14E-04** | 0.29 | 0.71 |
| **rs1016140** | intron 3 | s064gp_33 | CD58 | **0.42** | **2.44E-05** | 0.28 | 0.62 |
| **rs2473316** | intron 3 | s023_12 | CDC42 | 1.07 | 0.758 | 0.71 | 1.62 |
| **rs2473316** |  | s023_22 | CDC42 | 0.91 | 0.893 | 0.22 | 3.82 |
| **rs10917148** | intron 5 | s024_12 | CDC42 | 0.81 | 0.391 | 0.49 | 1.32 |
| **rs10917148** |  | s024_22 | CDC42 | 0.08 | 0.059 | 0.01 | 1.10 |
| **rs12409415** | 5' near gene | s118_12 | IL19 | 1.17 | 0.328 | 0.86 | 1.59 |
| **rs12409415** |  | s118_22 | IL19 | **2.52** | **0.025** | 1.12 | 5.64 |
| **rs2056225** | 5' near gene | s119_12 | IL19 | 1.11 | 0.561 | 0.78 | 1.57 |
| **rs2056225** |  | s119_22 | IL19 | 0.74 | 0.456 | 0.34 | 1.63 |
| **rs2243158** | 5'UTR | s120_12 | IL19 | 1.08 | 0.652 | 0.76 | 1.55 |
| **rs2243158** |  | s120_22 | IL19 | 0.79 | 0.580 | 0.34 | 1.84 |
| **rs2287047** | intron 1 | s145_12 | IL1R1 | 0.91 | 0.604 | 0.64 | 1.29 |
| **rs2287047** |  | s145_22 | IL1R1 | 0.77 | 0.307 | 0.46 | 1.28 |
| **rs997049** | intron 4 | s146_12 | IL1R1 | 1.19 | 0.370 | 0.81 | 1.74 |
| **rs997049** |  | s146_22 | IL1R1 | 1.49 | 0.465 | 0.51 | 4.31 |
| **rs3917299** | intron 6 | s147_12 | IL1R1 | 0.82 | 0.318 | 0.56 | 1.21 |
| **rs3917299** |  | s147_22 | IL1R1 | 1.26 | 0.577 | 0.56 | 2.82 |
| **rs3171845** | intron 9 | s148_12 | IL1R1 | 0.74 | 0.091 | 0.53 | 1.05 |
| **rs3171845** |  | s148_22 | IL1R1 | 0.68 | 0.259 | 0.35 | 1.33 |
| **rs3917332** | 3' near gene | s149_12 | IL1R1 | 1.07 | 0.762 | 0.69 | 1.66 |
| **rs3917332** |  | s149_22 | IL1R1 | **2.87** | **0.012** | 1.26 | 6.56 |

Table S1 shows the single SNP analysis results for all markers found to associate with peak vaccine-induced antibody (anti-HBs) level in the current haplotype analysis for measurement time and vaccine group only. These data are shown for ease of direct comparison, as our previous analysis was additionally adjusted for number of doses of vaccine, age group, sex and village (Hennig *et al* 2008, for details see table 3 and table S2). Note: The genotype/allele designations are 1 for ancestral and 2 for variant allele; _33 indicates the grouping of homozygote variants (22) and heterozygotes (12).

Hennig BJ, Fielding K, Broxholme J, Diatta M, Mendy M, Moore C et al. Host genetic factors and vaccine-induced immunity to hepatitis B virus infection. PLoS ONE 2008; 3(3): e1898.
