# Supplementary material for: Host Genetic Factors and Vaccine-Induced Immunity to HBV Infection: Haplotype Analysis
Source: PLoS One. 2010 Aug 18;5(8):e12273. doi: 10.1371/journal.pone.0012273 (PMC2923624; doi:10.1371/journal.pone.0012273)
Supplement: Table S2 — Adjusted § global p-values for five genes (CD44, CD58, CDC42, IL19, IL1R1) that were followed up in the haplotype analysis. (0.13 MB DOC) [file pone.0012273.s004.doc]

| **Gene** | **SNP IDs in haplotype** | **Haplotype ID in Hennig et al 2008** | **Data set** | | | |
| --- | --- | --- | --- | --- | --- | --- |
|  |  |  | **unrelated** | | **family** | **combined** |
| CD44 | rs2785172rs187116 | s435s436 | 0.006 | 1.000 | | 0.252 |
| CD44 | rs2785172rs187116rs7126359 | s435s436s437 | 0.045 | 0.267 | | 0.495 |
| CD44 | rs2785172rs187116rs7126359rs353620 | s435s436s437s438 | 0.850 | 0.841 | | 0.552 |
| CD44 | rs187116rs7126359 | s436s437 | 0.117 | 0.587 | | 1.000 |
| CD44 | rs187116rs7126359rs353620 | s436s437s438 | 0.143 | 0.572 | | 0.000 |
| CD44 | rs187116rs7126359rs353620rs7952514 | s436s437s438s439 | 0.020 | 1.000 | | 0.122 |
| CD44 | rs7126359rs353620 | s437s438 | 0.281 | 1.000 | | 1.000 |
| CD44 | rs7126359rs353620rs7952514 | s437s438s439 | 0.326 | 0.001 | | 0.377 |
| CD44 | rs7126359rs353620rs7952514rs353644 | s437s438s439s440 | 0.000 | 0.000 | | 1.000 |
| CD44 | rs353620rs7952514 | s438s439 | 0.121 | 0.710 | | 0.428 |
| CD44 | rs353620rs7952514rs353644 | s438s439s440 | 0.001 | 0.314 | | 0.707 |
| CD44 | rs353620rs7952514rs353644rs353630 | s438s439s440s441 | 0.665 | 1.000 | | 0.213 |
| CD44 | rs7952514rs353644 | s439s440 | 0.519 | 0.231 | | 0.008 |
| CD44 | rs7952514rs353644rs353630 | s439s440s441 | 0.724 | 0.000 | | 0.038 |
| CD44 | rs7952514rs353644rs353630rs7937602 | s439s440s441s442 | 0.019 | 0.000 | | 0.008 |
| CD44 | rs353644rs353630 | s440s441 | 0.082 | 0.113 | | 0.000 |
| CD44 | rs353644rs353630rs7937602 | s440s441s442 | 0.002 | 1.000 | | 0.000 |
| CD44 | rs353644rs353630rs7937602rs11033013 | s440s441s442s443 | 0.058 | 0.000 | | 1.000 |
| CD44 | rs353630rs7937602 | s441s442 | 0.001 | 0.049 | | 0.645 |
| CD44 | rs353630rs7937602rs11033013 | s441s442s443 | 0.414 | 0.065 | | 1.000 |
| CD44 | rs353630rs7937602rs11033013rs4756196 | s441s442s443s444 | 0.000 | 0.000 | | 0.021 |
| CD44 | rs7937602rs11033013 | s442s443 | 0.288 | 0.011 | | 0.000 |
| CD44 | rs7937602rs11033013rs4756196 | s442s443s444 | 0.320 | 0.000 | | 0.001 |
| CD44 | rs7937602rs11033013rs4756196rs996076 | s442s443s444s445 | 0.005 | 1.000 | | 0.000 |
| CD44 | rs11033013rs4756196 | s443s444 | 1.000 | 1.000 | | 1.000 |
| CD44 | rs11033013rs4756196rs996076 | s443s444s445 | 0.070 | 0.335 | | 0.251 |
| CD44 | rs11033013rs4756196rs996076rs10128562 | s443s444s445s446 | 0.486 | 0.013 | | 1.000 |
| CD44 | rs4756196rs996076 | s444s445 | 0.489 | 0.774 | | 0.866 |
| CD44 | rs4756196rs996076rs10128562 | s444s445s446 | 1.000 | 1.000 | | 1.000 |
| CD44 | rs4756196rs996076rs10128562rs713330 | s444s445s446s447 | 0.016 | 0.127 | | 0.001 |
| CD44 | rs996076rs10128562 | s445s446 | 1.000 | 0.007 | | 0.054 |
| CD44 | rs996076rs10128562rs713330 | s445s446s447 | 0.401 | 1.000 | | 0.082 |
| CD44 | rs996076rs10128562rs713330rs7105890 | s445s446s447s448 | 0.227 | 1.000 | | 1.000 |
| CD44 | rs10128562rs713330 | s446s447 | 0.740 | 1.000 | | 1.000 |
| CD44 | rs10128562rs713330rs7105890 | s446s447s448 | 0.762 | 0.002 | | 0.021 |
| CD44 | rs10128562rs713330rs7105890rs2295756 | s446s447s448s449 | 1.000 | 0.016 | | 0.000 |
| CD44 | rs713330rs7105890 | s447s448 | 0.605 | 1.000 | | 0.000 |
| CD44 | rs713330rs7105890rs2295756 | s447s448s449 | 0.018 | 0.000 | | 0.020 |
| CD44 | rs713330rs7105890rs2295756rs7116739 | s447s448s449s450 | 0.004 | 1.000 | | 1.000 |
| CD44 | rs7105890rs2295756 | s448s449 | 0.174 | 0.020 | | 1.000 |
| CD44 | rs7105890rs2295756rs7116739 | s448s449s450 | 1.000 | 1.000 | | 1.000 |
| CD44 | rs7105890rs2295756rs7116739rs10128586 | s448s449s450s451 | 1.000 | 0.004 | | 1.000 |
| CD44 | rs2295756rs7116739 | s449s450 | 0.004 | 0.004 | | 0.751 |
| CD44 | rs2295756rs7116739rs10128586 | s449s450s451 | 0.084 | 1.000 | | 0.128 |
| CD44 | rs2295756rs7116739rs10128586rs12419062 | s449s450s451s452 | 0.009 | 0.000 | | 0.000 |
| CD44 | rs7116739rs10128586 | s450s451 | 0.704 | 0.611 | | 1.000 |
| CD44 | rs7116739rs10128586rs12419062 | s450s451s452 | 0.929 | 1.000 | | 0.229 |
| CD44 | rs10128586rs12419062 | s451s452 | 0.412 | 0.029 | | 0.016 |
| CD58 | rs10802189rs10802190 | s060s061 | 0.237 | 0.005 | | 0.468 |
| CD58 | rs10802189rs10802190rs1414275 | s060s061s062 | 0.021 | 0.246 | | 1.000 |
| CD58 | rs10802189rs10802190rs1414275rs11588376 | s060s061s062s063 | 0.095 | 0.246 | | 1.000 |
| CD58 | rs10802190rs1414275 | s061s062 | 0.325 | 0.067 | | 1.000 |
| CD58 | rs10802190rs1414275rs11588376 | s061s062s063 | 0.293 | 0.212 | | 0.802 |
| CD58 | rs10802190rs1414275rs11588376rs1016140 | s061s062s063s064 | 0.021 | 1.000 | | 0.092 |
| CD58 | rs1414275rs11588376 | s062s063 | 0.047 | 0.146 | | 0.008 |
| CD58 | rs1414275rs11588376rs1016140 | s062s063s064 | 0.023 | 0.043 | | 0.008 |
| CD58 | rs1414275rs11588376rs1016140rs1335532 | s062s063s064s065 | 1.000 | 1.000 | | 0.111 |
| CD58 | rs11588376rs1016140 | s063s064 | 0.019 | 0.076 | | 0.084 |
| CD58 | rs11588376rs1016140rs1335532 | s063s064s065 | 0.009 | 1.000 | | 0.002 |
| CD58 | rs11588376rs1016140rs1335532rs2300747 | s063s064s065s066 | 0.721 | 1.000 | | 1.000 |
| CD58 | rs1016140rs1335532 | s064s065 | 0.070 | 0.410 | | 0.000 |
| CD58 | rs1016140rs1335532rs2300747 | s064s065s066 | 1.000 | 0.000 | | 0.780 |
| CD58 | rs1016140rs1335532rs2300747rs10923122 | s064s065s066s067 | 0.319 | 1.000 | | 0.726 |
| CD58 | rs1335532rs2300747 | s065s066 | 0.002 | 1.000 | | 1.000 |
| CD58 | rs1335532rs2300747rs10923122 | s065s066s067 | 0.289 | 0.001 | | 0.112 |
| CD58 | rs1335532rs2300747rs10923122rs7542681 | s065s066s067s068 | 0.114 | 0.000 | | 1.000 |
| CD58 | rs2300747rs10923122 | s066s067 | 0.010 | 1.000 | | 0.806 |
| CD58 | rs2300747rs10923122rs7542681 | s066s067s068 | 1.000 | 1.000 | | 0.634 |
| CD58 | rs10923122rs7542681 | s067s068 | 1.000 | 0.123 | | 1.000 |
| CDC42 | rs2143104rs2473322 | s019s020 | 1.000 | 0.113 | | 1.000 |
| CDC42 | rs2143104rs2473322rs2473317 | s019s020s021 | 0.063 | 1.000 | | 0.003 |
| CDC42 | rs2143104rs2473322rs2473317rs2056974 | s019s020s021s022 | 1.000 | 0.054 | | 1.000 |
| CDC42 | rs2473322rs2473317 | s020s021 | 0.988 | 0.006 | | 0.024 |
| CDC42 | rs2473322rs2473317rs2056974 | s020s021s022 | 1.000 | 1.000 | | 1.000 |
| CDC42 | rs2473322rs2473317rs2056974rs2473316 | s020s021s022s023 | 0.013 | 0.000 | | 0.002 |
| CDC42 | rs2473317rs2056974 | s021s022 | 0.412 | 0.900 | | 0.057 |
| CDC42 | rs2473317rs2056974rs2473316 | s021s022s023 | 0.057 | 1.000 | | 0.010 |
| CDC42 | rs2473317rs2056974rs2473316rs10917148 | s021s022s023s024 | 0.014 | 0.000 | | 0.018 |
| CDC42 | rs2056974rs2473316 | s022s023 | 0.007 | 0.004 | | 0.006 |
| CDC42 | rs2056974rs2473316rs10917148 | s022s023s024 | 0.053 | 0.000 | | 1.000 |
| CDC42 | rs2056974rs2473316rs10917148rs7519109 | s022s023s024s025 | 0.042 | 0.000 | | 0.042 |
| CDC42 | rs2473316rs10917148 | s023s024 | 0.106 | 0.178 | | 0.000 |
| CDC42 | rs2473316rs10917148rs7519109 | s023s024s025 | 0.038 | 0.178 | | 1.000 |
| CDC42 | rs10917148rs7519109 | s024s025 | 1.000 | 0.113 | | 1.000 |
| IL19 | rs4347211rs3950619 | s115s116 | 1.000 | 0.882 | | 0.146 |
| IL19 | rs4347211rs3950619rs1878673 | s115s116s117 | 0.144 | 0.076 | | 0.001 |
| IL19 | rs4347211rs3950619rs1878673rs12409415 | s115s116s117s118 | 0.782 | 0.006 | | 1.000 |
| IL19 | rs3950619rs1878673 | s116s117 | 0.230 | 0.677 | | 0.035 |
| IL19 | rs3950619rs1878673rs12409415 | s116s117s118 | 0.040 | 0.876 | | 0.001 |
| IL19 | rs3950619rs1878673rs12409415rs2056225 | s116s117s118s119 | 0.000 | 1.000 | | 0.003 |
| IL19 | rs1878673rs12409415 | s117s118 | 0.024 | 1.000 | | 0.258 |
| IL19 | rs1878673rs12409415rs2056225 | s117s118s119 | 0.026 | 0.000 | | 0.869 |
| IL19 | rs1878673rs12409415rs2056225rs2243158 | s117s118s119s120 | 0.049 | 0.003 | | 0.000 |
| IL19 | rs12409415rs2056225 | s118s119 | 0.007 | 0.000 | | 0.038 |
| IL19 | rs12409415rs2056225rs2243158 | s118s119s120 | 0.008 | 0.000 | | 0.004 |
| IL19 | rs12409415rs2056225rs2243158rs2243174 | s118s119s120s121 | 0.035 | 0.000 | | 1.000 |
| IL19 | rs2056225rs2243158 | s119s120 | 0.318 | 0.007 | | 0.000 |
| IL19 | rs2056225rs2243158rs2243174 | s119s120s121 | 0.717 | 1.000 | | 0.003 |
| IL19 | rs2056225rs2243158rs2243174rs2243191 | s119s120s121s122 | 1.000 | 0.000 | | 1.000 |
| IL19 | rs2243158rs2243174 | s120s121 | 1.000 | 1.000 | | 0.221 |
| IL19 | rs2243158rs2243174rs2243191 | s120s121s122 | 0.012 | 0.727 | | 1.000 |
| IL19 | rs2243174rs2243191 | s121s122 | 1.000 | 0.113 | | 0.176 |
| IL1R1 | rs2287047rs997049 | s145s146 | 0.252 | 0.036 | | 1.000 |
| IL1R1 | rs2287047rs997049rs3917299 | s145s146s147 | 0.848 | 0.021 | | 0.020 |
| IL1R1 | rs2287047rs997049rs3917299rs3171845 | s145s146s147s148 | 0.376 | 1.000 | | 0.284 |
| IL1R1 | rs997049rs3917299 | s146s147 | 0.286 | 1.000 | | 1.000 |
| IL1R1 | rs997049rs3917299rs3171845 | s146s147s148 | 0.002 | 0.417 | | 1.000 |
| IL1R1 | rs997049rs3917299rs3171845rs3917332 | s146s147s148s149 | 0.231 | 0.567 | | 1.000 |
| IL1R1 | rs3917299rs3171845 | s147s148 | 0.512 | 0.331 | | 0.396 |
| IL1R1 | rs3917299rs3171845rs3917332 | s147s148s149 | 0.222 | 0.441 | | 0.247 |
| IL1R1 | rs3171845rs3917332 | s148s149 | 0.239 | 0.323 | | 1.000 |

^§^ Adjusted for measurement time (between last vaccination and peak antibody level assessment) and vaccine group (six regimes since 1984)
