# Supplementary material for: Host Genetic Factors and Vaccine-Induced Immunity to HBV Infection: Haplotype Analysis
Source: PLoS One. 2010 Aug 18;5(8):e12273. doi: 10.1371/journal.pone.0012273 (PMC2923624; doi:10.1371/journal.pone.0012273)
Supplement: Table S3 — Global unadjusted haplotype associations with anti-HBs level in unrelated, family and combined data for all 117 genes assessed as part of the haplotype analysis. (1.15 MB DOC) [file pone.0012273.s005.doc]

|  |  |  | Global p-values | | |
| --- | --- | --- | --- | --- | --- |
| Gene | Haplotype (rs#'s) | Haplotype (SNP id's) | unrelated | family | combined |
| AICDA | rs11046349rs2580876 | s505s506 | 0.553 | 0.345 | 0.649 |
| AKT1 | rs2494731rs2494743 | s569s570 | 0.710 | 0.458 | 0.252 |
| AKT1 | rs2494731rs2494743rs1130214 | s569s570s571 | 0.520 | 0.258 | 0.172 |
| AKT1 | rs2494743rs1130214 | s570s571 | 0.378 | 0.011 | 0.521 |
| BCL6 | rs1523475rs1523474 | s238s239 | 0.201 | 0.255 | 0.277 |
| BCL6 | rs1523475rs1523474rs4686467 | s238s239s240 | 0.151 | 0.127 | 0.083 |
| BCL6 | rs1523474rs4686467 | s239s240 | 0.167 | 0.095 | 0.032 |
| BLR1 | rs523604rs1623316 | s485s486 | 0.214 | 0.912 | 0.394 |
| BLR1 | rs523604rs1623316rs613791 | s485s486s487 | 0.537 | 1.000 | 0.571 |
| BLR1 | rs523604rs1623316rs613791rs3922 | s485s486s487s488 | 0.465 | 0.565 | 0.928 |
| BLR1 | rs1623316rs613791 | s486s487 | 0.440 | 0.650 | 0.644 |
| BLR1 | rs1623316rs613791rs3922 | s486s487s488 | 0.600 | 0.566 | 0.608 |
| BLR1 | rs613791rs3922 | s487s488 | 0.264 | 0.193 | 0.375 |
| C3 | rs344555rs344550 | s637s638 | 0.224 | 0.350 | 0.541 |
| C3 | rs344555rs344550rs237554 | s637s638s639 | 0.069 | 0.108 | 0.133 |
| C3 | rs344555rs344550rs237554rs432001 | s637s638s639s640 | 0.813 | 0.027 | 0.130 |
| C3 | rs344550rs237554 | s638s639 | 0.534 | 0.160 | 0.086 |
| C3 | rs344550rs237554rs432001 | s638s639s640 | 0.616 | 0.084 | 0.156 |
| C3 | rs344550rs237554rs432001rs2287846 | s638s639s640s641 | 0.661 | 0.441 | 0.547 |
| C3 | rs237554rs432001 | s639s640 | 0.551 | 0.024 | 0.216 |
| C3 | rs237554rs432001rs2287846 | s639s640s641 | 0.712 | 8.1E-05 | 0.139 |
| C3 | rs237554rs432001rs2287846rs366510 | s639s640s641s642 | 0.857 | 0.114 | 0.311 |
| C3 | rs432001rs2287846 | s640s641 | 0.384 | 0.090 | 0.368 |
| C3 | rs432001rs2287846rs366510 | s640s641s642 | 0.463 | 0.056 | 0.199 |
| C3 | rs432001rs2287846rs366510rs432823 | s640s641s642s643 | 0.495 | 0.070 | 0.202 |
| C3 | rs2287846rs366510 | s641s642 | 0.554 | 0.215 | 0.199 |
| C3 | rs2287846rs366510rs432823 | s641s642s643 | 0.582 | 0.221 | 0.207 |
| C3 | rs2287846rs366510rs432823rs2250656 | s641s642s643s644 | 0.698 | 0.408 | 0.727 |
| C3 | rs366510rs432823 | s642s643 | 0.298 | 0.452 | 0.186 |
| C3 | rs366510rs432823rs2250656 | s642s643s644 | 0.729 | 0.464 | 0.422 |
| C3 | rs432823rs2250656 | s643s644 | 0.813 | 0.423 | 0.417 |
| CARD8 | rs1062808rs1971783 | s664s665 | 0.899 | 0.498 | 0.889 |
| CARD8 | rs1062808rs1971783rs1966625 | s664s665s666 | 0.607 | 0.720 | 0.774 |
| CARD8 | rs1062808rs1971783rs1966625rs3786740 | s664s665s666s667 | 0.795 | 0.942 | 0.837 |
| CARD8 | rs1971783rs1966625 | s665s666 | 0.585 | 0.907 | 0.754 |
| CARD8 | rs1971783rs1966625rs3786740 | s665s666s667 | 0.795 | 0.944 | 0.847 |
| CARD8 | rs1971783rs1966625rs3786740rs2043211 | s665s666s667s668 | 0.799 | 0.300 | 0.889 |
| CARD8 | rs1966625rs3786740 | s666s667 | 0.609 | 0.873 | 0.781 |
| CARD8 | rs1966625rs3786740rs2043211 | s666s667s668 | 0.773 | 0.976 | 0.860 |
| CARD8 | rs1966625rs3786740rs2043211rs6509364 | s666s667s668s669 | 0.769 | 0.632 | 0.935 |
| CARD8 | rs3786740rs2043211 | s667s668 | 0.800 | 0.816 | 0.727 |
| CARD8 | rs3786740rs2043211rs6509364 | s667s668s669 | 0.779 | 0.969 | 0.922 |
| CARD8 | rs3786740rs2043211rs6509364rs6509368 | s667s668s669s670 | 0.593 | 0.883 | 0.978 |
| CARD8 | rs2043211rs6509364 | s668s669 | 0.413 | 0.943 | 0.444 |
| CARD8 | rs2043211rs6509364rs6509368 | s668s669s670 | 0.235 | 0.819 | 0.618 |
| CARD8 | rs2043211rs6509364rs6509368rs1972619 | s668s669s670s671 | 0.262 | 0.966 | 0.700 |
| CARD8 | rs6509364rs6509368 | s669s670 | 0.413 | 0.582 | 0.883 |
| CARD8 | rs6509364rs6509368rs1972619 | s669s670s671 | 0.502 | 0.320 | 0.528 |
| CARD8 | rs6509368rs1972619 | s670s671 | 0.564 | 0.482 | 0.378 |
| CD163 | rs11054130rs7487755 | s499s500 | 0.961 | 0.783 | 0.853 |
| CD163 | rs11054130rs7487755rs6488336 | s499s500s501 | 0.683 | 0.805 | 0.874 |
| CD163 | rs11054130rs7487755rs6488336rs6488338 | s499s500s501s502 | 0.756 | 0.933 | 0.512 |
| CD163 | rs7487755rs6488336 | s500s501 | 0.996 | 0.878 | 0.947 |
| CD163 | rs7487755rs6488336rs6488338 | s500s501s502 | 0.701 | 0.844 | 0.737 |
| CD163 | rs7487755rs6488336rs6488338rs6488340 | s500s501s502s503 | 0.485 | 0.006 | 0.343 |
| CD163 | rs6488336rs6488338 | s501s502 | 0.924 | 0.880 | 0.981 |
| CD163 | rs6488336rs6488338rs6488340 | s501s502s503 | 0.638 | 2.2E-04 | 0.958 |
| CD163 | rs6488336rs6488338rs6488340rs4883263 | s501s502s503s504 | 0.448 | 0.739 | 0.950 |
| CD163 | rs6488338rs6488340 | s502s503 | 0.186 | 0.867 | 0.608 |
| CD163 | rs6488338rs6488340rs4883263 | s502s503s504 | 0.098 | 0.716 | 0.829 |
| CD163 | rs6488340rs4883263 | s503s504 | 0.240 | 0.517 | 0.401 |
| CD22 | rs2239511rs2312586 | s653s654 | 0.956 | 0.123 | 0.377 |
| CD22 | rs2239511rs2312586rs10406069 | s653s654s655 | 0.113 | 0.093 | 0.405 |
| CD22 | rs2239511rs2312586rs10406069rs10423648 | s653s654s655s656 | 0.261 | 0.033 | 0.247 |
| CD22 | rs2312586rs10406069 | s654s655 | 0.410 | 1.000 | 0.303 |
| CD22 | rs2312586rs10406069rs10423648 | s654s655s656 | 0.168 | 0.005 | 0.003 |
| CD22 | rs10406069rs10423648 | s655s656 | 0.293 | 0.404 | 0.422 |
| CD28 | rs3181100rs1181390 | s197s198 | 0.654 | 0.987 | 0.774 |
| CD28 | rs3181100rs1181390rs10932017 | s197s198s199 | 0.815 | 0.666 | 0.952 |
| CD28 | rs3181100rs1181390rs10932017rs3769683 | s197s198s199s200 | 0.902 | 0.986 | 0.821 |
| CD28 | rs1181390rs10932017 | s198s199 | 0.751 | 0.775 | 0.994 |
| CD28 | rs1181390rs10932017rs3769683 | s198s199s200 | 0.817 | 0.821 | 0.753 |
| CD28 | rs1181390rs10932017rs3769683rs3116494 | s198s199s200s201 | 0.422 | 1.000 | 0.581 |
| CD28 | rs10932017rs3769683 | s199s200 | 0.319 | 0.719 | 0.536 |
| CD28 | rs10932017rs3769683rs3116494 | s199s200s201 | 0.654 | 0.795 | 0.720 |
| CD28 | rs3769683rs3116494 | s200s201 | 0.561 | 0.695 | 0.740 |
| CD44 | rs2785172rs187116 | s435s436 | 0.035 | 1.000 | 0.069 |
| CD44 | rs2785172rs187116rs7126359 | s435s436s437 | 0.018 | 1.000 | 0.016 |
| CD44 | rs2785172rs187116rs7126359rs353620 | s435s436s437s438 | 0.072 | 0.871 | 0.002 |
| CD44 | rs187116rs7126359 | s436s437 | 0.150 | 0.459 | 0.181 |
| CD44 | rs187116rs7126359rs353620 | s436s437s438 | 0.427 | 0.389 | 0.018 |
| CD44 | rs187116rs7126359rs353620rs7952514 | s436s437s438s439 | 0.592 | 1.000 | 0.223 |
| CD44 | rs7126359rs353620 | s437s438 | 0.428 | 1.0E-07 | 0.677 |
| CD44 | rs7126359rs353620rs7952514 | s437s438s439 | 0.861 | 0.588 | 0.381 |
| CD44 | rs7126359rs353620rs7952514rs353644 | s437s438s439s440 | 0.010 | 0.016 | 1.000 |
| CD44 | rs353620rs7952514 | s438s439 | 0.885 | 0.426 | 0.404 |
| CD44 | rs353620rs7952514rs353644 | s438s439s440 | 0.029 | 0.004 | 0.111 |
| CD44 | rs353620rs7952514rs353644rs353630 | s438s439s440s441 | 0.023 | 0.002 | 0.062 |
| CD44 | rs7952514rs353644 | s439s440 | 0.478 | 5.9E-04 | 0.005 |
| CD44 | rs7952514rs353644rs353630 | s439s440s441 | 0.325 | 0.002 | 0.024 |
| CD44 | rs7952514rs353644rs353630rs7937602 | s439s440s441s442 | 0.182 | 0.015 | 0.080 |
| CD44 | rs353644rs353630 | s440s441 | 0.297 | 3.3E-04 | 0.032 |
| CD44 | rs353644rs353630rs7937602 | s440s441s442 | 0.073 | 0.004 | 0.001 |
| CD44 | rs353644rs353630rs7937602rs11033013 | s440s441s442s443 | 0.047 | 1.7E-04 | 0.109 |
| CD44 | rs353630rs7937602 | s441s442 | 0.040 | 0.693 | 0.033 |
| CD44 | rs353630rs7937602rs11033013 | s441s442s443 | 0.055 | 0.417 | 0.109 |
| CD44 | rs353630rs7937602rs11033013rs4756196 | s441s442s443s444 | 0.064 | 0.001 | 0.019 |
| CD44 | rs7937602rs11033013 | s442s443 | 0.036 | 0.531 | 0.043 |
| CD44 | rs7937602rs11033013rs4756196 | s442s443s444 | 0.049 | 0.002 | 0.033 |
| CD44 | rs7937602rs11033013rs4756196rs996076 | s442s443s444s445 | 0.092 | 6.1E-05 | 0.029 |
| CD44 | rs11033013rs4756196 | s443s444 | 0.091 | 4.7E-04 | 0.027 |
| CD44 | rs11033013rs4756196rs996076 | s443s444s445 | 0.090 | 0.002 | 0.034 |
| CD44 | rs11033013rs4756196rs996076rs10128562 | s443s444s445s446 | 0.104 | 3.6E-05 | 0.063 |
| CD44 | rs4756196rs996076 | s444s445 | 0.076 | 0.001 | 0.035 |
| CD44 | rs4756196rs996076rs10128562 | s444s445s446 | 0.068 | 0.007 | 0.034 |
| CD44 | rs4756196rs996076rs10128562rs713330 | s444s445s446s447 | 0.010 | 0.014 | 0.004 |
| CD44 | rs996076rs10128562 | s445s446 | 0.530 | 0.461 | 0.958 |
| CD44 | rs996076rs10128562rs713330 | s445s446s447 | 0.006 | 0.250 | 0.030 |
| CD44 | rs996076rs10128562rs713330rs7105890 | s445s446s447s448 | 0.460 | 0.282 | 0.987 |
| CD44 | rs10128562rs713330 | s446s447 | 0.700 | 0.006 | 0.690 |
| CD44 | rs10128562rs713330rs7105890 | s446s447s448 | 0.526 | 0.823 | 1.000 |
| CD44 | rs10128562rs713330rs7105890rs2295756 | s446s447s448s449 | 0.526 | 0.395 | 0.510 |
| CD44 | rs713330rs7105890 | s447s448 | 0.163 | 0.936 | 0.799 |
| CD44 | rs713330rs7105890rs2295756 | s447s448s449 | 0.425 | 0.639 | 0.123 |
| CD44 | rs713330rs7105890rs2295756rs7116739 | s447s448s449s450 | 0.602 | 0.719 | 0.163 |
| CD44 | rs7105890rs2295756 | s448s449 | 0.893 | 0.959 | 0.981 |
| CD44 | rs7105890rs2295756rs7116739 | s448s449s450 | 0.791 | 0.612 | 0.735 |
| CD44 | rs7105890rs2295756rs7116739rs10128586 | s448s449s450s451 | 0.843 | 0.001 | 0.352 |
| CD44 | rs2295756rs7116739 | s449s450 | 0.625 | 0.631 | 0.328 |
| CD44 | rs2295756rs7116739rs10128586 | s449s450s451 | 0.726 | 0.474 | 0.580 |
| CD44 | rs2295756rs7116739rs10128586rs12419062 | s449s450s451s452 | 0.957 | 0.084 | 0.992 |
| CD44 | rs7116739rs10128586 | s450s451 | 0.567 | 0.141 | 0.101 |
| CD44 | rs7116739rs10128586rs12419062 | s450s451s452 | 0.840 | 0.936 | 0.836 |
| CD44 | rs10128586rs12419062 | s451s452 | 0.763 | 0.409 | 0.504 |
| CD5 | rs3862667rs616340 | s477s478 | 0.463 | 0.007 | 0.439 |
| CD5 | rs3862667rs616340rs4245224 | s477s478s479 | 0.518 | 0.018 | 0.523 |
| CD5 | rs3862667rs616340rs4245224rs7104333 | s477s478s479s480 | 0.686 | 0.001 | 0.449 |
| CD5 | rs616340rs4245224 | s478s479 | 0.447 | 0.159 | 0.457 |
| CD5 | rs616340rs4245224rs7104333 | s478s479s480 | 0.609 | 0.311 | 0.477 |
| CD5 | rs4245224rs7104333 | s479s480 | 0.279 | 0.209 | 0.327 |
| CD58 | rs10802189rs10802190 | s060s061 | 0.070 | 0.058 | 0.004 |
| CD58 | rs10802189rs10802190rs1414275 | s060s061s062 | 0.029 | 0.005 | 8.8E-05 |
| CD58 | rs10802189rs10802190rs1414275rs11588376 | s060s061s062s063 | 0.014 | 0.005 | 3.2E-05 |
| CD58 | rs10802190rs1414275 | s061s062 | 0.012 | 0.001 | 1.4E-05 |
| CD58 | rs10802190rs1414275rs11588376 | s061s062s063 | 0.010 | 0.001 | 1.2E-05 |
| CD58 | rs10802190rs1414275rs11588376rs1016140 | s061s062s063s064 | 0.013 | 0.003 | 2.4E-05 |
| CD58 | rs1414275rs11588376 | s062s063 | 0.008 | 4.7E-04 | 9.8E-06 |
| CD58 | rs1414275rs11588376rs1016140 | s062s063s064 | 0.010 | 0.001 | 6.8E-06 |
| CD58 | rs1414275rs11588376rs1016140rs1335532 | s062s063s064s065 | 0.024 | 0.006 | 3.9E-05 |
| CD58 | rs11588376rs1016140 | s063s064 | 0.009 | 0.001 | 6.9E-06 |
| CD58 | rs11588376rs1016140rs1335532 | s063s064s065 | 0.024 | 0.006 | 3.6E-05 |
| CD58 | rs11588376rs1016140rs1335532rs2300747 | s063s064s065s066 | 0.042 | 0.006 | 4.0E-05 |
| CD58 | rs1016140rs1335532 | s064s065 | 0.020 | 0.006 | 1.1E-04 |
| CD58 | rs1016140rs1335532rs2300747 | s064s065s066 | 0.042 | 0.005 | 6.6E-05 |
| CD58 | rs1016140rs1335532rs2300747rs10923122 | s064s065s066s067 | 0.006 | 0.042 | 2.7E-04 |
| CD58 | rs1335532rs2300747 | s065s066 | 0.065 | 0.006 | 3.4E-04 |
| CD58 | rs1335532rs2300747rs10923122 | s065s066s067 | 0.041 | 0.031 | 0.011 |
| CD58 | rs1335532rs2300747rs10923122rs7542681 | s065s066s067s068 | 0.257 | 0.029 | 0.007 |
| CD58 | rs2300747rs10923122 | s066s067 | 0.369 | 0.194 | 0.156 |
| CD58 | rs2300747rs10923122rs7542681 | s066s067s068 | 0.776 | 0.093 | 0.055 |
| CD58 | rs10923122rs7542681 | s067s068 | 0.014 | 0.381 | 0.022 |
| CD69 | rs1051065rs6416258 | s507s508 | 0.013 | 0.272 | 0.173 |
| CD69 | rs1051065rs6416258rs3176789 | s507s508s509 | 0.045 | 0.002 | 0.088 |
| CD69 | rs6416258rs3176789 | s508s509 | 0.628 | 0.468 | 0.622 |
| CD80 | rs7628626rs9855093 | s220s221 | 0.933 | 0.671 | 0.613 |
| CD80 | rs7628626rs9855093rs4688014 | s220s221s222 | 0.937 | 0.494 | 0.471 |
| CD80 | rs7628626rs9855093rs4688014rs624035 | s220s221s222s223 | 0.820 | 0.934 | 0.538 |
| CD80 | rs9855093rs4688014 | s221s222 | 0.693 | 0.417 | 0.363 |
| CD80 | rs9855093rs4688014rs624035 | s221s222s223 | 0.600 | 0.904 | 0.722 |
| CD80 | rs9855093rs4688014rs624035rs6804441 | s221s222s223s224 | 0.714 | 0.133 | 0.567 |
| CD80 | rs4688014rs624035 | s222s223 | 0.608 | 0.967 | 0.760 |
| CD80 | rs4688014rs624035rs6804441 | s222s223s224 | 0.711 | 0.113 | 0.592 |
| CD80 | rs4688014rs624035rs6804441rs13071247 | s222s223s224s225 | 0.397 | 0.154 | 0.464 |
| CD80 | rs624035rs6804441 | s223s224 | 0.705 | 0.217 | 0.421 |
| CD80 | rs624035rs6804441rs13071247 | s223s224s225 | 0.859 | 0.227 | 0.496 |
| CD80 | rs624035rs6804441rs13071247rs1485332 | s223s224s225s226 | 0.483 | 0.063 | 0.417 |
| CD80 | rs6804441rs13071247 | s224s225 | 0.993 | 0.157 | 0.462 |
| CD80 | rs6804441rs13071247rs1485332 | s224s225s226 | 0.361 | 0.141 | 0.282 |
| CD80 | rs6804441rs13071247rs1485332rs1880661 | s224s225s226s227 | 0.183 | 0.552 | 0.040 |
| CD80 | rs13071247rs1485332 | s225s226 | 0.229 | 0.635 | 0.782 |
| CD80 | rs13071247rs1485332rs1880661 | s225s226s227 | 0.047 | 0.315 | 0.722 |
| CD80 | rs1485332rs1880661 | s226s227 | 0.036 | 0.059 | 0.750 |
| CD86 | rs2681404rs6786977 | s228s229 | 0.110 | 0.359 | 0.317 |
| CD86 | rs2681404rs6786977rs2681411 | s228s229s230 | 0.246 | 0.437 | 0.003 |
| CD86 | rs2681404rs6786977rs2681411rs2681422 | s228s229s230s231 | 0.264 | 0.486 | 0.643 |
| CD86 | rs6786977rs2681411 | s229s230 | 0.138 | 0.459 | 0.292 |
| CD86 | rs6786977rs2681411rs2681422 | s229s230s231 | 0.264 | 0.514 | 0.435 |
| CD86 | rs6786977rs2681411rs2681422rs11717893 | s229s230s231s232 | 0.253 | 0.931 | 0.607 |
| CD86 | rs2681411rs2681422 | s230s231 | 0.234 | 0.864 | 0.303 |
| CD86 | rs2681411rs2681422rs11717893 | s230s231s232 | 0.354 | 0.950 | 0.448 |
| CD86 | rs2681411rs2681422rs11717893rs2332096 | s230s231s232s233 | 0.051 | 0.989 | 0.097 |
| CD86 | rs2681422rs11717893 | s231s232 | 0.374 | 0.903 | 0.465 |
| CD86 | rs2681422rs11717893rs2332096 | s231s232s233 | 0.009 | 0.939 | 0.082 |
| CD86 | rs2681422rs11717893rs2332096rs1915092 | s231s232s233s234 | 0.116 | 0.873 | 0.352 |
| CD86 | rs11717893rs2332096 | s232s233 | 0.026 | 0.856 | 0.066 |
| CD86 | rs11717893rs2332096rs1915092 | s232s233s234 | 0.075 | 0.534 | 0.073 |
| CD86 | rs11717893rs2332096rs1915092rs9848900 | s232s233s234s235 | 0.310 | 0.012 | 0.743 |
| CD86 | rs2332096rs1915092 | s233s234 | 0.047 | 0.770 | 0.127 |
| CD86 | rs2332096rs1915092rs9848900 | s233s234s235 | 0.187 | 0.567 | 0.276 |
| CD86 | rs2332096rs1915092rs9848900rs10804556 | s233s234s235s236 | 0.203 | 0.420 | 0.115 |
| CD86 | rs1915092rs9848900 | s234s235 | 0.377 | 0.674 | 0.318 |
| CD86 | rs1915092rs9848900rs10804556 | s234s235s236 | 0.203 | 0.280 | 0.064 |
| CD86 | rs1915092rs9848900rs10804556rs1129055 | s234s235s236s237 | 0.205 | 9.9E-05 | 0.062 |
| CD86 | rs9848900rs10804556 | s235s236 | 0.200 | 0.081 | 0.019 |
| CD86 | rs9848900rs10804556rs1129055 | s235s236s237 | 0.195 | 5.7E-05 | 0.023 |
| CD86 | rs10804556rs1129055 | s236s237 | 0.314 | 2.5E-04 | 5.0E-05 |
| CDC42 | rs2143104rs2473322 | s019s020 | 0.037 | 1.000 | 0.014 |
| CDC42 | rs2143104rs2473322rs2473317 | s019s020s021 | 0.151 | 0.317 | 0.042 |
| CDC42 | rs2143104rs2473322rs2473317rs2056974 | s019s020s021s022 | 0.067 | 0.207 | 0.038 |
| CDC42 | rs2473322rs2473317 | s020s021 | 0.035 | 0.317 | 0.043 |
| CDC42 | rs2473322rs2473317rs2056974 | s020s021s022 | 0.067 | 0.207 | 0.038 |
| CDC42 | rs2473322rs2473317rs2056974rs2473316 | s020s021s022s023 | 0.077 | 0.146 | 0.023 |
| CDC42 | rs2473317rs2056974 | s021s022 | 0.069 | 0.186 | 0.051 |
| CDC42 | rs2473317rs2056974rs2473316 | s021s022s023 | 0.088 | 0.049 | 0.005 |
| CDC42 | rs2473317rs2056974rs2473316rs10917148 | s021s022s023s024 | 0.077 | 0.146 | 0.023 |
| CDC42 | rs2056974rs2473316 | s022s023 | 0.030 | 0.072 | 0.003 |
| CDC42 | rs2056974rs2473316rs10917148 | s022s023s024 | 0.054 | 0.087 | 0.005 |
| CDC42 | rs2056974rs2473316rs10917148rs7519109 | s022s023s024s025 | 0.054 | 0.087 | 0.005 |
| CDC42 | rs2473316rs10917148 | s023s024 | 0.077 | 0.455 | 0.057 |
| CDC42 | rs2473316rs10917148rs7519109 | s023s024s025 | 0.372 | 0.455 | 0.057 |
| CDC42 | rs10917148rs7519109 | s024s025 | 0.037 | 1.000 | 0.014 |
| CIITA | rs4781016rs4774 | s572s573 | 0.112 | 0.632 | 0.653 |
| CIITA | rs4781016rs4774rs4781019 | s572s573s574 | 0.040 | 1.000 | 0.351 |
| CIITA | rs4781016rs4774rs4781019rs6498130 | s572s573s574s575 | 0.209 | 0.871 | 0.325 |
| CIITA | rs4774rs4781019 | s573s574 | 0.561 | 0.525 | 0.411 |
| CIITA | rs4774rs4781019rs6498130 | s573s574s575 | 0.558 | 0.970 | 0.691 |
| CIITA | rs4774rs4781019rs6498130rs4781024 | s573s574s575s576 | 0.546 | 0.134 | 0.121 |
| CIITA | rs4781019rs6498130 | s574s575 | 0.262 | 0.678 | 0.288 |
| CIITA | rs4781019rs6498130rs4781024 | s574s575s576 | 0.260 | 0.399 | 0.152 |
| CIITA | rs6498130rs4781024 | s575s576 | 0.115 | 0.532 | 0.102 |
| CISH | rs622502rs2239751 | s218s219 | 0.752 | 0.508 | 0.741 |
| CLEC7A | rs11053597rs11053603 | s510s511 | 0.448 | 0.264 | 0.291 |
| CLEC7A | rs11053597rs11053603rs2078178 | s510s511s512 | 0.549 | 0.277 | 0.313 |
| CLEC7A | rs11053603rs2078178 | s511s512 | 0.882 | 0.135 | 0.231 |
| CR2 | rs1567190rs1507764 | s123s124 | 0.700 | 0.187 | 0.188 |
| CR2 | rs1567190rs1507764rs1048971 | s123s124s125 | 0.870 | 0.305 | 0.326 |
| CR2 | rs1567190rs1507764rs1048971rs17615 | s123s124s125s126 | 0.879 | 0.314 | 0.426 |
| CR2 | rs1507764rs1048971 | s124s125 | 0.701 | 0.235 | 0.212 |
| CR2 | rs1507764rs1048971rs17615 | s124s125s126 | 0.756 | 0.292 | 0.332 |
| CR2 | rs1507764rs1048971rs17615rs2182912 | s124s125s126s127 | 0.565 | 0.046 | 0.279 |
| CR2 | rs1048971rs17615 | s125s126 | 0.580 | 0.747 | 0.385 |
| CR2 | rs1048971rs17615rs2182912 | s125s126s127 | 0.677 | 0.161 | 0.100 |
| CR2 | rs1048971rs17615rs2182912rs9429940 | s125s126s127s128 | 0.780 | 0.191 | 0.157 |
| CR2 | rs17615rs2182912 | s126s127 | 0.417 | 0.135 | 0.056 |
| CR2 | rs17615rs2182912rs9429940 | s126s127s128 | 0.501 | 0.003 | 0.058 |
| CR2 | rs2182912rs9429940 | s127s128 | 0.115 | 0.098 | 0.201 |
| CTLA4 | rs231777rs231779 | s202s203 | 0.832 | 0.510 | 0.529 |
| CTLA4 | rs231777rs231779rs3087243 | s202s203s204 | 0.962 | 0.527 | 0.690 |
| CTLA4 | rs231779rs3087243 | s203s204 | 0.865 | 0.337 | 0.533 |
| DEXI | rs3087519rs4072864 | s577s578 | 0.463 | 0.152 | 0.645 |
| DEXI | rs3087519rs4072864rs4072865 | s577s578s579 | 0.533 | 0.111 | 0.602 |
| DEXI | rs4072864rs4072865 | s578s579 | 0.273 | 0.283 | 0.964 |
| FAS | rs1324551rs6586165 | s425s426 | 0.383 | 1.000 | 0.370 |
| FAS | rs1324551rs6586165rs2147420 | s425s426s427 | 0.380 | 0.003 | 0.435 |
| FAS | rs1324551rs6586165rs2147420rs3218619 | s425s426s427s428 | 0.591 | 0.439 | 0.277 |
| FAS | rs6586165rs2147420 | s426s427 | 0.438 | 0.003 | 0.435 |
| FAS | rs6586165rs2147420rs3218619 | s426s427s428 | 0.652 | 0.897 | 0.277 |
| FAS | rs6586165rs2147420rs3218619rs3218614 | s426s427s428s429 | 0.776 | 0.266 | 0.343 |
| FAS | rs2147420rs3218619 | s427s428 | 0.506 | 0.482 | 0.293 |
| FAS | rs2147420rs3218619rs3218614 | s427s428s429 | 0.349 | 0.162 | 0.278 |
| FAS | rs2147420rs3218619rs3218614rs9658767 | s427s428s429s430 | 0.748 | 0.328 | 0.471 |
| FAS | rs3218619rs3218614 | s428s429 | 0.701 | 0.817 | 0.906 |
| FAS | rs3218619rs3218614rs9658767 | s428s429s430 | 0.402 | 1.000 | 0.194 |
| FAS | rs3218614rs9658767 | s429s430 | 0.704 | 0.410 | 0.486 |
| FCGR2C | rs6427597rs410236 | s073s074 | 0.704 | 0.506 | 0.301 |
| GATA3 | rs1399180rs3802604 | s414s415 | 0.187 | 0.673 | 0.351 |
| GATA3 | rs1399180rs3802604rs570613 | s414s415s416 | 0.323 | 0.230 | 0.511 |
| GATA3 | rs1399180rs3802604rs570613rs3802600 | s414s415s416s417 | 0.070 | 0.960 | 0.306 |
| GATA3 | rs3802604rs570613 | s415s416 | 0.396 | 0.029 | 0.484 |
| GATA3 | rs3802604rs570613rs3802600 | s415s416s417 | 0.515 | 0.730 | 0.125 |
| GATA3 | rs3802604rs570613rs3802600rs528778 | s415s416s417s418 | 0.290 | 0.060 | 0.239 |
| GATA3 | rs570613rs3802600 | s416s417 | 0.654 | 2.0E-06 | 0.602 |
| GATA3 | rs570613rs3802600rs528778 | s416s417s418 | 0.253 | 5.9E-06 | 0.639 |
| GATA3 | rs3802600rs528778 | s417s418 | 0.637 | 0.249 | 0.173 |
| GNB3 | rs5441rs5446 | s494s498 | 0.157 | 0.035 | 0.007 |
| GPNMB | rs199357rs1881203 | s347s348 | 0.529 | 0.713 | 0.793 |
| GPNMB | rs199357rs1881203rs858275 | s347s348s349 | 0.906 | 0.390 | 0.947 |
| GPNMB | rs199357rs1881203rs858275rs5850 | s347s348s349s350 | 0.526 | 0.248 | 0.605 |
| GPNMB | rs1881203rs858275 | s348s349 | 0.656 | 0.380 | 0.861 |
| GPNMB | rs1881203rs858275rs5850 | s348s349s350 | 0.334 | 0.706 | 0.852 |
| GPNMB | rs858275rs5850 | s349s350 | 0.189 | 0.010 | 0.468 |
| ICOS | rs4675374rs4389322 | s205s206 | 0.158 | 0.595 | 0.127 |
| ICOS | rs4675374rs4389322rs4521021 | s205s206s207 | 0.224 | 0.613 | 0.151 |
| ICOS | rs4675374rs4389322rs4521021rs11571314 | s205s206s207s208 | 0.092 | 0.781 | 0.180 |
| ICOS | rs4389322rs4521021 | s206s207 | 0.107 | 0.728 | 0.243 |
| ICOS | rs4389322rs4521021rs11571314 | s206s207s208 | 0.075 | 0.998 | 0.431 |
| ICOS | rs4389322rs4521021rs11571314rs10172036 | s206s207s208s209 | 0.063 | 0.077 | 0.167 |
| ICOS | rs4521021rs11571314 | s207s208 | 0.155 | 0.714 | 0.194 |
| ICOS | rs4521021rs11571314rs10172036 | s207s208s209 | 0.166 | 0.487 | 0.071 |
| ICOS | rs4521021rs11571314rs10172036rs4404254 | s207s208s209s210 | 0.142 | 0.503 | 0.052 |
| ICOS | rs11571314rs10172036 | s208s209 | 0.053 | 0.380 | 0.111 |
| ICOS | rs11571314rs10172036rs4404254 | s208s209s210 | 0.053 | 0.398 | 0.129 |
| ICOS | rs10172036rs4404254 | s209s210 | 0.050 | 0.450 | 0.117 |
| ICOSLG | rs4819388rs2070558 | s705s706 | 0.857 | 0.201 | 0.312 |
| ICOSLG | rs4819388rs2070558rs2070561 | s705s706s707 | 0.179 | 0.117 | 0.128 |
| ICOSLG | rs2070558rs2070561 | s706s707 | 0.691 | 0.458 | 0.379 |
| IFNAR1 | rs2856968rs2243590 | s686s687 | 0.177 | 0.954 | 0.372 |
| IFNAR1 | rs2856968rs2243590rs2257167 | s686s687s688 | 0.403 | 1.000 | 0.417 |
| IFNAR1 | rs2856968rs2243590rs2257167rs17875834 | s686s687s688s690 | 0.353 | 0.146 | 0.029 |
| IFNAR1 | rs2243590rs2257167 | s687s688 | 0.403 | 4.2E-04 | 0.418 |
| IFNAR1 | rs2243590rs2257167rs17875834 | s687s688s690 | 0.353 | 0.133 | 0.031 |
| IFNAR1 | rs2243590rs2257167rs17875834rs2834202 | s687s688s690s691 | 0.075 | 0.022 | 0.064 |
| IFNAR1 | rs2257167rs17875834 | s688s690 | 0.189 | 0.459 | 0.087 |
| IFNAR1 | rs2257167rs17875834rs2834202 | s688s690s691 | 0.315 | 0.060 | 0.057 |
| IFNAR1 | rs17875834rs2834202 | s690s691 | 0.199 | 0.069 | 0.047 |
| IFNAR2 | rs11088247rs2248202 | s672s673 | 0.372 | 0.613 | 0.682 |
| IFNAR2 | rs11088247rs2248202rs7279064 | s672s673s674 | 0.996 | 0.608 | 0.702 |
| IFNAR2 | rs11088247rs2248202rs7279064rs2834158 | s672s673s674s675 | 0.996 | 0.651 | 0.762 |
| IFNAR2 | rs2248202rs7279064 | s673s674 | 0.417 | 0.635 | 0.395 |
| IFNAR2 | rs2248202rs7279064rs2834158 | s673s674s675 | 0.996 | 0.628 | 0.708 |
| IFNAR2 | rs2248202rs7279064rs2834158rs11911133 | s673s674s675s677 | 0.957 | 0.478 | 0.414 |
| IFNAR2 | rs7279064rs2834158 | s674s675 | 0.766 | 0.942 | 0.848 |
| IFNAR2 | rs7279064rs2834158rs11911133 | s674s675s677 | 0.959 | 0.012 | 0.829 |
| IFNAR2 | rs7279064rs2834158rs11911133rs2250226 | s674s675s677s678 | 0.304 | 0.019 | 0.270 |
| IFNAR2 | rs2834158rs11911133 | s675s677 | 0.917 | 0.003 | 0.619 |
| IFNAR2 | rs2834158rs11911133rs2250226 | s675s677s678 | 0.289 | 0.019 | 0.509 |
| IFNAR2 | rs11911133rs2250226 | s677s678 | 0.158 | 0.019 | 0.600 |
| IFNG | rs2069727rs2069718 | s523s524 | 0.121 | 0.375 | 0.056 |
| IFNGR1 | rs9376267rs9376268 | s313s314 | 0.015 | 0.572 | 0.346 |
| IFNGR2 | rs2284553rs9808685 | s692s693 | 0.042 | 0.129 | 0.038 |
| IFNGR2 | rs2284553rs9808685rs2834210 | s692s693s694 | 0.084 | 0.235 | 0.066 |
| IFNGR2 | rs2284553rs9808685rs2834210rs9808753 | s692s693s694s695 | 0.091 | 1.000 | 0.001 |
| IFNGR2 | rs9808685rs2834210 | s693s694 | 0.100 | 0.265 | 0.067 |
| IFNGR2 | rs9808685rs2834210rs9808753 | s693s694s695 | 0.074 | 0.525 | 0.065 |
| IFNGR2 | rs9808685rs2834210rs9808753rs2834214 | s693s694s695s696 | 0.084 | 0.472 | 0.070 |
| IFNGR2 | rs2834210rs9808753 | s694s695 | 0.305 | 0.825 | 0.731 |
| IFNGR2 | rs2834210rs9808753rs2834214 | s694s695s696 | 0.323 | 0.371 | 0.783 |
| IFNGR2 | rs2834210rs9808753rs2834214rs2834215 | s694s695s696s697 | 0.323 | 0.001 | 0.783 |
| IFNGR2 | rs9808753rs2834214 | s695s696 | 0.347 | 0.725 | 0.854 |
| IFNGR2 | rs9808753rs2834214rs2834215 | s695s696s697 | 0.347 | 0.725 | 0.854 |
| IFNGR2 | rs2834214rs2834215 | s696s697 | 0.180 | 0.465 | 0.594 |
| IL10 | rs3024498il10_592 | s112s113 | 0.727 | 0.279 | 0.215 |
| IL10 | rs3024498il10_592il10_1082 | s112s113s114 | 0.120 | 0.260 | 0.189 |
| IL10 | il10_592il10_1082 | s113s114 | 0.144 | 0.149 | 0.097 |
| IL10RA | rs2508450rs4252279 | s481s482 | 0.131 | 0.042 | 0.007 |
| IL10RA | rs2508450rs4252279rs2229113 | s481s482s483 | 0.289 | 0.052 | 0.013 |
| IL10RA | rs2508450rs4252279rs2229113rs9610 | s481s482s483s484 | 0.392 | 1.000 | 0.025 |
| IL10RA | rs4252279rs2229113 | s482s483 | 0.492 | 0.361 | 0.190 |
| IL10RA | rs4252279rs2229113rs9610 | s482s483s484 | 0.611 | 0.011 | 0.173 |
| IL10RA | rs2229113rs9610 | s483s484 | 0.609 | 0.413 | 0.251 |
| IL10RB | rs2834167rs2284552 | s679s680 | 0.326 | 0.289 | 0.685 |
| IL10RB | rs2834167rs2284552rs962859 | s679s680s681 | 0.410 | 0.397 | 0.913 |
| IL10RB | rs2834167rs2284552rs962859rs2243498 | s679s680s681s682 | 0.521 | 0.020 | 0.219 |
| IL10RB | rs2284552rs962859 | s680s681 | 0.342 | 0.311 | 0.880 |
| IL10RB | rs2284552rs962859rs2243498 | s680s681s682 | 0.500 | 0.313 | 0.942 |
| IL10RB | rs2284552rs962859rs2243498rs2247526 | s680s681s682s683 | 0.244 | 0.259 | 0.380 |
| IL10RB | rs962859rs2243498 | s681s682 | 0.664 | 0.239 | 0.642 |
| IL10RB | rs962859rs2243498rs2247526 | s681s682s683 | 0.317 | 0.391 | 0.022 |
| IL10RB | rs962859rs2243498rs2247526rs2834174 | s681s682s683s684 | 0.058 | 0.239 | 0.010 |
| IL10RB | rs2243498rs2247526 | s682s683 | 0.195 | 0.296 | 0.169 |
| IL10RB | rs2243498rs2247526rs2834174 | s682s683s684 | 0.248 | 0.530 | 0.277 |
| IL10RB | rs2243498rs2247526rs2834174rs3171425 | s682s683s684s685 | 0.411 | 0.365 | 0.458 |
| IL10RB | rs2247526rs2834174 | s683s684 | 0.118 | 0.268 | 0.102 |
| IL10RB | rs2247526rs2834174rs3171425 | s683s684s685 | 0.192 | 0.362 | 0.238 |
| IL10RB | rs2834174rs3171425 | s684s685 | 0.225 | 0.476 | 0.281 |
| IL12RB1 | rs375947rs429774 | s650s651 | 0.706 | 0.767 | 0.616 |
| IL12RB1 | rs375947rs429774rs376008 | s650s651s652 | 0.200 | 0.007 | 0.049 |
| IL12RB1 | rs429774rs376008 | s651s652 | 0.614 | 0.003 | 0.165 |
| IL12RB2 | rs11209046rs3790558 | s045s046 | 0.234 | 0.182 | 0.718 |
| IL12RB2 | rs11209046rs3790558rs2066446 | s045s046s047 | 0.695 | 0.243 | 0.745 |
| IL12RB2 | rs11209046rs3790558rs2066446rs2066445 | s045s046s047s048 | 0.731 | 0.199 | 0.682 |
| IL12RB2 | rs3790558rs2066446 | s046s047 | 0.309 | 0.200 | 0.600 |
| IL12RB2 | rs3790558rs2066446rs2066445 | s046s047s048 | 0.172 | 0.376 | 0.633 |
| IL12RB2 | rs3790558rs2066446rs2066445rs3790566 | s046s047s048s049 | 0.499 | 0.169 | 0.775 |
| IL12RB2 | rs2066446rs2066445 | s047s048 | 0.810 | 0.130 | 0.897 |
| IL12RB2 | rs2066446rs2066445rs3790566 | s047s048s049 | 0.714 | 0.071 | 0.407 |
| IL12RB2 | rs2066446rs2066445rs3790566rs3790567 | s047s048s049s050 | 0.714 | 0.360 | 0.403 |
| IL12RB2 | rs2066445rs3790566 | s048s049 | 0.832 | 0.117 | 0.709 |
| IL12RB2 | rs2066445rs3790566rs3790567 | s048s049s050 | 0.832 | 0.116 | 0.706 |
| IL12RB2 | rs2066445rs3790566rs3790567rs12564159 | s048s049s050s051 | 0.142 | 0.189 | 0.204 |
| IL12RB2 | rs3790566rs3790567 | s049s050 | 0.993 | 0.053 | 0.330 |
| IL12RB2 | rs3790566rs3790567rs12564159 | s049s050s051 | 0.038 | 0.375 | 0.049 |
| IL12RB2 | rs3790566rs3790567rs12564159rs2307145 | s049s050s051s052 | 0.114 | 0.646 | 0.147 |
| IL12RB2 | rs3790567rs12564159 | s050s051 | 0.038 | 0.375 | 0.049 |
| IL12RB2 | rs3790567rs12564159rs2307145 | s050s051s052 | 0.114 | 0.646 | 0.147 |
| IL12RB2 | rs3790567rs12564159rs2307145rs881087 | s050s051s052s053 | 0.114 | 0.637 | 0.134 |
| IL12RB2 | rs12564159rs2307145 | s051s052 | 0.271 | 0.616 | 0.366 |
| IL12RB2 | rs12564159rs2307145rs881087 | s051s052s053 | 0.271 | 0.638 | 0.357 |
| IL12RB2 | rs2307145rs881087 | s052s053 | 0.276 | 0.638 | 0.357 |
| IL15 | rs6837991rs990851 | s252s253 | 0.171 | 0.872 | 0.270 |
| IL15 | rs6837991rs990851rs1519551 | s252s253s254 | 0.274 | 1.000 | 0.499 |
| IL15 | rs6837991rs990851rs1519551rs1519552 | s252s253s254s255 | 0.302 | 0.998 | 0.488 |
| IL15 | rs990851rs1519551 | s253s254 | 0.143 | 0.986 | 0.272 |
| IL15 | rs990851rs1519551rs1519552 | s253s254s255 | 0.161 | 0.986 | 0.299 |
| IL15 | rs990851rs1519551rs1519552rs7688250 | s253s254s255s256 | 0.230 | 0.669 | 0.371 |
| IL15 | rs1519551rs1519552 | s254s255 | 0.185 | 0.994 | 0.362 |
| IL15 | rs1519551rs1519552rs7688250 | s254s255s256 | 0.263 | 0.635 | 0.412 |
| IL15 | rs1519551rs1519552rs7688250rs10028670 | s254s255s256s257 | 0.137 | 0.518 | 0.263 |
| IL15 | rs1519552rs7688250 | s255s256 | 0.204 | 0.385 | 0.249 |
| IL15 | rs1519552rs7688250rs10028670 | s255s256s257 | 0.061 | 0.288 | 0.179 |
| IL15 | rs1519552rs7688250rs10028670rs1040257 | s255s256s257s258 | 0.061 | 0.288 | 0.120 |
| IL15 | rs7688250rs10028670 | s256s257 | 1.000 | 1.000 | 0.120 |
| IL15 | rs7688250rs10028670rs1040257 | s256s257s258 | 1.000 | 1.000 | 0.063 |
| IL15 | rs7688250rs10028670rs1040257rs6841454 | s256s257s258s259 | 1.000 | 1.000 | 0.063 |
| IL15 | rs10028670rs1040257 | s257s258 | 1.000 | 1.000 | 0.063 |
| IL15 | rs10028670rs1040257rs6841454 | s257s258s259 | 1.000 | 1.000 | 0.063 |
| IL15 | rs10028670rs1040257rs6841454rs1519553 | s257s258s259s260 | 1.000 | 1.000 | 0.063 |
| IL15 | rs1040257rs6841454 | s258s259 | 1.000 | 1.000 | 0.063 |
| IL15 | rs1040257rs6841454rs1519553 | s258s259s260 | 1.000 | 1.000 | 0.063 |
| IL15 | rs1040257rs6841454rs1519553rs1907949 | s258s259s260s261 | 1.000 | 0.074 | 0.065 |
| IL15 | rs6841454rs1519553 | s259s260 | 1.000 | 1.000 | 1.000 |
| IL15 | rs6841454rs1519553rs1907949 | s259s260s261 | 0.512 | 0.811 | 0.770 |
| IL15 | rs6841454rs1519553rs1907949rs12508955 | s259s260s261s262 | 1.000 | 1.000 | 0.072 |
| IL15 | rs1519553rs1907949 | s260s261 | 0.512 | 0.811 | 0.770 |
| IL15 | rs1519553rs1907949rs12508955 | s260s261s262 | 1.000 | 1.000 | 0.072 |
| IL15 | rs1907949rs12508955 | s261s262 | 1.000 | 1.000 | 0.096 |
| IL15RA | rs2296135rs8177726 | s408s409 | 0.830 | 0.620 | 0.595 |
| IL15RA | rs2296135rs8177726rs7097780 | s408s409s411 | 0.245 | 0.499 | 0.166 |
| IL15RA | rs2296135rs8177726rs7097780rs8177772 | s408s409s411s412 | 0.179 | 0.447 | 0.112 |
| IL15RA | rs8177726rs7097780 | s409s411 | 0.081 | 0.129 | 0.005 |
| IL15RA | rs8177726rs7097780rs8177772 | s409s411s412 | 0.051 | 0.785 | 0.010 |
| IL15RA | rs8177726rs7097780rs8177772rs8177633 | s409s411s412s413 | 0.074 | 0.312 | 0.022 |
| IL15RA | rs7097780rs8177772 | s411s412 | 0.273 | 0.446 | 0.234 |
| IL15RA | rs7097780rs8177772rs8177633 | s411s412s413 | 0.121 | 0.574 | 1.000 |
| IL15RA | rs8177772rs8177633 | s412s413 | 0.033 | 0.515 | 0.674 |
| IL19 | rs4347211rs3950619 | s115s116 | 0.136 | 0.267 | 0.033 |
| IL19 | rs4347211rs3950619rs1878673 | s115s116s117 | 0.171 | 0.209 | 0.032 |
| IL19 | rs4347211rs3950619rs1878673rs12409415 | s115s116s117s118 | 0.207 | 0.058 | 0.024 |
| IL19 | rs3950619rs1878673 | s116s117 | 0.078 | 0.047 | 0.005 |
| IL19 | rs3950619rs1878673rs12409415 | s116s117s118 | 0.074 | 0.017 | 0.003 |
| IL19 | rs3950619rs1878673rs12409415rs2056225 | s116s117s118s119 | 0.049 | 0.026 | 0.005 |
| IL19 | rs1878673rs12409415 | s117s118 | 0.598 | 0.005 | 0.085 |
| IL19 | rs1878673rs12409415rs2056225 | s117s118s119 | 0.197 | 0.029 | 0.067 |
| IL19 | rs1878673rs12409415rs2056225rs2243158 | s117s118s119s120 | 0.123 | 0.020 | 0.049 |
| IL19 | rs12409415rs2056225 | s118s119 | 0.147 | 0.094 | 0.028 |
| IL19 | rs12409415rs2056225rs2243158 | s118s119s120 | 0.146 | 0.023 | 8.7E-04 |
| IL19 | rs12409415rs2056225rs2243158rs2243174 | s118s119s120s121 | 0.228 | 0.027 | 0.005 |
| IL19 | rs2056225rs2243158 | s119s120 | 0.067 | 2.3E-04 | 0.042 |
| IL19 | rs2056225rs2243158rs2243174 | s119s120s121 | 0.187 | 0.199 | 0.080 |
| IL19 | rs2056225rs2243158rs2243174rs2243191 | s119s120s121s122 | 0.020 | 0.004 | 0.005 |
| IL19 | rs2243158rs2243174 | s120s121 | 0.185 | 0.166 | 0.069 |
| IL19 | rs2243158rs2243174rs2243191 | s120s121s122 | 0.286 | 0.157 | 0.060 |
| IL19 | rs2243174rs2243191 | s121s122 | 0.419 | 0.271 | 0.275 |
| IL1A | rs2856836rs17561 | s166s167 | 0.756 | 0.544 | 0.784 |
| IL1A | rs2856836rs17561rs1609682 | s166s167s168 | 0.617 | 0.746 | 0.959 |
| IL1A | rs2856836rs17561rs1609682rs1800587 | s166s167s168s169 | 0.720 | 0.649 | 1.000 |
| IL1A | rs17561rs1609682 | s167s168 | 0.714 | 0.746 | 0.959 |
| IL1A | rs17561rs1609682rs1800587 | s167s168s169 | 0.791 | 0.580 | 0.992 |
| IL1A | rs1609682rs1800587 | s168s169 | 0.796 | 0.612 | 0.974 |
| IL1B | rs1143643rs1143627 | s170s171 | 0.595 | 0.138 | 0.232 |
| IL1R1 | rs2287047rs997049 | s145s146 | 0.215 | 0.081 | 0.613 |
| IL1R1 | rs2287047rs997049rs3917299 | s145s146s147 | 0.001 | 0.047 | 0.005 |
| IL1R1 | rs2287047rs997049rs3917299rs3171845 | s145s146s147s148 | 0.007 | 0.073 | 0.206 |
| IL1R1 | rs997049rs3917299 | s146s147 | 0.018 | 0.358 | 9.4E-04 |
| IL1R1 | rs997049rs3917299rs3171845 | s146s147s148 | 0.098 | 0.280 | 0.093 |
| IL1R1 | rs997049rs3917299rs3171845rs3917332 | s146s147s148s149 | 0.135 | 0.293 | 0.003 |
| IL1R1 | rs3917299rs3171845 | s147s148 | 0.371 | 0.128 | 0.066 |
| IL1R1 | rs3917299rs3171845rs3917332 | s147s148s149 | 0.304 | 0.232 | 0.045 |
| IL1R1 | rs3171845rs3917332 | s148s149 | 0.259 | 0.143 | 0.031 |
| IL1R2 | rs4851521rs4851526 | s139s140 | 0.869 | 0.434 | 0.583 |
| IL1R2 | rs4851521rs4851526rs4851527 | s139s140s141 | 0.924 | 0.756 | 0.609 |
| IL1R2 | rs4851521rs4851526rs4851527rs719248 | s139s140s141s142 | 0.723 | 1.000 | 0.591 |
| IL1R2 | rs4851526rs4851527 | s140s141 | 0.650 | 0.552 | 0.637 |
| IL1R2 | rs4851526rs4851527rs719248 | s140s141s142 | 0.489 | 1.000 | 0.640 |
| IL1R2 | rs4851526rs4851527rs719248rs2072474 | s140s141s142s143 | 0.660 | 0.166 | 0.453 |
| IL1R2 | rs4851527rs719248 | s141s142 | 0.383 | 0.757 | 0.352 |
| IL1R2 | rs4851527rs719248rs2072474 | s141s142s143 | 0.540 | 0.397 | 0.666 |
| IL1R2 | rs4851527rs719248rs2072474rs3218979 | s141s142s143s144 | 0.470 | 0.617 | 0.003 |
| IL1R2 | rs719248rs2072474 | s142s143 | 0.569 | 0.963 | 0.655 |
| IL1R2 | rs719248rs2072474rs3218979 | s142s143s144 | 0.550 | 0.530 | 0.004 |
| IL1R2 | rs2072474rs3218979 | s143s144 | 0.393 | 0.742 | 0.081 |
| IL1RL1 | rs2310220rs1041973 | s160s161 | 0.837 | 0.677 | 0.801 |
| IL1RL1 | rs2310220rs1041973rs1420101 | s160s161s162 | 0.946 | 0.621 | 0.825 |
| IL1RL1 | rs2310220rs1041973rs1420101rs12989197 | s160s161s162s163 | 0.903 | 0.552 | 0.689 |
| IL1RL1 | rs1041973rs1420101 | s161s162 | 0.946 | 0.504 | 0.835 |
| IL1RL1 | rs1041973rs1420101rs12989197 | s161s162s163 | 0.921 | 0.431 | 0.581 |
| IL1RL1 | rs1041973rs1420101rs12989197rs4988956 | s161s162s163s164 | 0.286 | 0.358 | 0.078 |
| IL1RL1 | rs1420101rs12989197 | s162s163 | 0.850 | 0.259 | 0.552 |
| IL1RL1 | rs1420101rs12989197rs4988956 | s162s163s164 | 0.821 | 0.620 | 0.495 |
| IL1RL1 | rs1420101rs12989197rs4988956rs10192157 | s162s163s164s165 | 0.875 | 0.620 | 0.493 |
| IL1RL1 | rs12989197rs4988956 | s163s164 | 0.714 | 0.648 | 0.774 |
| IL1RL1 | rs12989197rs4988956rs10192157 | s163s164s165 | 0.799 | 0.648 | 0.769 |
| IL1RL1 | rs4988956rs10192157 | s164s165 | 0.716 | 0.702 | 0.577 |
| IL1RL2 | rs1997503rs1558648 | s150s151 | 0.893 | 0.822 | 0.787 |
| IL1RL2 | rs1997503rs1558648rs11123913 | s150s151s152 | 0.770 | 0.862 | 0.730 |
| IL1RL2 | rs1997503rs1558648rs11123913rs1922291 | s150s151s152s153 | 0.605 | 0.929 | 0.645 |
| IL1RL2 | rs1558648rs11123913 | s151s152 | 0.587 | 0.787 | 0.547 |
| IL1RL2 | rs1558648rs11123913rs1922291 | s151s152s153 | 0.456 | 0.969 | 0.606 |
| IL1RL2 | rs1558648rs11123913rs1922291rs917994 | s151s152s153s154 | 0.412 | 0.973 | 0.566 |
| IL1RL2 | rs11123913rs1922291 | s152s153 | 0.317 | 0.741 | 0.286 |
| IL1RL2 | rs11123913rs1922291rs917994 | s152s153s154 | 0.146 | 1.000 | 0.132 |
| IL1RL2 | rs11123913rs1922291rs917994rs2302621 | s152s153s154s155 | 0.256 | 1.000 | 0.264 |
| IL1RL2 | rs1922291rs917994 | s153s154 | 0.129 | 0.262 | 0.131 |
| IL1RL2 | rs1922291rs917994rs2302621 | s153s154s155 | 0.100 | 0.671 | 0.135 |
| IL1RL2 | rs1922291rs917994rs2302621rs3755285 | s153s154s155s156 | 0.262 | 0.542 | 0.248 |
| IL1RL2 | rs917994rs2302621 | s154s155 | 0.111 | 0.563 | 0.138 |
| IL1RL2 | rs917994rs2302621rs3755285 | s154s155s156 | 0.283 | 0.422 | 0.174 |
| IL1RL2 | rs917994rs2302621rs3755285rs955754 | s154s155s156s157 | 0.233 | 0.026 | 0.187 |
| IL1RL2 | rs2302621rs3755285 | s155s156 | 0.283 | 0.422 | 0.170 |
| IL1RL2 | rs2302621rs3755285rs955754 | s155s156s157 | 0.233 | 0.729 | 0.182 |
| IL1RL2 | rs2302621rs3755285rs955754rs6709635 | s155s156s157s158 | 0.286 | 0.766 | 0.047 |
| IL1RL2 | rs3755285rs955754 | s156s157 | 0.416 | 0.257 | 0.156 |
| IL1RL2 | rs3755285rs955754rs6709635 | s156s157s158 | 0.302 | 0.259 | 0.114 |
| IL1RL2 | rs3755285rs955754rs6709635rs2302612 | s156s157s158s159 | 0.302 | 0.259 | 0.120 |
| IL1RL2 | rs955754rs6709635 | s157s158 | 0.217 | 0.239 | 0.081 |
| IL1RL2 | rs955754rs6709635rs2302612 | s157s158s159 | 0.217 | 0.239 | 0.086 |
| IL1RL2 | rs6709635rs2302612 | s158s159 | 0.141 | 0.241 | 0.063 |
| IL22 | rs1182844rs1179251 | s530s531 | 0.965 | 0.071 | 0.992 |
| IL22 | rs1182844rs1179251rs2046068 | s530s531s532 | 0.921 | 1.000 | 0.961 |
| IL22 | rs1179251rs2046068 | s531s532 | 0.578 | 0.886 | 0.872 |
| IL22RA1 | rs3795300rs3795299 | s026s027 | 0.406 | 0.536 | 0.241 |
| IL22RA1 | rs3795300rs3795299rs10903022 | s026s027s028 | 0.868 | 1.000 | 0.584 |
| IL22RA1 | rs3795300rs3795299rs10903022rs4292900 | s026s027s028s029 | 0.810 | 0.293 | 0.417 |
| IL22RA1 | rs3795299rs10903022 | s027s028 | 0.767 | 0.943 | 0.747 |
| IL22RA1 | rs3795299rs10903022rs4292900 | s027s028s029 | 0.581 | 0.386 | 0.647 |
| IL22RA1 | rs10903022rs4292900 | s028s029 | 0.552 | 2.0E-09 | 0.768 |
| IL22RA2 | rs1543509rs202567 | s306s307 | 0.939 | 0.772 | 0.799 |
| IL22RA2 | rs1543509rs202567rs7774349 | s306s307s308 | 0.939 | 0.772 | 0.799 |
| IL22RA2 | rs1543509rs202567rs7774349rs11154914 | s306s307s308s309 | 0.345 | 0.972 | 0.736 |
| IL22RA2 | rs202567rs7774349 | s307s308 | 0.624 | 1.000 | 0.190 |
| IL22RA2 | rs202567rs7774349rs11154914 | s307s308s309 | 0.869 | 0.029 | 0.307 |
| IL22RA2 | rs202567rs7774349rs11154914rs10457018 | s307s308s309s310 | 0.837 | 4.1E-05 | 0.207 |
| IL22RA2 | rs7774349rs11154914 | s308s309 | 0.869 | 0.029 | 0.307 |
| IL22RA2 | rs7774349rs11154914rs10457018 | s308s309s310 | 0.837 | 0.038 | 0.207 |
| IL22RA2 | rs7774349rs11154914rs10457018rs9376263 | s308s309s310s311 | 0.925 | 1.000 | 0.216 |
| IL22RA2 | rs11154914rs10457018 | s309s310 | 0.761 | 0.011 | 1.000 |
| IL22RA2 | rs11154914rs10457018rs9376263 | s309s310s311 | 0.921 | 0.176 | 0.332 |
| IL22RA2 | rs11154914rs10457018rs9376263rs6570136 | s309s310s311s312 | 0.280 | 0.283 | 1.000 |
| IL22RA2 | rs10457018rs9376263 | s310s311 | 0.828 | 0.124 | 0.563 |
| IL22RA2 | rs10457018rs9376263rs6570136 | s310s311s312 | 0.468 | 0.195 | 0.538 |
| IL22RA2 | rs9376263rs6570136 | s311s312 | 0.777 | 0.194 | 0.603 |
| IL26 | rs10748100rs2870946 | s525s526 | 0.571 | 0.960 | 0.921 |
| IL26 | rs10748100rs2870946rs7132188 | s525s526s527 | 4.6E-04 | 3.3E-05 | 0.994 |
| IL26 | rs10748100rs2870946rs7132188rs3782554 | s525s526s527s528 | 0.102 | 0.344 | 0.659 |
| IL26 | rs2870946rs7132188 | s526s527 | 0.908 | 0.940 | 0.903 |
| IL26 | rs2870946rs7132188rs3782554 | s526s527s528 | 0.498 | 0.581 | 1.000 |
| IL26 | rs2870946rs7132188rs3782554rs3814240 | s526s527s528s529 | 0.297 | 0.773 | 0.529 |
| IL26 | rs7132188rs3782554 | s527s528 | 0.617 | 1.000 | 0.500 |
| IL26 | rs7132188rs3782554rs3814240 | s527s528s529 | 0.434 | 0.331 | 0.091 |
| IL26 | rs3782554rs3814240 | s528s529 | 0.417 | 0.864 | 0.340 |
| IL28RA | rs11249006rs7552086 | s030s031 | 0.570 | 0.915 | 0.710 |
| IL28RA | rs11249006rs7552086rs10903038 | s030s031s032 | 0.503 | 0.963 | 0.909 |
| IL28RA | rs11249006rs7552086rs10903038rs7520329 | s030s031s032s033 | 0.799 | 0.354 | 0.442 |
| IL28RA | rs7552086rs10903038 | s031s032 | 0.391 | 0.990 | 0.745 |
| IL28RA | rs7552086rs10903038rs7520329 | s031s032s033 | 0.548 | 0.453 | 0.420 |
| IL28RA | rs7552086rs10903038rs7520329rs3897440 | s031s032s033s034 | 0.730 | 0.235 | 0.285 |
| IL28RA | rs10903038rs7520329 | s032s033 | 0.546 | 0.952 | 0.664 |
| IL28RA | rs10903038rs7520329rs3897440 | s032s033s034 | 0.879 | 0.481 | 0.742 |
| IL28RA | rs10903038rs7520329rs3897440rs4489498 | s032s033s034s035 | 0.538 | 0.304 | 0.588 |
| IL28RA | rs7520329rs3897440 | s033s034 | 0.860 | 0.393 | 0.507 |
| IL28RA | rs7520329rs3897440rs4489498 | s033s034s035 | 0.717 | 0.004 | 0.537 |
| IL28RA | rs3897440rs4489498 | s034s035 | 0.919 | 0.597 | 0.824 |
| IL4 | rs2070874rs2243268 | s275s276 | 0.053 | 0.772 | 0.226 |
| IL4 | rs2070874rs2243268rs2243274 | s275s276s277 | 0.194 | 0.264 | 0.321 |
| IL4 | rs2243268rs2243274 | s276s277 | 0.345 | 0.117 | 0.603 |
| IL4R | rs4787948rs3024548 | s582s583 | 0.484 | 0.796 | 0.356 |
| IL4R | rs4787948rs3024548rs3024672 | s582s583s584 | 0.136 | 0.051 | 0.496 |
| IL4R | rs4787948rs3024548rs3024672rs1805011 | s582s583s584s585 | 0.437 | 0.977 | 0.715 |
| IL4R | rs3024548rs3024672 | s583s584 | 0.075 | 0.630 | 0.301 |
| IL4R | rs3024548rs3024672rs1805011 | s583s584s585 | 0.299 | 0.718 | 0.478 |
| IL4R | rs3024548rs3024672rs1805011rs1805012 | s583s584s585s586 | 0.216 | 0.418 | 0.795 |
| IL4R | rs3024672rs1805011 | s584s585 | 0.110 | 1.8E-04 | 0.487 |
| IL4R | rs3024672rs1805011rs1805012 | s584s585s586 | 0.083 | 0.042 | 0.268 |
| IL4R | rs3024672rs1805011rs1805012rs1805015 | s584s585s586s587 | 0.275 | 0.163 | 0.320 |
| IL4R | rs1805011rs1805012 | s585s586 | 0.015 | 0.008 | 0.278 |
| IL4R | rs1805011rs1805012rs1805015 | s585s586s587 | 0.114 | 0.075 | 0.595 |
| IL4R | rs1805011rs1805012rs1805015rs1801275 | s585s586s587s588 | 0.264 | 0.121 | 0.524 |
| IL4R | rs1805012rs1805015 | s586s587 | 0.291 | 0.627 | 0.163 |
| IL4R | rs1805012rs1805015rs1801275 | s586s587s588 | 0.043 | 0.079 | 0.130 |
| IL4R | rs1805012rs1805015rs1801275rs1805016 | s586s587s588s589 | 0.081 | 0.108 | 0.228 |
| IL4R | rs1805015rs1801275 | s587s588 | 0.370 | 0.060 | 0.663 |
| IL4R | rs1805015rs1801275rs1805016 | s587s588s589 | 0.427 | 0.093 | 0.645 |
| IL4R | rs1805015rs1801275rs1805016rs1029489 | s587s588s589s590 | 0.142 | 0.005 | 0.304 |
| IL4R | rs1801275rs1805016 | s588s589 | 0.547 | 0.112 | 0.675 |
| IL4R | rs1801275rs1805016rs1029489 | s588s589s590 | 0.065 | 0.054 | 0.076 |
| IL4R | rs1805016rs1029489 | s589s590 | 0.561 | 0.804 | 0.757 |
| IL5RA | rs340833rs4322988 | s211s212 | 0.405 | 0.494 | 0.182 |
| IL5RA | rs340833rs4322988rs6809408 | s211s212s213 | 0.197 | 0.360 | 0.312 |
| IL5RA | rs340833rs4322988rs6809408rs163549 | s211s212s213s214 | 0.029 | 0.168 | 0.033 |
| IL5RA | rs4322988rs6809408 | s212s213 | 0.104 | 0.428 | 0.757 |
| IL5RA | rs4322988rs6809408rs163549 | s212s213s214 | 0.009 | 0.614 | 0.096 |
| IL5RA | rs4322988rs6809408rs163549rs7647903 | s212s213s214s215 | 0.973 | 0.577 | 0.400 |
| IL5RA | rs6809408rs163549 | s213s214 | 4.1E-04 | 0.614 | 0.062 |
| IL5RA | rs6809408rs163549rs7647903 | s213s214s215 | 0.029 | 0.690 | 0.328 |
| IL5RA | rs6809408rs163549rs7647903rs2290610 | s213s214s215s216 | 0.217 | 0.323 | 0.237 |
| IL5RA | rs163549rs7647903 | s214s215 | 0.040 | 0.853 | 0.262 |
| IL5RA | rs163549rs7647903rs2290610 | s214s215s216 | 0.054 | 0.014 | 0.043 |
| IL5RA | rs163549rs7647903rs2290610rs3856847 | s214s215s216s217 | 0.020 | 0.793 | 0.108 |
| IL5RA | rs7647903rs2290610 | s215s216 | 0.722 | 0.001 | 0.563 |
| IL5RA | rs7647903rs2290610rs3856847 | s215s216s217 | 0.304 | 0.377 | 0.014 |
| IL5RA | rs2290610rs3856847 | s216s217 | 0.332 | 0.359 | 0.972 |
| IL6 | rs1554606rs2069845 | s345s346 | 0.764 | 0.297 | 0.504 |
| IL7 | rs2583759rs894221 | s351s352 | 0.449 | 0.685 | 0.671 |
| IL7 | rs2583759rs894221rs2583778 | s351s352s353 | 0.449 | 0.685 | 0.671 |
| IL7 | rs2583759rs894221rs2583778rs6993386 | s351s352s353s354 | 0.200 | 0.791 | 0.416 |
| IL7 | rs894221rs2583778 | s352s353 | 0.449 | 0.689 | 0.661 |
| IL7 | rs894221rs2583778rs6993386 | s352s353s354 | 0.200 | 0.796 | 0.412 |
| IL7 | rs894221rs2583778rs6993386rs1441850 | s352s353s354s355 | 0.200 | 0.796 | 0.411 |
| IL7 | rs2583778rs6993386 | s353s354 | 0.354 | 0.656 | 0.262 |
| IL7 | rs2583778rs6993386rs1441850 | s353s354s355 | 0.200 | 0.796 | 0.411 |
| IL7 | rs2583778rs6993386rs1441850rs1119642 | s353s354s355s356 | 0.536 | 0.593 | 0.708 |
| IL7 | rs6993386rs1441850 | s354s355 | 0.195 | 0.478 | 0.247 |
| IL7 | rs6993386rs1441850rs1119642 | s354s355s356 | 0.587 | 0.859 | 0.858 |
| IL7 | rs6993386rs1441850rs1119642rs2717543 | s354s355s356s357 | 0.458 | 0.018 | 0.420 |
| IL7 | rs1441850rs1119642 | s355s356 | 0.990 | 0.403 | 0.648 |
| IL7 | rs1441850rs1119642rs2717543 | s355s356s357 | 0.809 | 0.313 | 0.203 |
| IL7 | rs1441850rs1119642rs2717543rs2919935 | s355s356s357s358 | 0.495 | 0.472 | 0.343 |
| IL7 | rs1119642rs2717543 | s356s357 | 0.925 | 0.872 | 0.911 |
| IL7 | rs1119642rs2717543rs2919935 | s356s357s358 | 0.682 | 0.704 | 0.638 |
| IL7 | rs1119642rs2717543rs2919935rs10110519 | s356s357s358s359 | 0.772 | 0.682 | 0.701 |
| IL7 | rs2717543rs2919935 | s357s358 | 0.346 | 0.783 | 0.429 |
| IL7 | rs2717543rs2919935rs10110519 | s357s358s359 | 0.499 | 0.779 | 0.473 |
| IL7 | rs2717543rs2919935rs10110519rs2583762 | s357s358s359s360 | 0.499 | 0.779 | 0.473 |
| IL7 | rs2919935rs10110519 | s358s359 | 0.334 | 0.576 | 0.252 |
| IL7 | rs2919935rs10110519rs2583762 | s358s359s360 | 0.499 | 0.795 | 0.483 |
| IL7 | rs2919935rs10110519rs2583762rs11990466 | s358s359s360s361 | 0.348 | 0.874 | 0.459 |
| IL7 | rs10110519rs2583762 | s359s360 | 0.575 | 0.829 | 0.662 |
| IL7 | rs10110519rs2583762rs11990466 | s359s360s361 | 0.332 | 0.892 | 0.495 |
| IL7 | rs10110519rs2583762rs11990466rs6473118 | s359s360s361s362 | 0.435 | 0.451 | 0.621 |
| IL7 | rs2583762rs11990466 | s360s361 | 0.235 | 0.872 | 0.501 |
| IL7 | rs2583762rs11990466rs6473118 | s360s361s362 | 0.294 | 0.458 | 0.458 |
| IL7 | rs2583762rs11990466rs6473118rs11775538 | s360s361s362s363 | 0.294 | 0.455 | 0.460 |
| IL7 | rs11990466rs6473118 | s361s362 | 0.500 | 0.382 | 0.644 |
| IL7 | rs11990466rs6473118rs11775538 | s361s362s363 | 0.500 | 0.380 | 0.559 |
| IL7 | rs11990466rs6473118rs11775538rs11778246 | s361s362s363s364 | 0.500 | 0.380 | 0.559 |
| IL7 | rs6473118rs11775538 | s362s363 | 0.500 | 0.380 | 0.652 |
| IL7 | rs6473118rs11775538rs11778246 | s362s363s364 | 0.500 | 0.380 | 0.559 |
| IL7 | rs11775538rs11778246 | s363s364 | 0.489 | 0.649 | 0.411 |
| IL7R | rs10213865rs10044838 | s267s268 | 0.144 | 0.751 | 0.615 |
| IL7R | rs10213865rs10044838rs1494555 | s267s268s269 | 0.269 | 0.771 | 0.676 |
| IL7R | rs10213865rs10044838rs1494555rs6897932 | s267s268s269s270 | 0.295 | 0.889 | 0.516 |
| IL7R | rs10044838rs1494555 | s268s269 | 0.523 | 0.653 | 0.902 |
| IL7R | rs10044838rs1494555rs6897932 | s268s269s270 | 0.720 | 0.841 | 0.949 |
| IL7R | rs10044838rs1494555rs6897932rs3194051 | s268s269s270s271 | 0.372 | 0.983 | 0.812 |
| IL7R | rs1494555rs6897932 | s269s270 | 0.727 | 0.907 | 0.829 |
| IL7R | rs1494555rs6897932rs3194051 | s269s270s271 | 0.629 | 0.916 | 0.867 |
| IL7R | rs6897932rs3194051 | s270s271 | 0.647 | 0.905 | 0.915 |
| IRAK3 | rs1732886rs1882200 | s519s520 | 0.642 | 1.000 | 0.601 |
| IRAK3 | rs1732886rs1882200rs1152888 | s519s520s521 | 0.865 | 0.003 | 0.846 |
| IRAK3 | rs1732886rs1882200rs1152888rs1152918 | s519s520s521s522 | 6.2E-05 | 1.000 | 7.5E-05 |
| IRAK3 | rs1882200rs1152888 | s520s521 | 0.929 | 0.003 | 0.750 |
| IRAK3 | rs1882200rs1152888rs1152918 | s520s521s522 | 0.937 | 0.157 | 0.502 |
| IRAK3 | rs1152888rs1152918 | s521s522 | 0.950 | 0.171 | 0.399 |
| IRAK4 | rs4251450rs4251459 | s514s515 | 0.631 | 0.596 | 0.562 |
| IRAK4 | rs4251450rs4251459rs3805198 | s514s515s516 | 0.996 | 0.596 | 0.766 |
| IRAK4 | rs4251450rs4251459rs3805198rs4251545 | s514s515s516s517 | 0.755 | 0.386 | 0.336 |
| IRAK4 | rs4251459rs3805198 | s515s516 | 0.961 | 0.474 | 0.590 |
| IRAK4 | rs4251459rs3805198rs4251545 | s515s516s517 | 0.723 | 0.360 | 0.339 |
| IRAK4 | rs4251459rs3805198rs4251545rs4251555 | s515s516s517s518 | 0.747 | 0.360 | 0.304 |
| IRAK4 | rs3805198rs4251545 | s516s517 | 0.737 | 0.360 | 0.348 |
| IRAK4 | rs3805198rs4251545rs4251555 | s516s517s518 | 0.657 | 0.360 | 0.313 |
| IRAK4 | rs4251545rs4251555 | s517s518 | 0.651 | 0.381 | 0.313 |
| IRF4 | rs6930635rs6900384 | s278s279 | 0.328 | 0.461 | 0.822 |
| IRF4 | rs6930635rs6900384rs1473037 | s278s279s280 | 0.464 | 6.3E-04 | 0.764 |
| IRF4 | rs6930635rs6900384rs1473037rs1877179 | s278s279s280s281 | 0.561 | 1.000 | 0.627 |
| IRF4 | rs6900384rs1473037 | s279s280 | 0.156 | 0.772 | 0.088 |
| IRF4 | rs6900384rs1473037rs1877179 | s279s280s281 | 0.430 | 1.000 | 0.376 |
| IRF4 | rs6900384rs1473037rs1877179rs1050975 | s279s280s281s282 | 0.372 | 0.097 | 0.280 |
| IRF4 | rs1473037rs1877179 | s280s281 | 0.568 | 0.788 | 0.957 |
| IRF4 | rs1473037rs1877179rs1050975 | s280s281s282 | 0.404 | 0.482 | 0.024 |
| IRF4 | rs1877179rs1050975 | s281s282 | 0.540 | 0.697 | 0.416 |
| ITGAL | rs11150590rs2285459 | s591s592 | 0.532 | 0.090 | 0.751 |
| ITGAL | rs11150590rs2285459rs6565189 | s591s592s593 | 0.778 | 0.165 | 0.556 |
| ITGAL | rs11150590rs2285459rs6565189rs4243232 | s591s592s593s594 | 0.197 | 0.002 | 0.046 |
| ITGAL | rs2285459rs6565189 | s592s593 | 0.690 | 0.480 | 0.313 |
| ITGAL | rs2285459rs6565189rs4243232 | s592s593s594 | 0.578 | 2.1E-04 | 0.793 |
| ITGAL | rs2285459rs6565189rs4243232rs2230433 | s592s593s594s595 | 0.548 | 0.273 | 0.014 |
| ITGAL | rs6565189rs4243232 | s593s594 | 0.548 | 0.812 | 0.972 |
| ITGAL | rs6565189rs4243232rs2230433 | s593s594s595 | 0.411 | 0.615 | 0.208 |
| ITGAL | rs4243232rs2230433 | s594s595 | 0.568 | 0.564 | 0.381 |
| ITGB2 | rs684rs5030671 | s708s709 | 0.076 | 0.295 | 0.084 |
| ITGB2 | rs684rs5030671rs2235133 | s708s709s710 | 0.016 | 0.179 | 0.001 |
| ITGB2 | rs684rs5030671rs2235133rs760458 | s708s709s710s711 | 0.711 | 1.000 | 0.014 |
| ITGB2 | rs5030671rs2235133 | s709s710 | 0.079 | 0.879 | 0.343 |
| ITGB2 | rs5030671rs2235133rs760458 | s709s710s711 | 0.276 | 1.000 | 0.313 |
| ITGB2 | rs5030671rs2235133rs760458rs3788150 | s709s710s711s712 | 0.373 | 0.129 | 0.229 |
| ITGB2 | rs2235133rs760458 | s710s711 | 0.701 | 1.000 | 0.778 |
| ITGB2 | rs2235133rs760458rs3788150 | s710s711s712 | 0.463 | 0.881 | 0.210 |
| ITGB2 | rs2235133rs760458rs3788150rs3788151 | s710s711s712s713 | 0.172 | 0.646 | 0.369 |
| ITGB2 | rs760458rs3788150 | s711s712 | 0.227 | 0.801 | 0.142 |
| ITGB2 | rs760458rs3788150rs3788151 | s711s712s713 | 0.148 | 0.960 | 0.271 |
| ITGB2 | rs3788150rs3788151 | s712s713 | 0.256 | 0.963 | 0.378 |
| JAK1 | rs2780890rs310244 | s036s037 | 0.516 | 0.412 | 0.813 |
| JAK1 | rs2780890rs310244rs4916005 | s036s037s038 | 0.499 | 0.230 | 0.248 |
| JAK1 | rs2780890rs310244rs4916005rs10789166 | s036s037s038s039 | 0.283 | 0.118 | 0.037 |
| JAK1 | rs310244rs4916005 | s037s038 | 0.365 | 0.432 | 0.440 |
| JAK1 | rs310244rs4916005rs10789166 | s037s038s039 | 0.445 | 0.483 | 0.501 |
| JAK1 | rs310244rs4916005rs10789166rs2780900 | s037s038s039s040 | 0.909 | 0.250 | 0.404 |
| JAK1 | rs4916005rs10789166 | s038s039 | 0.967 | 0.268 | 0.716 |
| JAK1 | rs4916005rs10789166rs2780900 | s038s039s040 | 0.963 | 0.824 | 0.973 |
| JAK1 | rs4916005rs10789166rs2780900rs310228 | s038s039s040s041 | 0.978 | 0.007 | 0.971 |
| JAK1 | rs10789166rs2780900 | s039s040 | 0.883 | 0.833 | 0.975 |
| JAK1 | rs10789166rs2780900rs310228 | s039s040s041 | 0.923 | 0.003 | 0.978 |
| JAK1 | rs10789166rs2780900rs310228rs310236 | s039s040s041s042 | 0.990 | 0.468 | 0.581 |
| JAK1 | rs2780900rs310228 | s040s041 | 0.818 | 0.839 | 0.941 |
| JAK1 | rs2780900rs310228rs310236 | s040s041s042 | 0.732 | 1.000 | 0.997 |
| JAK1 | rs2780900rs310228rs310236rs3790541 | s040s041s042s043 | 0.586 | 0.002 | 0.873 |
| JAK1 | rs310228rs310236 | s041s042 | 0.765 | 0.662 | 0.992 |
| JAK1 | rs310228rs310236rs3790541 | s041s042s043 | 0.605 | 0.319 | 0.999 |
| JAK1 | rs310228rs310236rs3790541rs310199 | s041s042s043s044 | 0.222 | 5.6E-05 | 0.510 |
| JAK1 | rs310236rs3790541 | s042s043 | 0.422 | 0.199 | 0.987 |
| JAK1 | rs310236rs3790541rs310199 | s042s043s044 | 0.125 | 0.017 | 0.291 |
| JAK1 | rs3790541rs310199 | s043s044 | 0.047 | 0.034 | 0.663 |
| JAK3 | rs3212752rs867174 | s645s646 | 0.612 | 0.100 | 0.912 |
| JAK3 | rs3212752rs867174rs3212711 | s645s646s647 | 0.615 | 0.008 | 0.867 |
| JAK3 | rs3212752rs867174rs3212711rs3212701 | s645s646s647s648 | 0.574 | 2.9E-07 | 0.008 |
| JAK3 | rs867174rs3212711 | s646s647 | 0.648 | 0.152 | 0.251 |
| JAK3 | rs867174rs3212711rs3212701 | s646s647s648 | 0.729 | 0.105 | 0.022 |
| JAK3 | rs867174rs3212711rs3212701rs7250423 | s646s647s648s649 | 0.686 | 0.002 | 0.006 |
| JAK3 | rs3212711rs3212701 | s647s648 | 0.435 | 0.002 | 0.003 |
| JAK3 | rs3212711rs3212701rs7250423 | s647s648s649 | 0.426 | 0.002 | 5.1E-07 |
| JAK3 | rs3212701rs7250423 | s648s649 | 0.458 | 0.091 | 0.180 |
| LGMN | rs2250672rs2402189 | s550s551 | 0.139 | 0.567 | 0.090 |
| LGMN | rs2250672rs2402189rs3818320 | s550s551s552 | 0.242 | 0.773 | 0.176 |
| LGMN | rs2250672rs2402189rs3818320rs2236264 | s550s551s552s553 | 0.387 | 0.930 | 0.330 |
| LGMN | rs2402189rs3818320 | s551s552 | 0.192 | 0.566 | 0.133 |
| LGMN | rs2402189rs3818320rs2236264 | s551s552s553 | 0.557 | 1.000 | 0.524 |
| LGMN | rs2402189rs3818320rs2236264rs1242102 | s551s552s553s554 | 0.666 | 0.643 | 0.467 |
| LGMN | rs3818320rs2236264 | s552s553 | 0.943 | 0.443 | 0.688 |
| LGMN | rs3818320rs2236264rs1242102 | s552s553s554 | 0.938 | 1.000 | 0.102 |
| LGMN | rs3818320rs2236264rs1242102rs1242095 | s552s553s554s555 | 0.727 | 6.2E-06 | 0.854 |
| LGMN | rs2236264rs1242102 | s553s554 | 0.835 | 0.513 | 0.563 |
| LGMN | rs2236264rs1242102rs1242095 | s553s554s555 | 0.386 | 0.433 | 0.826 |
| LGMN | rs1242102rs1242095 | s554s555 | 0.476 | 0.233 | 0.786 |
| MAP2K3 | rs1466314rs8074866 | s604s605 | 0.336 | 0.763 | 0.870 |
| MAP2K3 | rs1466314rs8074866rs3760201 | s604s605s606 | 0.567 | 0.884 | 0.940 |
| MAP2K3 | rs1466314rs8074866rs3760201rs9899521 | s604s605s606s608 | 0.628 | 0.453 | 0.889 |
| MAP2K3 | rs8074866rs3760201 | s605s606 | 0.404 | 0.950 | 0.619 |
| MAP2K3 | rs8074866rs3760201rs9899521 | s605s606s608 | 0.812 | 0.134 | 0.373 |
| MAP2K3 | rs8074866rs3760201rs9899521rs9901404 | s605s606s608s609 | 0.204 | 0.367 | 0.659 |
| MAP2K3 | rs3760201rs9899521 | s606s608 | 0.557 | 0.231 | 0.723 |
| MAP2K3 | rs3760201rs9899521rs9901404 | s606s608s609 | 0.045 | 0.671 | 0.476 |
| MAP2K3 | rs3760201rs9899521rs9901404rs9909362 | s606s608s609s610 | 0.614 | 1.000 | 0.916 |
| MAP2K3 | rs9899521rs9901404 | s608s609 | 0.783 | 0.778 | 0.647 |
| MAP2K3 | rs9899521rs9901404rs9909362 | s608s609s610 | 0.819 | 0.676 | 0.514 |
| MAP2K3 | rs9901404rs9909362 | s609s610 | 0.507 | 1.000 | 0.439 |
| MAP2K4 | rs7216687rs2108496 | s597s598 | 0.201 | 1.000 | 0.428 |
| MAP2K4 | rs7216687rs2108496rs8080333 | s597s598s599 | 0.134 | 0.679 | 0.438 |
| MAP2K4 | rs7216687rs2108496rs8080333rs8065164 | s597s598s599s600 | 0.016 | 0.691 | 0.186 |
| MAP2K4 | rs2108496rs8080333 | s598s599 | 0.652 | 0.002 | 0.460 |
| MAP2K4 | rs2108496rs8080333rs8065164 | s598s599s600 | 0.610 | 0.003 | 0.180 |
| MAP2K4 | rs2108496rs8080333rs8065164rs8064513 | s598s599s600s601 | 0.610 | 0.002 | 0.372 |
| MAP2K4 | rs8080333rs8065164 | s599s600 | 0.707 | 0.747 | 0.962 |
| MAP2K4 | rs8080333rs8065164rs8064513 | s599s600s601 | 0.761 | 1.000 | 0.996 |
| MAP2K4 | rs8080333rs8065164rs8064513rs7207011 | s599s600s601s602 | 0.678 | 0.653 | 0.932 |
| MAP2K4 | rs8065164rs8064513 | s600s601 | 0.523 | 0.245 | 0.751 |
| MAP2K4 | rs8065164rs8064513rs7207011 | s600s601s602 | 0.600 | 0.590 | 0.729 |
| MAP2K4 | rs8065164rs8064513rs7207011rs1870584 | s600s601s602s603 | 0.442 | 0.646 | 0.432 |
| MAP2K4 | rs8064513rs7207011 | s601s602 | 0.719 | 0.569 | 0.755 |
| MAP2K4 | rs8064513rs7207011rs1870584 | s601s602s603 | 0.532 | 0.780 | 0.533 |
| MAP2K4 | rs7207011rs1870584 | s602s603 | 0.492 | 0.899 | 0.570 |
| MAP2K6 | rs2034100rs817565 | s620s621 | 0.182 | 0.975 | 0.444 |
| MAP2K6 | rs2034100rs817565rs11869348 | s620s621s622 | 0.154 | 0.155 | 0.033 |
| MAP2K6 | rs2034100rs817565rs11869348rs7213686 | s620s621s622s623 | 0.325 | 2.5E-05 | 0.001 |
| MAP2K6 | rs817565rs11869348 | s621s622 | 0.141 | 0.155 | 0.021 |
| MAP2K6 | rs817565rs11869348rs7213686 | s621s622s623 | 0.313 | 0.005 | 0.007 |
| MAP2K6 | rs817565rs11869348rs7213686rs6501328 | s621s622s623s624 | 0.478 | 0.004 | 0.016 |
| MAP2K6 | rs11869348rs7213686 | s622s623 | 0.410 | 0.032 | 0.376 |
| MAP2K6 | rs11869348rs7213686rs6501328 | s622s623s624 | 0.192 | 0.046 | 0.661 |
| MAP2K6 | rs11869348rs7213686rs6501328rs2028049 | s622s623s624s625 | 0.282 | 0.025 | 0.577 |
| MAP2K6 | rs7213686rs6501328 | s623s624 | 0.472 | 0.592 | 0.697 |
| MAP2K6 | rs7213686rs6501328rs2028049 | s623s624s625 | 0.061 | 0.219 | 0.195 |
| MAP2K6 | rs7213686rs6501328rs2028049rs11869073 | s623s624s625s626 | 0.092 | 0.743 | 0.152 |
| MAP2K6 | rs6501328rs2028049 | s624s625 | 0.096 | 0.196 | 0.461 |
| MAP2K6 | rs6501328rs2028049rs11869073 | s624s625s626 | 0.103 | 0.600 | 0.891 |
| MAP2K6 | rs6501328rs2028049rs11869073rs8078890 | s624s625s626s627 | 0.013 | 0.862 | 0.179 |
| MAP2K6 | rs2028049rs11869073 | s625s626 | 0.576 | 0.686 | 0.425 |
| MAP2K6 | rs2028049rs11869073rs8078890 | s625s626s627 | 0.643 | 1.000 | 0.496 |
| MAP2K6 | rs2028049rs11869073rs8078890rs6501329 | s625s626s627s629 | 0.302 | 0.024 | 1.000 |
| MAP2K6 | rs11869073rs8078890 | s626s627 | 0.845 | 0.539 | 0.253 |
| MAP2K6 | rs11869073rs8078890rs6501329 | s626s627s629 | 0.602 | 0.323 | 0.924 |
| MAP2K6 | rs11869073rs8078890rs6501329rs2715824 | s626s627s629s630 | 0.685 | 1.000 | 0.927 |
| MAP2K6 | rs8078890rs6501329 | s627s629 | 0.792 | 0.961 | 0.980 |
| MAP2K6 | rs8078890rs6501329rs2715824 | s627s629s630 | 0.316 | 0.802 | 0.826 |
| MAP2K6 | rs8078890rs6501329rs2715824rs2251862 | s627s629s630s631 | 0.600 | 0.445 | 0.898 |
| MAP2K6 | rs6501329rs2715824 | s629s630 | 0.380 | 0.875 | 0.762 |
| MAP2K6 | rs6501329rs2715824rs2251862 | s629s630s631 | 0.664 | 0.926 | 0.912 |
| MAP2K6 | rs6501329rs2715824rs2251862rs2715832 | s629s630s631s632 | 0.791 | 0.833 | 0.854 |
| MAP2K6 | rs2715824rs2251862 | s630s631 | 0.668 | 0.556 | 0.999 |
| MAP2K6 | rs2715824rs2251862rs2715832 | s630s631s632 | 0.861 | 0.763 | 0.804 |
| MAP2K6 | rs2715824rs2251862rs2715832rs756944 | s630s631s632s633 | 0.863 | 0.596 | 0.653 |
| MAP2K6 | rs2251862rs2715832 | s631s632 | 0.828 | 0.001 | 0.579 |
| MAP2K6 | rs2251862rs2715832rs756944 | s631s632s633 | 0.628 | 0.821 | 0.705 |
| MAP2K6 | rs2251862rs2715832rs756944rs2716191 | s631s632s633s634 | 0.216 | 0.730 | 0.673 |
| MAP2K6 | rs2715832rs756944 | s632s633 | 0.805 | 1.000 | 0.624 |
| MAP2K6 | rs2715832rs756944rs2716191 | s632s633s634 | 0.156 | 0.708 | 0.702 |
| MAP2K6 | rs756944rs2716191 | s633s634 | 0.369 | 0.769 | 0.718 |
| MAPK8 | rs3827680rs10857565 | s419s420 | 0.024 | 0.253 | 0.006 |
| MAPK8 | rs3827680rs10857565rs11101318 | s419s420s421 | 0.024 | 0.327 | 0.006 |
| MAPK8 | rs3827680rs10857565rs11101318rs10508901 | s419s420s421s422 | 0.032 | 0.127 | 0.034 |
| MAPK8 | rs10857565rs11101318 | s420s421 | 0.023 | 0.284 | 0.006 |
| MAPK8 | rs10857565rs11101318rs10508901 | s420s421s422 | 0.033 | 0.132 | 0.029 |
| MAPK8 | rs10857565rs11101318rs10508901rs10508903 | s420s421s422s423 | 0.022 | 0.281 | 0.004 |
| MAPK8 | rs11101318rs10508901 | s421s422 | 0.022 | 0.191 | 0.006 |
| MAPK8 | rs11101318rs10508901rs10508903 | s421s422s423 | 0.022 | 0.339 | 0.022 |
| MAPK8 | rs11101318rs10508901rs10508903rs9284 | s421s422s423s424 | 0.022 | 0.338 | 0.021 |
| MAPK8 | rs10508901rs10508903 | s422s423 | 0.119 | 0.139 | 0.015 |
| MAPK8 | rs10508901rs10508903rs9284 | s422s423s424 | 0.144 | 0.136 | 0.026 |
| MAPK8 | rs10508903rs9284 | s423s424 | 0.050 | 0.215 | 0.026 |
| MS4A1 | rs948312rs7126354 | s473s474 | 0.579 | 0.698 | 0.802 |
| MS4A1 | rs948312rs7126354rs4939364 | s473s474s475 | 0.205 | 0.870 | 0.020 |
| MS4A1 | rs948312rs7126354rs4939364rs1051756 | s473s474s475s476 | 0.186 | 0.771 | 0.002 |
| MS4A1 | rs7126354rs4939364 | s474s475 | 0.238 | 0.688 | 0.003 |
| MS4A1 | rs7126354rs4939364rs1051756 | s474s475s476 | 0.204 | 0.684 | 2.2E-04 |
| MS4A1 | rs4939364rs1051756 | s475s476 | 0.275 | 0.752 | 0.391 |
| MS4A4A | rs2017549rs1026250 | s467s468 | 0.570 | 0.285 | 0.451 |
| MS4A4A | rs2017549rs1026250rs4939331 | s467s468s469 | 0.470 | 0.173 | 0.706 |
| MS4A4A | rs2017549rs1026250rs4939331rs10750931 | s467s468s469s470 | 0.516 | 1.000 | 0.681 |
| MS4A4A | rs1026250rs4939331 | s468s469 | 0.679 | 0.144 | 0.466 |
| MS4A4A | rs1026250rs4939331rs10750931 | s468s469s470 | 0.784 | 0.264 | 0.629 |
| MS4A4A | rs1026250rs4939331rs10750931rs6591561 | s468s469s470s471 | 0.863 | 0.777 | 0.622 |
| MS4A4A | rs4939331rs10750931 | s469s470 | 0.603 | 0.112 | 0.393 |
| MS4A4A | rs4939331rs10750931rs6591561 | s469s470s471 | 0.758 | 0.531 | 0.372 |
| MS4A4A | rs4939331rs10750931rs6591561rs10681 | s469s470s471s472 | 0.659 | 0.479 | 0.275 |
| MS4A4A | rs10750931rs6591561 | s470s471 | 0.637 | 0.389 | 0.468 |
| MS4A4A | rs10750931rs6591561rs10681 | s470s471s472 | 0.535 | 0.352 | 0.384 |
| MS4A4A | rs6591561rs10681 | s471s472 | 0.644 | 0.355 | 0.410 |
| MX1 | rs461093rs468672 | s698s699 | 0.716 | 0.047 | 0.513 |
| MX1 | rs461093rs468672rs8132871 | s698s699s700 | 0.671 | 0.139 | 0.508 |
| MX1 | rs461093rs468672rs8132871rs468440 | s698s699s700s701 | 0.142 | 0.289 | 0.398 |
| MX1 | rs468672rs8132871 | s699s700 | 0.714 | 0.452 | 0.915 |
| MX1 | rs468672rs8132871rs468440 | s699s700s701 | 0.179 | 0.820 | 0.334 |
| MX1 | rs468672rs8132871rs468440rs469390 | s699s700s701s702 | 0.742 | 0.644 | 0.667 |
| MX1 | rs8132871rs468440 | s700s701 | 0.457 | 0.865 | 0.818 |
| MX1 | rs8132871rs468440rs469390 | s700s701s702 | 0.668 | 0.811 | 0.780 |
| MX1 | rs8132871rs468440rs469390rs1050008 | s700s701s702s703 | 0.076 | 0.831 | 0.445 |
| MX1 | rs468440rs469390 | s701s702 | 0.652 | 0.700 | 0.702 |
| MX1 | rs468440rs469390rs1050008 | s701s702s703 | 0.013 | 0.842 | 1.2E-05 |
| MX1 | rs468440rs469390rs1050008rs1557370 | s701s702s703s704 | 1.000 | 0.965 | 0.590 |
| MX1 | rs469390rs1050008 | s702s703 | 0.068 | 0.867 | 0.136 |
| MX1 | rs469390rs1050008rs1557370 | s702s703s704 | 0.215 | 0.959 | 0.245 |
| MX1 | rs1050008rs1557370 | s703s704 | 0.878 | 0.773 | 0.660 |
| NFKB1 | rs4647992rs230529 | s243s244 | 0.578 | 0.427 | 0.975 |
| NFKB1 | rs4647992rs230529rs230521 | s243s244s245 | 0.578 | 0.427 | 0.975 |
| NFKB1 | rs4647992rs230529rs230521rs4648037 | s243s244s245s246 | 0.640 | 0.654 | 0.999 |
| NFKB1 | rs230529rs230521 | s244s245 | 0.749 | 0.454 | 0.778 |
| NFKB1 | rs230529rs230521rs4648037 | s244s245s246 | 0.665 | 0.704 | 0.968 |
| NFKB1 | rs230529rs230521rs4648037rs3774956 | s244s245s246s247 | 0.665 | 0.721 | 0.981 |
| NFKB1 | rs230521rs4648037 | s245s246 | 0.665 | 0.704 | 0.968 |
| NFKB1 | rs230521rs4648037rs3774956 | s245s246s247 | 0.665 | 0.721 | 0.981 |
| NFKB1 | rs230521rs4648037rs3774956rs1020760 | s245s246s247s248 | 0.665 | 0.721 | 0.981 |
| NFKB1 | rs4648037rs3774956 | s246s247 | 0.665 | 0.719 | 0.974 |
| NFKB1 | rs4648037rs3774956rs1020760 | s246s247s248 | 0.665 | 0.719 | 0.974 |
| NFKB1 | rs4648037rs3774956rs1020760rs4648072 | s246s247s248s249 | 0.458 | 0.708 | 0.937 |
| NFKB1 | rs3774956rs1020760 | s247s248 | 0.749 | 0.479 | 0.800 |
| NFKB1 | rs3774956rs1020760rs4648072 | s247s248s249 | 0.309 | 0.662 | 0.835 |
| NFKB1 | rs3774956rs1020760rs4648072rs230547 | s247s248s249s250 | 0.404 | 0.991 | 0.604 |
| NFKB1 | rs1020760rs4648072 | s248s249 | 0.309 | 0.607 | 0.860 |
| NFKB1 | rs1020760rs4648072rs230547 | s248s249s250 | 0.404 | 0.988 | 0.613 |
| NFKB1 | rs1020760rs4648072rs230547rs1609798 | s248s249s250s251 | 0.276 | 0.765 | 0.792 |
| NFKB1 | rs4648072rs230547 | s249s250 | 0.211 | 0.630 | 0.656 |
| NFKB1 | rs4648072rs230547rs1609798 | s249s250s251 | 0.340 | 2.5E-06 | 0.844 |
| NFKB1 | rs230547rs1609798 | s250s251 | 0.409 | 0.800 | 0.919 |
| NFKBIA | rs4982269rs696 | s546s547 | 0.158 | 0.261 | 0.648 |
| NFKBIA | rs4982269rs696rs2233409 | s546s547s548 | 0.481 | 0.613 | 0.099 |
| NFKBIA | rs4982269rs696rs2233409rs3138050 | s546s547s548s549 | 0.690 | 0.221 | 0.207 |
| NFKBIA | rs696rs2233409 | s547s548 | 0.318 | 0.512 | 0.124 |
| NFKBIA | rs696rs2233409rs3138050 | s547s548s549 | 0.720 | 0.378 | 0.106 |
| NFKBIA | rs2233409rs3138050 | s548s549 | 0.056 | 0.904 | 0.238 |
| NFKBIE | rs2282151rs730775 | s298s299 | 0.984 | 0.349 | 0.567 |
| NFKBIE | rs2282151rs730775rs2233424 | s298s299s300 | 0.988 | 0.021 | 1.000 |
| NFKBIE | rs730775rs2233424 | s299s300 | 0.882 | 0.054 | 0.385 |
| NFKBIL2 | rs3862193rs4082353 | s365s366 | 0.110 | 0.791 | 0.243 |
| NFKBIL2 | rs3862193rs4082353rs2306384 | s365s366s367 | 0.143 | 0.800 | 0.232 |
| NFKBIL2 | rs3862193rs4082353rs2306384rs4925858 | s365s366s367s369 | 0.185 | 0.072 | 0.018 |
| NFKBIL2 | rs4082353rs2306384 | s366s367 | 0.126 | 0.672 | 0.319 |
| NFKBIL2 | rs4082353rs2306384rs4925858 | s366s367s369 | 0.204 | 0.003 | 0.003 |
| NFKBIL2 | rs2306384rs4925858 | s367s369 | 0.287 | 0.840 | 0.226 |
| OAS1 | rs10744785rs3741981 | s533s534 | 0.592 | 0.762 | 0.866 |
| OAS1 | rs10744785rs3741981rs2285934 | s533s534s535 | 0.815 | 0.798 | 0.888 |
| OAS1 | rs10744785rs3741981rs2285934rs10774671 | s533s534s535s536 | 0.616 | 0.850 | 0.620 |
| OAS1 | rs3741981rs2285934 | s534s535 | 0.748 | 0.761 | 0.769 |
| OAS1 | rs3741981rs2285934rs10774671 | s534s535s536 | 0.470 | 0.946 | 0.492 |
| OAS1 | rs2285934rs10774671 | s535s536 | 0.391 | 0.727 | 0.247 |
| PAX5 | rs4880018rs3860999 | s370s371 | 0.145 | 0.262 | 0.932 |
| PAX5 | rs4880018rs3860999rs4510947 | s370s371s372 | 0.523 | 0.234 | 0.691 |
| PAX5 | rs4880018rs3860999rs4510947rs10758405 | s370s371s372s373 | 0.245 | 0.006 | 0.970 |
| PAX5 | rs3860999rs4510947 | s371s372 | 0.902 | 0.024 | 0.971 |
| PAX5 | rs3860999rs4510947rs10758405 | s371s372s373 | 0.373 | 0.244 | 0.569 |
| PAX5 | rs3860999rs4510947rs10758405rs4880023 | s371s372s373s374 | 0.699 | 0.367 | 0.308 |
| PAX5 | rs4510947rs10758405 | s372s373 | 0.470 | 0.636 | 0.880 |
| PAX5 | rs4510947rs10758405rs4880023 | s372s373s374 | 0.462 | 0.291 | 0.388 |
| PAX5 | rs4510947rs10758405rs4880023rs7850607 | s372s373s374s375 | 0.464 | 0.093 | 0.129 |
| PAX5 | rs10758405rs4880023 | s373s374 | 0.151 | 0.849 | 0.289 |
| PAX5 | rs10758405rs4880023rs7850607 | s373s374s375 | 0.234 | 0.581 | 0.112 |
| PAX5 | rs10758405rs4880023rs7850607rs10511935 | s373s374s375s376 | 0.092 | 0.446 | 0.027 |
| PAX5 | rs4880023rs7850607 | s374s375 | 0.354 | 0.491 | 0.188 |
| PAX5 | rs4880023rs7850607rs10511935 | s374s375s376 | 0.441 | 0.305 | 0.113 |
| PAX5 | rs4880023rs7850607rs10511935rs12683779 | s374s375s376s377 | 0.146 | 0.331 | 0.090 |
| PAX5 | rs7850607rs10511935 | s375s376 | 0.392 | 0.485 | 0.220 |
| PAX5 | rs7850607rs10511935rs12683779 | s375s376s377 | 0.183 | 0.869 | 0.278 |
| PAX5 | rs7850607rs10511935rs12683779rs3780138 | s375s376s377s378 | 0.021 | 0.870 | 0.102 |
| PAX5 | rs10511935rs12683779 | s376s377 | 0.930 | 0.916 | 0.791 |
| PAX5 | rs10511935rs12683779rs3780138 | s376s377s378 | 0.006 | 0.967 | 0.149 |
| PAX5 | rs10511935rs12683779rs3780138rs10758412 | s376s377s378s379 | 0.008 | 0.982 | 0.059 |
| PAX5 | rs12683779rs3780138 | s377s378 | 7.3E-04 | 0.937 | 0.015 |
| PAX5 | rs12683779rs3780138rs10758412 | s377s378s379 | 0.001 | 0.692 | 0.028 |
| PAX5 | rs12683779rs3780138rs10758412rs3758161 | s377s378s379s380 | 0.018 | 0.799 | 0.164 |
| PAX5 | rs3780138rs10758412 | s378s379 | 3.3E-04 | 0.341 | 0.009 |
| PAX5 | rs3780138rs10758412rs3758161 | s378s379s380 | 0.015 | 0.722 | 0.116 |
| PAX5 | rs3780138rs10758412rs3758161rs3824340 | s378s379s380s381 | 0.016 | 0.617 | 0.265 |
| PAX5 | rs10758412rs3758161 | s379s380 | 0.270 | 0.742 | 0.257 |
| PAX5 | rs10758412rs3758161rs3824340 | s379s380s381 | 0.105 | 0.774 | 0.083 |
| PAX5 | rs10758412rs3758161rs3824340rs3758163 | s379s380s381s382 | 0.473 | 0.422 | 0.445 |
| PAX5 | rs3758161rs3824340 | s380s381 | 0.307 | 0.558 | 0.730 |
| PAX5 | rs3758161rs3824340rs3758163 | s380s381s382 | 0.490 | 0.657 | 0.795 |
| PAX5 | rs3758161rs3824340rs3758163rs4515644 | s380s381s382s383 | 0.325 | 0.693 | 0.892 |
| PAX5 | rs3824340rs3758163 | s381s382 | 0.399 | 0.543 | 0.650 |
| PAX5 | rs3824340rs3758163rs4515644 | s381s382s383 | 0.405 | 0.636 | 0.888 |
| PAX5 | rs3824340rs3758163rs4515644rs3780151 | s381s382s383s384 | 0.187 | 1.000 | 0.268 |
| PAX5 | rs3758163rs4515644 | s382s383 | 0.901 | 0.648 | 0.898 |
| PAX5 | rs3758163rs4515644rs3780151 | s382s383s384 | 0.683 | 0.445 | 0.510 |
| PAX5 | rs3758163rs4515644rs3780151rs7032313 | s382s383s384s385 | 0.548 | 0.765 | 0.543 |
| PAX5 | rs4515644rs3780151 | s383s384 | 0.807 | 0.169 | 0.286 |
| PAX5 | rs4515644rs3780151rs7032313 | s383s384s385 | 0.799 | 0.195 | 0.313 |
| PAX5 | rs4515644rs3780151rs7032313rs13288123 | s383s384s385s386 | 0.310 | 0.317 | 0.428 |
| PAX5 | rs3780151rs7032313 | s384s385 | 0.360 | 0.617 | 0.319 |
| PAX5 | rs3780151rs7032313rs13288123 | s384s385s386 | 0.714 | 0.738 | 0.537 |
| PAX5 | rs3780151rs7032313rs13288123rs3780154 | s384s385s386s387 | 0.825 | 0.629 | 0.702 |
| PAX5 | rs7032313rs13288123 | s385s386 | 0.705 | 0.733 | 0.533 |
| PAX5 | rs7032313rs13288123rs3780154 | s385s386s387 | 0.812 | 0.628 | 0.703 |
| PAX5 | rs7032313rs13288123rs3780154rs3780157 | s385s386s387s388 | 0.343 | 0.537 | 0.764 |
| PAX5 | rs13288123rs3780154 | s386s387 | 0.891 | 0.397 | 0.484 |
| PAX5 | rs13288123rs3780154rs3780157 | s386s387s388 | 0.207 | 0.659 | 0.661 |
| PAX5 | rs13288123rs3780154rs3780157rs11998778 | s386s387s388s389 | 0.227 | 0.696 | 0.373 |
| PAX5 | rs3780154rs3780157 | s387s388 | 0.120 | 0.779 | 0.499 |
| PAX5 | rs3780154rs3780157rs11998778 | s387s388s389 | 0.440 | 0.817 | 0.748 |
| PAX5 | rs3780154rs3780157rs11998778rs7859088 | s387s388s389s390 | 0.129 | 0.324 | 0.333 |
| PAX5 | rs3780157rs11998778 | s388s389 | 0.194 | 0.827 | 0.563 |
| PAX5 | rs3780157rs11998778rs7859088 | s388s389s390 | 0.528 | 0.607 | 0.189 |
| PAX5 | rs3780157rs11998778rs7859088rs2039217 | s388s389s390s391 | 0.066 | 0.656 | 0.501 |
| PAX5 | rs11998778rs7859088 | s389s390 | 0.793 | 0.328 | 0.584 |
| PAX5 | rs11998778rs7859088rs2039217 | s389s390s391 | 0.803 | 0.387 | 0.657 |
| PAX5 | rs11998778rs7859088rs2039217rs10758418 | s389s390s391s392 | 0.851 | 0.294 | 0.620 |
| PAX5 | rs7859088rs2039217 | s390s391 | 0.756 | 0.065 | 0.418 |
| PAX5 | rs7859088rs2039217rs10758418 | s390s391s392 | 0.945 | 0.043 | 0.057 |
| PAX5 | rs7859088rs2039217rs10758418rs3780169 | s390s391s392s393 | 0.132 | 0.036 | 0.444 |
| PAX5 | rs2039217rs10758418 | s391s392 | 0.914 | 0.155 | 0.071 |
| PAX5 | rs2039217rs10758418rs3780169 | s391s392s393 | 0.046 | 0.019 | 0.233 |
| PAX5 | rs2039217rs10758418rs3780169rs3018183 | s391s392s393s394 | 0.210 | 0.125 | 0.643 |
| PAX5 | rs10758418rs3780169 | s392s393 | 0.074 | 0.256 | 0.635 |
| PAX5 | rs10758418rs3780169rs3018183 | s392s393s394 | 0.303 | 0.241 | 0.793 |
| PAX5 | rs10758418rs3780169rs3018183rs2282077 | s392s393s394s395 | 0.396 | 0.216 | 0.632 |
| PAX5 | rs3780169rs3018183 | s393s394 | 0.487 | 0.003 | 0.606 |
| PAX5 | rs3780169rs3018183rs2282077 | s393s394s395 | 0.690 | 0.168 | 0.726 |
| PAX5 | rs3780169rs3018183rs2282077rs883966 | s393s394s395s396 | 0.286 | 0.110 | 0.699 |
| PAX5 | rs3018183rs2282077 | s394s395 | 0.618 | 0.088 | 0.571 |
| PAX5 | rs3018183rs2282077rs883966 | s394s395s396 | 0.047 | 0.149 | 0.656 |
| PAX5 | rs3018183rs2282077rs883966rs1329573 | s394s395s396s397 | 0.283 | 0.002 | 0.831 |
| PAX5 | rs2282077rs883966 | s395s396 | 0.009 | 0.311 | 0.607 |
| PAX5 | rs2282077rs883966rs1329573 | s395s396s397 | 0.102 | 1.000 | 1.000 |
| PAX5 | rs2282077rs883966rs1329573rs7020413 | s395s396s397s398 | 0.128 | 0.188 | 0.769 |
| PAX5 | rs883966rs1329573 | s396s397 | 0.276 | 0.867 | 0.802 |
| PAX5 | rs883966rs1329573rs7020413 | s396s397s398 | 0.143 | 0.236 | 0.603 |
| PAX5 | rs883966rs1329573rs7020413rs944066 | s396s397s398s399 | 0.691 | 1.000 | 0.955 |
| PAX5 | rs1329573rs7020413 | s397s398 | 0.948 | 0.349 | 0.430 |
| PAX5 | rs1329573rs7020413rs944066 | s397s398s399 | 0.983 | 0.500 | 0.606 |
| PAX5 | rs1329573rs7020413rs944066rs3739440 | s397s398s399s400 | 0.987 | 7.0E-04 | 0.908 |
| PAX5 | rs7020413rs944066 | s398s399 | 0.644 | 0.524 | 0.295 |
| PAX5 | rs7020413rs944066rs3739440 | s398s399s400 | 0.750 | 0.152 | 0.345 |
| PAX5 | rs944066rs3739440 | s399s400 | 0.592 | 0.006 | 0.596 |
| PRDM1 | rs6923419rs811925 | s302s303 | 0.385 | 0.105 | 0.728 |
| PRDM1 | rs6923419rs811925rs530239 | s302s303s304 | 0.213 | 0.079 | 0.905 |
| PRDM1 | rs6923419rs811925rs530239rs573869 | s302s303s304s305 | 0.098 | 6.0E-04 | 0.838 |
| PRDM1 | rs811925rs530239 | s303s304 | 0.694 | 0.029 | 0.316 |
| PRDM1 | rs811925rs530239rs573869 | s303s304s305 | 0.629 | 0.020 | 0.135 |
| PRDM1 | rs530239rs573869 | s304s305 | 0.974 | 9.2E-11 | 0.243 |
| PTPN22 | rs2476599rs1217388 | s054s055 | 0.364 | 0.912 | 0.399 |
| PTPN22 | rs2476599rs1217388rs1217395 | s054s055s056 | 0.293 | 0.968 | 0.469 |
| PTPN22 | rs2476599rs1217388rs1217395rs2476602 | s054s055s056s058 | 0.293 | 0.968 | 0.469 |
| PTPN22 | rs1217388rs1217395 | s055s056 | 0.745 | 1.000 | 0.724 |
| PTPN22 | rs1217388rs1217395rs2476602 | s055s056s058 | 0.293 | 0.968 | 0.469 |
| PTPN22 | rs1217388rs1217395rs2476602rs2488458 | s055s056s058s059 | 0.308 | 0.986 | 0.712 |
| PTPN22 | rs1217395rs2476602 | s056s058 | 0.293 | 0.970 | 0.413 |
| PTPN22 | rs1217395rs2476602rs2488458 | s056s058s059 | 0.308 | 0.988 | 0.650 |
| PTPN22 | rs2476602rs2488458 | s058s059 | 0.379 | 0.919 | 0.482 |
| PTPRC | rs6673408rs1326273 | s080s081 | 0.314 | 0.679 | 0.395 |
| PTPRC | rs6673408rs1326273rs1052238 | s080s081s082 | 0.124 | 0.670 | 0.308 |
| PTPRC | rs6673408rs1326273rs1052238rs9803750 | s080s081s082s083 | 0.237 | 0.134 | 0.837 |
| PTPRC | rs1326273rs1052238 | s081s082 | 0.097 | 0.648 | 0.180 |
| PTPRC | rs1326273rs1052238rs9803750 | s081s082s083 | 0.291 | 0.596 | 0.884 |
| PTPRC | rs1326273rs1052238rs9803750rs9803978 | s081s082s083s084 | 0.441 | 0.387 | 0.895 |
| PTPRC | rs1052238rs9803750 | s082s083 | 0.090 | 0.653 | 0.317 |
| PTPRC | rs1052238rs9803750rs9803978 | s082s083s084 | 0.444 | 0.680 | 0.200 |
| PTPRC | rs1052238rs9803750rs9803978rs1326279 | s082s083s084s085 | 0.212 | 0.698 | 0.358 |
| PTPRC | rs9803750rs9803978 | s083s084 | 0.291 | 0.350 | 0.900 |
| PTPRC | rs9803750rs9803978rs1326279 | s083s084s085 | 0.769 | 0.336 | 0.888 |
| PTPRC | rs9803750rs9803978rs1326279rs2359952 | s083s084s085s086 | 0.760 | 0.427 | 0.620 |
| PTPRC | rs9803978rs1326279 | s084s085 | 0.293 | 0.415 | 0.917 |
| PTPRC | rs9803978rs1326279rs2359952 | s084s085s086 | 0.831 | 0.484 | 0.594 |
| PTPRC | rs9803978rs1326279rs2359952rs3767747 | s084s085s086s087 | 0.975 | 0.437 | 0.576 |
| PTPRC | rs1326279rs2359952 | s085s086 | 0.664 | 0.378 | 0.451 |
| PTPRC | rs1326279rs2359952rs3767747 | s085s086s087 | 0.871 | 0.626 | 0.578 |
| PTPRC | rs1326279rs2359952rs3767747rs10919557 | s085s086s087s088 | 0.821 | 0.726 | 0.468 |
| PTPRC | rs2359952rs3767747 | s086s087 | 0.995 | 0.567 | 0.747 |
| PTPRC | rs2359952rs3767747rs10919557 | s086s087s088 | 0.985 | 0.614 | 0.671 |
| PTPRC | rs2359952rs3767747rs10919557rs1011338 | s086s087s088s093 | 0.987 | 0.258 | 0.805 |
| PTPRC | rs3767747rs10919557 | s087s088 | 0.912 | 0.368 | 0.494 |
| PTPRC | rs3767747rs10919557rs1011338 | s087s088s093 | 0.865 | 0.630 | 0.932 |
| PTPRC | rs3767747rs10919557rs1011338rs1932433 | s087s088s093s094 | 0.958 | 0.530 | 0.965 |
| PTPRC | rs10919557rs1011338 | s088s093 | 0.373 | 0.141 | 0.731 |
| PTPRC | rs10919557rs1011338rs1932433 | s088s093s094 | 0.381 | 0.251 | 0.498 |
| PTPRC | rs10919557rs1011338rs1932433rs6683595 | s088s093s094s095 | 0.579 | 0.221 | 0.012 |
| PTPRC | rs1011338rs1932433 | s093s094 | 0.773 | 0.265 | 0.786 |
| PTPRC | rs1011338rs1932433rs6683595 | s093s094s095 | 0.695 | 0.222 | 0.773 |
| PTPRC | rs1011338rs1932433rs6683595rs11589894 | s093s094s095s096 | 1.000 | 0.279 | 0.793 |
| PTPRC | rs1932433rs6683595 | s094s095 | 0.780 | 0.223 | 0.762 |
| PTPRC | rs1932433rs6683595rs11589894 | s094s095s096 | 0.780 | 0.272 | 0.766 |
| PTPRC | rs1932433rs6683595rs11589894rs1932435 | s094s095s096s097 | 0.895 | 0.366 | 0.870 |
| PTPRC | rs6683595rs11589894 | s095s096 | 0.637 | 0.231 | 0.846 |
| PTPRC | rs6683595rs11589894rs1932435 | s095s096s097 | 0.799 | 0.326 | 0.582 |
| PTPRC | rs6683595rs11589894rs1932435rs874909 | s095s096s097s098 | 0.827 | 0.472 | 0.939 |
| PTPRC | rs11589894rs1932435 | s096s097 | 0.871 | 0.317 | 0.744 |
| PTPRC | rs11589894rs1932435rs874909 | s096s097s098 | 0.961 | 0.472 | 0.906 |
| PTPRC | rs1932435rs874909 | s097s098 | 0.880 | 0.273 | 0.726 |
| RAG1 | rs872053rs3740955 | s457s458 | 0.386 | 0.127 | 0.752 |
| RAG1 | rs872053rs3740955rs1056403 | s457s458s459 | 0.385 | 0.328 | 0.761 |
| RAG1 | rs3740955rs1056403 | s458s459 | 0.507 | 0.237 | 0.721 |
| RAG2 | rs7104753rs867801 | s460s461 | 0.689 | 0.104 | 0.129 |
| RAG2 | rs7104753rs867801rs867804 | s460s461s462 | 0.689 | 0.104 | 0.129 |
| RAG2 | rs867801rs867804 | s461s462 | 0.445 | 0.913 | 0.593 |
| RANGAP1 | rs1953rs71948 | s716s717 | 0.472 | 0.121 | 0.924 |
| RANGAP1 | rs1953rs71948rs104974 | s716s717s718 | 0.510 | 0.152 | 1.000 |
| RANGAP1 | rs1953rs71948rs104974rs139513 | s716s717s718s719 | 0.553 | 0.197 | 0.971 |
| RANGAP1 | rs71948rs104974 | s717s718 | 0.367 | 0.028 | 0.768 |
| RANGAP1 | rs71948rs104974rs139513 | s717s718s719 | 0.438 | 0.058 | 0.787 |
| RANGAP1 | rs71948rs104974rs139513rs5751072 | s717s718s719s720 | 0.580 | 0.284 | 0.909 |
| RANGAP1 | rs104974rs139513 | s718s719 | 0.271 | 0.042 | 0.642 |
| RANGAP1 | rs104974rs139513rs5751072 | s718s719s720 | 0.395 | 0.152 | 0.854 |
| RANGAP1 | rs104974rs139513rs5751072rs2235852 | s718s719s720s721 | 0.395 | 0.152 | 0.837 |
| RANGAP1 | rs139513rs5751072 | s719s720 | 0.141 | 0.059 | 0.540 |
| RANGAP1 | rs139513rs5751072rs2235852 | s719s720s721 | 0.125 | 0.059 | 0.522 |
| RANGAP1 | rs5751072rs2235852 | s720s721 | 0.838 | 0.134 | 0.248 |
| RAPH1 | rs2247094rs11679740 | s192s193 | 0.103 | 0.458 | 0.387 |
| RAPH1 | rs2247094rs11679740rs2469950 | s192s193s194 | 0.078 | 0.534 | 0.513 |
| RAPH1 | rs2247094rs11679740rs2469950rs2305417 | s192s193s194s195 | 0.185 | 0.562 | 0.440 |
| RAPH1 | rs11679740rs2469950 | s193s194 | 0.036 | 0.434 | 0.069 |
| RAPH1 | rs11679740rs2469950rs2305417 | s193s194s195 | 0.112 | 0.447 | 0.146 |
| RAPH1 | rs11679740rs2469950rs2305417rs7423567 | s193s194s195s196 | 1.000 | 0.237 | 0.600 |
| RAPH1 | rs2469950rs2305417 | s194s195 | 0.051 | 0.620 | 0.283 |
| RAPH1 | rs2469950rs2305417rs7423567 | s194s195s196 | 0.436 | 1.3E-10 | 0.298 |
| RAPH1 | rs2305417rs7423567 | s195s196 | 0.646 | 1.000 | 1.000 |
| RASSF5 | rs4845108rs7523985 | s099s100 | 0.981 | 0.096 | 0.736 |
| RASSF5 | rs4845108rs7523985rs7530746 | s099s100s101 | 0.489 | 0.492 | 0.783 |
| RASSF5 | rs4845108rs7523985rs7530746rs6685780 | s099s100s101s102 | 0.534 | 0.438 | 0.282 |
| RASSF5 | rs7523985rs7530746 | s100s101 | 0.775 | 0.340 | 0.594 |
| RASSF5 | rs7523985rs7530746rs6685780 | s100s101s102 | 0.522 | 0.503 | 0.212 |
| RASSF5 | rs7523985rs7530746rs6685780rs11119006 | s100s101s102s103 | 0.570 | 0.905 | 0.314 |
| RASSF5 | rs7530746rs6685780 | s101s102 | 0.189 | 0.441 | 0.365 |
| RASSF5 | rs7530746rs6685780rs11119006 | s101s102s103 | 0.014 | 0.362 | 0.094 |
| RASSF5 | rs7530746rs6685780rs11119006rs11119018 | s101s102s103s104 | 0.184 | 0.251 | 0.063 |
| RASSF5 | rs6685780rs11119006 | s102s103 | 0.147 | 0.450 | 0.392 |
| RASSF5 | rs6685780rs11119006rs11119018 | s102s103s104 | 0.372 | 0.189 | 0.407 |
| RASSF5 | rs6685780rs11119006rs11119018rs12569261 | s102s103s104s105 | 0.841 | 0.307 | 0.678 |
| RASSF5 | rs11119006rs11119018 | s103s104 | 0.626 | 0.378 | 0.961 |
| RASSF5 | rs11119006rs11119018rs12569261 | s103s104s105 | 0.901 | 0.528 | 0.943 |
| RASSF5 | rs11119006rs11119018rs12569261rs10442694 | s103s104s105s106 | 0.726 | 1.000 | 0.947 |
| RASSF5 | rs11119018rs12569261 | s104s105 | 0.969 | 0.794 | 0.824 |
| RASSF5 | rs11119018rs12569261rs10442694 | s104s105s106 | 0.635 | 0.913 | 0.902 |
| RASSF5 | rs11119018rs12569261rs10442694rs4845109 | s104s105s106s107 | 0.371 | 0.348 | 0.773 |
| RASSF5 | rs12569261rs10442694 | s105s106 | 0.604 | 0.611 | 0.999 |
| RASSF5 | rs12569261rs10442694rs4845109 | s105s106s107 | 0.521 | 0.667 | 0.820 |
| RASSF5 | rs12569261rs10442694rs4845109rs7555149 | s105s106s107s108 | 0.648 | 0.279 | 0.801 |
| RASSF5 | rs10442694rs4845109 | s106s107 | 0.415 | 0.728 | 0.633 |
| RASSF5 | rs10442694rs4845109rs7555149 | s106s107s108 | 0.591 | 0.599 | 0.783 |
| RASSF5 | rs10442694rs4845109rs7555149rs7527917 | s106s107s108s109 | 0.625 | 0.611 | 0.970 |
| RASSF5 | rs4845109rs7555149 | s107s108 | 0.421 | 1.000 | 0.644 |
| RASSF5 | rs4845109rs7555149rs7527917 | s107s108s109 | 0.494 | 0.659 | 0.980 |
| RASSF5 | rs4845109rs7555149rs7527917rs6660116 | s107s108s109s110 | 0.632 | 1.000 | 0.925 |
| RASSF5 | rs7555149rs7527917 | s108s109 | 0.898 | 0.879 | 0.976 |
| RASSF5 | rs7555149rs7527917rs6660116 | s108s109s110 | 0.975 | 0.440 | 0.810 |
| RASSF5 | rs7555149rs7527917rs6660116rs11589 | s108s109s110s111 | 0.719 | 0.310 | 0.830 |
| RASSF5 | rs7527917rs6660116 | s109s110 | 0.937 | 0.308 | 0.653 |
| RASSF5 | rs7527917rs6660116rs11589 | s109s110s111 | 0.461 | 0.281 | 0.648 |
| RASSF5 | rs6660116rs11589 | s110s111 | 0.564 | 0.506 | 0.784 |
| RFX5 | rs7552906rs1752387 | s069s070 | 0.236 | 0.318 | 0.085 |
| RFXAP | rs3814802rs1980881 | s537s538 | 0.290 | 0.238 | 0.992 |
| RFXAP | rs3814802rs1980881rs9547679 | s537s538s539 | 0.555 | 0.081 | 0.794 |
| RFXAP | rs1980881rs9547679 | s538s539 | 0.441 | 0.262 | 0.520 |
| SPI1 | rs10838698rs10769258 | s463s464 | 0.424 | 0.170 | 0.589 |
| SPI1 | rs10838698rs10769258rs896817 | s463s464s465 | 0.618 | 0.003 | 0.589 |
| SPI1 | rs10838698rs10769258rs896817rs7940536 | s463s464s465s466 | 0.618 | 0.003 | 0.589 |
| SPI1 | rs10769258rs896817 | s464s465 | 0.787 | 0.125 | 0.716 |
| SPI1 | rs10769258rs896817rs7940536 | s464s465s466 | 0.787 | 0.125 | 0.716 |
| SPI1 | rs896817rs7940536 | s465s466 | 0.787 | 0.127 | 0.715 |
| STAT1 | rs1400657rs3771300 | s176s177 | 0.218 | 0.365 | 0.170 |
| STAT1 | rs1400657rs3771300rs13395505 | s176s177s178 | 0.290 | 0.242 | 0.092 |
| STAT1 | rs1400657rs3771300rs13395505rs1914408 | s176s177s178s179 | 0.287 | 6.9E-10 | 0.175 |
| STAT1 | rs3771300rs13395505 | s177s178 | 0.277 | 0.297 | 0.086 |
| STAT1 | rs3771300rs13395505rs1914408 | s177s178s179 | 0.265 | 0.425 | 0.167 |
| STAT1 | rs3771300rs13395505rs1914408rs2280234 | s177s178s179s180 | 0.565 | 0.441 | 0.298 |
| STAT1 | rs13395505rs1914408 | s178s179 | 0.380 | 0.286 | 0.210 |
| STAT1 | rs13395505rs1914408rs2280234 | s178s179s180 | 0.482 | 0.556 | 0.384 |
| STAT1 | rs13395505rs1914408rs2280234rs7562024 | s178s179s180s181 | 0.491 | 0.336 | 0.869 |
| STAT1 | rs1914408rs2280234 | s179s180 | 0.569 | 0.431 | 0.551 |
| STAT1 | rs1914408rs2280234rs7562024 | s179s180s181 | 0.673 | 0.729 | 0.793 |
| STAT1 | rs1914408rs2280234rs7562024rs12693591 | s179s180s181s182 | 0.716 | 0.768 | 0.957 |
| STAT1 | rs2280234rs7562024 | s180s181 | 0.753 | 0.446 | 0.824 |
| STAT1 | rs2280234rs7562024rs12693591 | s180s181s182 | 0.754 | 0.746 | 0.949 |
| STAT1 | rs2280234rs7562024rs12693591rs10173099 | s180s181s182s183 | 0.525 | 0.884 | 0.980 |
| STAT1 | rs7562024rs12693591 | s181s182 | 0.690 | 0.513 | 0.826 |
| STAT1 | rs7562024rs12693591rs10173099 | s181s182s183 | 0.831 | 0.959 | 0.949 |
| STAT1 | rs7562024rs12693591rs10173099rs13029532 | s181s182s183s184 | 0.696 | 0.267 | 0.893 |
| STAT1 | rs12693591rs10173099 | s182s183 | 0.840 | 0.957 | 0.784 |
| STAT1 | rs12693591rs10173099rs13029532 | s182s183s184 | 0.959 | 0.116 | 0.635 |
| STAT1 | rs10173099rs13029532 | s183s184 | 0.995 | 0.348 | 0.467 |
| STAT3 | rs1053023rs1053005 | s611s612 | 0.850 | 0.745 | 0.682 |
| STAT3 | rs1053023rs1053005rs3809758 | s611s612s613 | 0.765 | 0.517 | 0.599 |
| STAT3 | rs1053023rs1053005rs3809758rs9912773 | s611s612s613s614 | 0.133 | 1.2E-04 | 0.050 |
| STAT3 | rs1053005rs3809758 | s612s613 | 0.800 | 0.532 | 0.513 |
| STAT3 | rs1053005rs3809758rs9912773 | s612s613s614 | 0.133 | 0.091 | 0.054 |
| STAT3 | rs1053005rs3809758rs9912773rs744166 | s612s613s614s615 | 0.218 | 0.032 | 0.051 |
| STAT3 | rs3809758rs9912773 | s613s614 | 0.471 | 0.067 | 0.057 |
| STAT3 | rs3809758rs9912773rs744166 | s613s614s615 | 0.343 | 0.172 | 0.101 |
| STAT3 | rs3809758rs9912773rs744166rs1026916 | s613s614s615s616 | 0.317 | 0.083 | 0.085 |
| STAT3 | rs9912773rs744166 | s614s615 | 0.817 | 0.689 | 0.972 |
| STAT3 | rs9912773rs744166rs1026916 | s614s615s616 | 0.802 | 0.841 | 0.342 |
| STAT3 | rs9912773rs744166rs1026916rs7211777 | s614s615s616s617 | 0.951 | 0.274 | 0.860 |
| STAT3 | rs744166rs1026916 | s615s616 | 0.544 | 0.564 | 0.962 |
| STAT3 | rs744166rs1026916rs7211777 | s615s616s617 | 0.843 | 0.098 | 0.555 |
| STAT3 | rs1026916rs7211777 | s616s617 | 0.832 | 0.119 | 0.512 |
| STAT4 | rs3024896rs3024861 | s185s186 | 0.492 | 5.4E-07 | 0.526 |
| STAT4 | rs3024896rs3024861rs7601754 | s185s186s187 | 0.049 | 0.223 | 0.022 |
| STAT4 | rs3024896rs3024861rs7601754rs6434435 | s185s186s187s188 | 0.155 | 0.264 | 0.235 |
| STAT4 | rs3024861rs7601754 | s186s187 | 0.063 | 0.861 | 0.887 |
| STAT4 | rs3024861rs7601754rs6434435 | s186s187s188 | 0.615 | 0.276 | 0.615 |
| STAT4 | rs3024861rs7601754rs6434435rs6752770 | s186s187s188s189 | 0.200 | 0.251 | 0.076 |
| STAT4 | rs7601754rs6434435 | s187s188 | 0.138 | 0.248 | 0.165 |
| STAT4 | rs7601754rs6434435rs6752770 | s187s188s189 | 0.150 | 0.248 | 0.061 |
| STAT4 | rs7601754rs6434435rs6752770rs1031508 | s187s188s189s190 | 0.496 | 0.505 | 0.107 |
| STAT4 | rs6434435rs6752770 | s188s189 | 0.211 | 0.265 | 0.113 |
| STAT4 | rs6434435rs6752770rs1031508 | s188s189s190 | 0.710 | 0.539 | 0.346 |
| STAT4 | rs6434435rs6752770rs1031508rs7572482 | s188s189s190s191 | 0.637 | 0.606 | 0.158 |
| STAT4 | rs6752770rs1031508 | s189s190 | 0.493 | 0.904 | 0.487 |
| STAT4 | rs6752770rs1031508rs7572482 | s189s190s191 | 0.091 | 0.873 | 0.085 |
| STAT4 | rs1031508rs7572482 | s190s191 | 0.258 | 0.795 | 0.708 |
| TAP1 | rs2071541rs735883 | s289s290 | 0.184 | 0.141 | 0.064 |
| TAP1 | rs2071541rs735883rs4148880 | s289s290s291 | 0.132 | 0.155 | 0.038 |
| TAP1 | rs2071541rs735883rs4148880rs2284190 | s289s290s291s292 | 0.231 | 0.105 | 0.168 |
| TAP1 | rs735883rs4148880 | s290s291 | 0.348 | 0.071 | 0.033 |
| TAP1 | rs735883rs4148880rs2284190 | s290s291s292 | 0.495 | 0.068 | 0.088 |
| TAP1 | rs4148880rs2284190 | s291s292 | 0.357 | 0.028 | 0.040 |
| TAP2 | rs13501rs241448 | s284s285 | 0.459 | 1.000 | 0.806 |
| TAP2 | rs13501rs241448rs241447 | s284s285s286 | 0.491 | 1.000 | 0.806 |
| TAP2 | rs13501rs241448rs241447rs241424 | s284s285s286s287 | 0.170 | 0.679 | 0.276 |
| TAP2 | rs241448rs241447 | s285s286 | 0.293 | 0.543 | 0.284 |
| TAP2 | rs241448rs241447rs241424 | s285s286s287 | 0.464 | 0.195 | 0.086 |
| TAP2 | rs241448rs241447rs241424rs4713598 | s285s286s287s288 | 0.585 | 0.013 | 0.001 |
| TAP2 | rs241447rs241424 | s286s287 | 0.464 | 0.195 | 0.086 |
| TAP2 | rs241447rs241424rs4713598 | s286s287s288 | 0.585 | 0.013 | 0.002 |
| TAP2 | rs241424rs4713598 | s287s288 | 0.642 | 0.105 | 0.088 |
| TAPBP | rs1059288rs2071888 | s295s296 | 0.086 | 0.570 | 0.395 |
| TAPBP | rs1059288rs2071888rs3106190 | s295s296s297 | 0.219 | 0.082 | 0.184 |
| TAPBP | rs2071888rs3106190 | s296s297 | 0.219 | 0.083 | 0.182 |
| TBX21 | rs16946264rs11079788 | s618s619 | 0.186 | 0.012 | 0.130 |
| TGFB1 | rs747857rs2278422 | s659s660 | 0.529 | 0.156 | 0.770 |
| TGFB1 | rs747857rs2278422rs4803455 | s659s660s662 | 0.677 | 0.395 | 0.916 |
| TGFB1 | rs747857rs2278422rs4803455rs2241715 | s659s660s662s663 | 0.094 | 0.245 | 0.201 |
| TGFB1 | rs2278422rs4803455 | s660s662 | 0.867 | 0.847 | 1.000 |
| TGFB1 | rs2278422rs4803455rs2241715 | s660s662s663 | 0.717 | 0.716 | 0.523 |
| TGFB1 | rs4803455rs2241715 | s662s663 | 0.286 | 0.020 | 0.155 |
| TIRAP | rs563011rs1893352 | s490s491 | 0.206 | 1.000 | 0.334 |
| TIRAP | rs563011rs1893352rs1786704 | s490s491s492 | 0.443 | 0.024 | 0.580 |
| TIRAP | rs1893352rs1786704 | s491s492 | 0.928 | 0.308 | 0.432 |
| TLR3 | rs7657186rs5743312 | s263s264 | 0.404 | 0.569 | 0.227 |
| TLR3 | rs7657186rs5743312rs3775292 | s263s264s265 | 0.463 | 0.756 | 0.397 |
| TLR3 | rs7657186rs5743312rs3775292rs3775291 | s263s264s265s266 | 0.076 | 0.256 | 0.292 |
| TLR3 | rs5743312rs3775292 | s264s265 | 0.946 | 0.524 | 0.822 |
| TLR3 | rs5743312rs3775292rs3775291 | s264s265s266 | 0.963 | 0.550 | 0.166 |
| TLR3 | rs3775292rs3775291 | s265s266 | 0.805 | 0.279 | 0.542 |
| TLR4 | rs1927911rs2149356 | s405s406 | 0.189 | 0.448 | 0.095 |
| TMEM37 | rs7602788rs2587708 | s172s173 | 0.301 | 0.035 | 0.170 |
| TMEM37 | rs7602788rs2587708rs2579632 | s172s173s174 | 0.228 | 0.639 | 0.050 |
| TMEM37 | rs7602788rs2587708rs2579632rs2278565 | s172s173s174s175 | 0.271 | 0.337 | 0.367 |
| TMEM37 | rs2587708rs2579632 | s173s174 | 0.696 | 0.062 | 0.102 |
| TMEM37 | rs2587708rs2579632rs2278565 | s173s174s175 | 0.876 | 0.197 | 0.463 |
| TMEM37 | rs2579632rs2278565 | s174s175 | 0.740 | 0.003 | 0.133 |
| TNFRSF17 | rs2017662rs12597429 | s580s581 | 0.624 | 0.680 | 0.930 |
| TNFRSF1B | rs496888rs976881 | s014s015 | 0.286 | 0.177 | 0.199 |
| TNFRSF1B | rs496888rs976881rs5746026 | s014s015s016 | 0.285 | 0.199 | 0.198 |
| TNFRSF1B | rs496888rs976881rs5746026rs5746051 | s014s015s016s017 | 0.307 | 0.559 | 0.289 |
| TNFRSF1B | rs976881rs5746026 | s015s016 | 0.266 | 0.227 | 0.095 |
| TNFRSF1B | rs976881rs5746026rs5746051 | s015s016s017 | 0.524 | 0.027 | 0.141 |
| TNFRSF1B | rs976881rs5746026rs5746051rs1061624 | s015s016s017s018 | 0.624 | 0.111 | 0.344 |
| TNFRSF1B | rs5746026rs5746051 | s016s017 | 0.666 | 0.081 | 0.370 |
| TNFRSF1B | rs5746026rs5746051rs1061624 | s016s017s018 | 0.894 | 0.145 | 0.633 |
| TNFRSF1B | rs5746051rs1061624 | s017s018 | 0.894 | 0.151 | 0.639 |
| TNFRSF8 | rs1318008rs4491070 | s001s002 | 0.586 | 0.321 | 0.484 |
| TNFRSF8 | rs1318008rs4491070rs6690493 | s001s002s003 | 0.835 | 0.248 | 0.616 |
| TNFRSF8 | rs1318008rs4491070rs6690493rs2297875 | s001s002s003s004 | 0.103 | 0.034 | 0.126 |
| TNFRSF8 | rs4491070rs6690493 | s002s003 | 0.952 | 0.290 | 0.605 |
| TNFRSF8 | rs4491070rs6690493rs2297875 | s002s003s004 | 0.696 | 0.219 | 0.446 |
| TNFRSF8 | rs4491070rs6690493rs2297875rs12133231 | s002s003s004s005 | 0.016 | 0.575 | 0.060 |
| TNFRSF8 | rs6690493rs2297875 | s003s004 | 0.631 | 0.145 | 0.432 |
| TNFRSF8 | rs6690493rs2297875rs12133231 | s003s004s005 | 0.096 | 0.398 | 0.225 |
| TNFRSF8 | rs6690493rs2297875rs12133231rs1201110 | s003s004s005s006 | 0.044 | 0.920 | 0.677 |
| TNFRSF8 | rs2297875rs12133231 | s004s005 | 0.081 | 0.844 | 0.198 |
| TNFRSF8 | rs2297875rs12133231rs1201110 | s004s005s006 | 0.058 | 0.849 | 0.028 |
| TNFRSF8 | rs2297875rs12133231rs1201110rs562844 | s004s005s006s007 | 0.008 | 0.962 | 0.077 |
| TNFRSF8 | rs12133231rs1201110 | s005s006 | 0.404 | 0.394 | 0.022 |
| TNFRSF8 | rs12133231rs1201110rs562844 | s005s006s007 | 3.9E-04 | 0.557 | 0.057 |
| TNFRSF8 | rs12133231rs1201110rs562844rs501525 | s005s006s007s008 | 0.166 | 0.095 | 0.324 |
| TNFRSF8 | rs1201110rs562844 | s006s007 | 1.9E-04 | 0.199 | 8.4E-04 |
| TNFRSF8 | rs1201110rs562844rs501525 | s006s007s008 | 0.017 | 0.350 | 0.010 |
| TNFRSF8 | rs1201110rs562844rs501525rs1148476 | s006s007s008s009 | 0.180 | 1.000 | 0.069 |
| TNFRSF8 | rs562844rs501525 | s007s008 | 0.075 | 0.124 | 0.622 |
| TNFRSF8 | rs562844rs501525rs1148476 | s007s008s009 | 0.544 | 0.681 | 0.830 |
| TNFRSF8 | rs562844rs501525rs1148476rs535068 | s007s008s009s010 | 0.297 | 0.706 | 0.843 |
| TNFRSF8 | rs501525rs1148476 | s008s009 | 0.941 | 0.672 | 0.892 |
| TNFRSF8 | rs501525rs1148476rs535068 | s008s009s010 | 0.863 | 0.027 | 0.390 |
| TNFRSF8 | rs501525rs1148476rs535068rs3766735 | s008s009s010s011 | 0.808 | 0.122 | 0.751 |
| TNFRSF8 | rs1148476rs535068 | s009s010 | 0.825 | 0.195 | 0.327 |
| TNFRSF8 | rs1148476rs535068rs3766735 | s009s010s011 | 0.699 | 0.167 | 0.478 |
| TNFRSF8 | rs1148476rs535068rs3766735rs671106 | s009s010s011s012 | 0.840 | 0.190 | 0.525 |
| TNFRSF8 | rs535068rs3766735 | s010s011 | 0.578 | 0.190 | 0.687 |
| TNFRSF8 | rs535068rs3766735rs671106 | s010s011s012 | 0.783 | 6.9E-04 | 0.758 |
| TNFRSF8 | rs535068rs3766735rs671106rs755398 | s010s011s012s013 | 0.831 | 0.054 | 0.104 |
| TNFRSF8 | rs3766735rs671106 | s011s012 | 0.476 | 0.350 | 0.576 |
| TNFRSF8 | rs3766735rs671106rs755398 | s011s012s013 | 0.682 | 0.297 | 0.884 |
| TNFRSF8 | rs671106rs755398 | s012s013 | 0.853 | 0.985 | 0.847 |
| TNFSF13B | rs8181791rs1224141 | s540s541 | 0.377 | 0.104 | 0.685 |
| TNFSF13B | rs8181791rs1224141rs10508198 | s540s541s542 | 0.048 | 0.076 | 0.030 |
| TNFSF13B | rs8181791rs1224141rs10508198rs9520835 | s540s541s542s543 | 0.339 | 1.000 | 0.759 |
| TNFSF13B | rs1224141rs10508198 | s541s542 | 0.094 | 0.208 | 0.132 |
| TNFSF13B | rs1224141rs10508198rs9520835 | s541s542s543 | 0.606 | 1.000 | 0.593 |
| TNFSF13B | rs1224141rs10508198rs9520835rs1224147 | s541s542s543s544 | 0.585 | 1.000 | 0.400 |
| TNFSF13B | rs10508198rs9520835 | s542s543 | 0.493 | 0.924 | 0.850 |
| TNFSF13B | rs10508198rs9520835rs1224147 | s542s543s544 | 0.652 | 0.526 | 0.368 |
| TNFSF13B | rs10508198rs9520835rs1224147rs1224151 | s542s543s544s545 | 0.586 | 0.383 | 1.000 |
| TNFSF13B | rs9520835rs1224147 | s543s544 | 0.556 | 0.522 | 0.411 |
| TNFSF13B | rs9520835rs1224147rs1224151 | s543s544s545 | 0.285 | 0.354 | 0.010 |
| TNFSF13B | rs1224147rs1224151 | s544s545 | 0.481 | 1.000 | 0.201 |
| TNFSF4 | rs3861950rs7518045 | s075s076 | 0.681 | 0.469 | 0.820 |
| TNFSF4 | rs3861950rs7518045rs1234313 | s075s076s077 | 0.925 | 1.000 | 0.964 |
| TNFSF4 | rs3861950rs7518045rs1234313rs13343108 | s075s076s077s078 | 0.943 | 0.840 | 0.987 |
| TNFSF4 | rs7518045rs1234313 | s076s077 | 0.563 | 0.528 | 0.861 |
| TNFSF4 | rs7518045rs1234313rs13343108 | s076s077s078 | 0.903 | 0.823 | 0.929 |
| TNFSF4 | rs7518045rs1234313rs13343108rs11811856 | s076s077s078s079 | 0.954 | 0.871 | 0.878 |
| TNFSF4 | rs1234313rs13343108 | s077s078 | 0.987 | 0.802 | 0.886 |
| TNFSF4 | rs1234313rs13343108rs11811856 | s077s078s079 | 0.925 | 0.869 | 0.854 |
| TNFSF4 | rs13343108rs11811856 | s078s079 | 0.917 | 0.851 | 0.839 |
| TNFSF7 | rs1862511rs344591 | s635s636 | 0.987 | 4.4E-04 | 0.325 |
| TNFSF8 | rs3181362rs3789882 | s401s402 | 0.747 | 0.273 | 0.298 |
| TNFSF8 | rs3181362rs3789882rs3181360 | s401s402s403 | 0.826 | 0.229 | 0.283 |
| TNFSF8 | rs3181362rs3789882rs3181360rs3181354 | s401s402s403s404 | 0.036 | 0.130 | 0.089 |
| TNFSF8 | rs3789882rs3181360 | s402s403 | 0.797 | 0.038 | 0.269 |
| TNFSF8 | rs3789882rs3181360rs3181354 | s402s403s404 | 0.034 | 0.480 | 0.087 |
| TNFSF8 | rs3181360rs3181354 | s403s404 | 0.029 | 0.304 | 0.071 |
| TOLLIP | rs3829223rs3793964 | s431s432 | 0.395 | 0.895 | 0.750 |
| TOLLIP | rs3829223rs3793964rs5743899 | s431s432s433 | 0.605 | 0.606 | 0.842 |
| TOLLIP | rs3829223rs3793964rs5743899rs5743867 | s431s432s433s434 | 0.503 | 0.591 | 1.000 |
| TOLLIP | rs3793964rs5743899 | s432s433 | 0.303 | 0.835 | 0.836 |
| TOLLIP | rs3793964rs5743899rs5743867 | s432s433s434 | 0.352 | 0.789 | 0.825 |
| TOLLIP | rs5743899rs5743867 | s433s434 | 0.397 | 0.577 | 0.985 |
| TRAF3 | rs4906263rs941726 | s556s557 | 0.680 | 0.017 | 0.252 |
| TRAF3 | rs4906263rs941726rs12432777 | s556s557s558 | 0.698 | 0.052 | 0.241 |
| TRAF3 | rs4906263rs941726rs12432777rs12436181 | s556s557s558s559 | 0.632 | 0.035 | 0.594 |
| TRAF3 | rs941726rs12432777 | s557s558 | 0.976 | 0.490 | 0.829 |
| TRAF3 | rs941726rs12432777rs12436181 | s557s558s559 | 0.972 | 0.499 | 0.849 |
| TRAF3 | rs941726rs12432777rs12436181rs11624345 | s557s558s559s560 | 0.998 | 0.794 | 0.929 |
| TRAF3 | rs12432777rs12436181 | s558s559 | 0.971 | 0.492 | 0.631 |
| TRAF3 | rs12432777rs12436181rs11624345 | s558s559s560 | 0.996 | 0.732 | 0.878 |
| TRAF3 | rs12432777rs12436181rs11624345rs8009520 | s558s559s560s561 | 0.996 | 0.732 | 0.878 |
| TRAF3 | rs12436181rs11624345 | s559s560 | 0.995 | 0.728 | 0.869 |
| TRAF3 | rs12436181rs11624345rs8009520 | s559s560s561 | 0.995 | 0.728 | 0.869 |
| TRAF3 | rs12436181rs11624345rs8009520rs7140494 | s559s560s561s562 | 0.967 | 0.794 | 0.864 |
| TRAF3 | rs11624345rs8009520 | s560s561 | 0.995 | 0.726 | 0.868 |
| TRAF3 | rs11624345rs8009520rs7140494 | s560s561s562 | 0.967 | 0.792 | 0.863 |
| TRAF3 | rs11624345rs8009520rs7140494rs7156191 | s560s561s562s563 | 0.977 | 0.903 | 0.934 |
| TRAF3 | rs8009520rs7140494 | s561s562 | 0.879 | 0.578 | 0.649 |
| TRAF3 | rs8009520rs7140494rs7156191 | s561s562s563 | 0.874 | 0.886 | 0.820 |
| TRAF3 | rs8009520rs7140494rs7156191rs2896460 | s561s562s563s564 | 0.874 | 0.886 | 0.822 |
| TRAF3 | rs7140494rs7156191 | s562s563 | 0.860 | 0.974 | 0.911 |
| TRAF3 | rs7140494rs7156191rs2896460 | s562s563s564 | 0.860 | 0.972 | 0.894 |
| TRAF3 | rs7140494rs7156191rs2896460rs12586742 | s562s563s564s565 | 0.860 | 0.972 | 0.926 |
| TRAF3 | rs7156191rs2896460 | s563s564 | 0.860 | 0.972 | 0.894 |
| TRAF3 | rs7156191rs2896460rs12586742 | s563s564s565 | 0.860 | 0.972 | 0.926 |
| TRAF3 | rs7156191rs2896460rs12586742rs1131877 | s563s564s565s566 | 0.860 | 0.972 | 0.926 |
| TRAF3 | rs2896460rs12586742 | s564s565 | 0.594 | 0.944 | 0.861 |
| TRAF3 | rs2896460rs12586742rs1131877 | s564s565s566 | 0.594 | 0.944 | 0.861 |
| TRAF3 | rs2896460rs12586742rs1131877rs7154305 | s564s565s566s567 | 0.594 | 0.944 | 0.861 |
| TRAF3 | rs12586742rs1131877 | s565s566 | 0.594 | 0.944 | 0.861 |
| TRAF3 | rs12586742rs1131877rs7154305 | s565s566s567 | 0.594 | 0.944 | 0.861 |
| TRAF3 | rs12586742rs1131877rs7154305rs9671376 | s565s566s567s568 | 0.594 | 0.944 | 0.861 |
| TRAF3 | rs1131877rs7154305 | s566s567 | 0.594 | 0.944 | 0.861 |
| TRAF3 | rs1131877rs7154305rs9671376 | s566s567s568 | 0.594 | 0.944 | 0.861 |
| TRAF3 | rs7154305rs9671376 | s567s568 | 0.719 | 0.944 | 0.861 |
| TRAF5 | rs12723208rs11582143 | s129s130 | 0.934 | 0.646 | 0.838 |
| TRAF5 | rs12723208rs11582143rs4951522 | s129s130s131 | 0.548 | 0.756 | 0.681 |
| TRAF5 | rs12723208rs11582143rs4951522rs11119729 | s129s130s131s132 | 0.884 | 0.764 | 0.585 |
| TRAF5 | rs11582143rs4951522 | s130s131 | 0.775 | 0.553 | 0.707 |
| TRAF5 | rs11582143rs4951522rs11119729 | s130s131s132 | 0.898 | 0.816 | 0.611 |
| TRAF5 | rs4951522rs11119729 | s131s132 | 0.261 | 1.000 | 0.681 |
| TRAF6 | rs2303439rs331457 | s453s454 | 0.456 | 0.849 | 0.522 |
| TRAF6 | rs2303439rs331457rs4755453 | s453s454s455 | 0.459 | 0.396 | 0.668 |
| TRAF6 | rs2303439rs331457rs4755453rs5030411 | s453s454s455s456 | 0.335 | 0.019 | 0.506 |
| TRAF6 | rs331457rs4755453 | s454s455 | 0.850 | 0.255 | 0.488 |
| TRAF6 | rs331457rs4755453rs5030411 | s454s455s456 | 0.710 | 0.035 | 0.368 |
| TRAF6 | rs4755453rs5030411 | s455s456 | 0.970 | 0.064 | 0.306 |
| WDR27 | rs7758376rs2997875 | s315s316 | 0.259 | 0.118 | 0.519 |
| WDR27 | rs7758376rs2997875rs9477984 | s315s316s317 | 0.372 | 0.191 | 0.631 |
| WDR27 | rs7758376rs2997875rs9477984rs9478031 | s315s316s317s318 | 0.455 | 0.177 | 0.698 |
| WDR27 | rs2997875rs9477984 | s316s317 | 0.376 | 0.537 | 0.741 |
| WDR27 | rs2997875rs9477984rs9478031 | s316s317s318 | 0.461 | 0.768 | 0.792 |
| WDR27 | rs2997875rs9477984rs9478031rs1989643 | s316s317s318s319 | 0.467 | 0.899 | 0.813 |
| WDR27 | rs9477984rs9478031 | s317s318 | 0.726 | 0.773 | 0.658 |
| WDR27 | rs9477984rs9478031rs1989643 | s317s318s319 | 0.521 | 0.438 | 0.208 |
| WDR27 | rs9477984rs9478031rs1989643rs2865097 | s317s318s319s320 | 0.684 | 1.000 | 0.443 |
| WDR27 | rs9478031rs1989643 | s318s319 | 1.000 | 1.000 | 1.000 |
| WDR27 | rs9478031rs1989643rs2865097 | s318s319s320 | 0.379 | 0.846 | 0.488 |
| WDR27 | rs9478031rs1989643rs2865097rs9383492 | s318s319s320s321 | 0.530 | 1.000 | 0.701 |
| WDR27 | rs1989643rs2865097 | s319s320 | 0.747 | 0.340 | 0.456 |
| WDR27 | rs1989643rs2865097rs9383492 | s319s320s321 | 0.642 | 0.428 | 0.767 |
| WDR27 | rs1989643rs2865097rs9383492rs1001844 | s319s320s321s322 | 0.641 | 1.000 | 1.000 |
| WDR27 | rs2865097rs9383492 | s320s321 | 0.897 | 0.504 | 0.627 |
| WDR27 | rs2865097rs9383492rs1001844 | s320s321s322 | 0.860 | 0.529 | 0.749 |
| WDR27 | rs2865097rs9383492rs1001844rs9383493 | s320s321s322s323 | 0.814 | 0.529 | 0.754 |
| WDR27 | rs9383492rs1001844 | s321s322 | 0.755 | 0.219 | 0.517 |
| WDR27 | rs9383492rs1001844rs9383493 | s321s322s323 | 0.821 | 0.529 | 0.752 |
| WDR27 | rs9383492rs1001844rs9383493rs926449 | s321s322s323s324 | 0.659 | 0.505 | 0.691 |
| WDR27 | rs1001844rs9383493 | s322s323 | 0.729 | 0.524 | 0.729 |
| WDR27 | rs1001844rs9383493rs926449 | s322s323s324 | 0.607 | 0.481 | 0.708 |
| WDR27 | rs1001844rs9383493rs926449rs9383498 | s322s323s324s325 | 0.682 | 0.393 | 0.885 |
| WDR27 | rs9383493rs926449 | s323s324 | 0.914 | 0.469 | 0.748 |
| WDR27 | rs9383493rs926449rs9383498 | s323s324s325 | 0.657 | 0.245 | 0.554 |
| WDR27 | rs9383493rs926449rs9383498rs6941625 | s323s324s325s326 | 0.645 | 0.225 | 0.474 |
| WDR27 | rs926449rs9383498 | s324s325 | 0.689 | 0.502 | 0.981 |
| WDR27 | rs926449rs9383498rs6941625 | s324s325s326 | 0.566 | 6.8E-07 | 0.895 |
| WDR27 | rs926449rs9383498rs6941625rs9295018 | s324s325s326s327 | 0.806 | 0.323 | 0.974 |
| WDR27 | rs9383498rs6941625 | s325s326 | 0.301 | 0.095 | 0.967 |
| WDR27 | rs9383498rs6941625rs9295018 | s325s326s327 | 0.510 | 0.179 | 0.994 |
| WDR27 | rs9383498rs6941625rs9295018rs2494673 | s325s326s327s328 | 0.059 | 0.108 | 0.291 |
| WDR27 | rs6941625rs9295018 | s326s327 | 0.713 | 0.474 | 0.888 |
| WDR27 | rs6941625rs9295018rs2494673 | s326s327s328 | 0.939 | 0.447 | 0.776 |
| WDR27 | rs6941625rs9295018rs2494673rs2050062 | s326s327s328s329 | 0.574 | 0.632 | 0.616 |
| WDR27 | rs9295018rs2494673 | s327s328 | 0.824 | 0.696 | 0.682 |
| WDR27 | rs9295018rs2494673rs2050062 | s327s328s329 | 0.757 | 0.932 | 0.866 |
| WDR27 | rs9295018rs2494673rs2050062rs2473441 | s327s328s329s330 | 0.757 | 0.929 | 0.891 |
| WDR27 | rs2494673rs2050062 | s328s329 | 0.552 | 0.929 | 0.873 |
| WDR27 | rs2494673rs2050062rs2473441 | s328s329s330 | 0.552 | 0.932 | 0.919 |
| WDR27 | rs2494673rs2050062rs2473441rs12111411 | s328s329s330s331 | 0.122 | 0.769 | 0.925 |
| WDR27 | rs2050062rs2473441 | s329s330 | 0.813 | 0.944 | 0.897 |
| WDR27 | rs2050062rs2473441rs12111411 | s329s330s331 | 0.226 | 0.558 | 0.831 |
| WDR27 | rs2050062rs2473441rs12111411rs2494686 | s329s330s331s332 | 0.065 | 0.395 | 0.622 |
| WDR27 | rs2473441rs12111411 | s330s331 | 0.226 | 0.558 | 0.831 |
| WDR27 | rs2473441rs12111411rs2494686 | s330s331s332 | 0.065 | 0.395 | 0.194 |
| WDR27 | rs2473441rs12111411rs2494686rs9396946 | s330s331s332s333 | 0.140 | 0.014 | 0.564 |
| WDR27 | rs12111411rs2494686 | s331s332 | 0.035 | 0.398 | 0.181 |
| WDR27 | rs12111411rs2494686rs9396946 | s331s332s333 | 0.088 | 0.444 | 0.171 |
| WDR27 | rs12111411rs2494686rs9396946rs7775504 | s331s332s333s334 | 0.340 | 0.532 | 0.868 |
| WDR27 | rs2494686rs9396946 | s332s333 | 0.992 | 0.015 | 0.416 |
| WDR27 | rs2494686rs9396946rs7775504 | s332s333s334 | 0.419 | 0.171 | 0.851 |
| WDR27 | rs2494686rs9396946rs7775504rs7762296 | s332s333s334s335 | 0.603 | 0.898 | 0.219 |
| WDR27 | rs9396946rs7775504 | s333s334 | 0.271 | 0.041 | 0.797 |
| WDR27 | rs9396946rs7775504rs7762296 | s333s334s335 | 0.419 | 0.256 | 2.5E-04 |
| WDR27 | rs9396946rs7775504rs7762296rs6938667 | s333s334s335s336 | 0.280 | 0.489 | 0.171 |
| WDR27 | rs7775504rs7762296 | s334s335 | 0.640 | 0.769 | 0.990 |
| WDR27 | rs7775504rs7762296rs6938667 | s334s335s336 | 0.701 | 0.879 | 0.761 |
| WDR27 | rs7775504rs7762296rs6938667rs9478086 | s334s335s336s337 | 0.864 | 0.321 | 0.767 |
| WDR27 | rs7762296rs6938667 | s335s336 | 0.714 | 0.908 | 0.809 |
| WDR27 | rs7762296rs6938667rs9478086 | s335s336s337 | 0.804 | 0.779 | 0.006 |
| WDR27 | rs7762296rs6938667rs9478086rs3800544 | s335s336s337s338 | 0.588 | 0.307 | 0.440 |
| WDR27 | rs6938667rs9478086 | s336s337 | 0.650 | 0.514 | 0.484 |
| WDR27 | rs6938667rs9478086rs3800544 | s336s337s338 | 0.870 | 0.110 | 0.144 |
| WDR27 | rs6938667rs9478086rs3800544rs3823464 | s336s337s338s339 | 0.209 | 0.877 | 0.030 |
| WDR27 | rs9478086rs3800544 | s337s338 | 0.708 | 0.018 | 0.840 |
| WDR27 | rs9478086rs3800544rs3823464 | s337s338s339 | 0.228 | 0.987 | 0.208 |
| WDR27 | rs9478086rs3800544rs3823464rs3800546 | s337s338s339s340 | 0.258 | 1.000 | 0.923 |
| WDR27 | rs3800544rs3823464 | s338s339 | 0.503 | 0.876 | 0.654 |
| WDR27 | rs3800544rs3823464rs3800546 | s338s339s340 | 0.286 | 0.520 | 0.982 |
| WDR27 | rs3800544rs3823464rs3800546rs3823465 | s338s339s340s341 | 0.161 | 0.691 | 0.974 |
| WDR27 | rs3823464rs3800546 | s339s340 | 0.167 | 0.029 | 0.829 |
| WDR27 | rs3823464rs3800546rs3823465 | s339s340s341 | 0.180 | 0.721 | 0.875 |
| WDR27 | rs3823464rs3800546rs3823465rs4236176 | s339s340s341s342 | 0.190 | 0.760 | 0.807 |
| WDR27 | rs3800546rs3823465 | s340s341 | 0.110 | 0.577 | 0.759 |
| WDR27 | rs3800546rs3823465rs4236176 | s340s341s342 | 0.077 | 0.559 | 0.707 |
| WDR27 | rs3800546rs3823465rs4236176rs4716377 | s340s341s342s343 | 0.077 | 0.641 | 0.713 |
| WDR27 | rs3823465rs4236176 | s341s342 | 0.129 | 0.671 | 0.761 |
| WDR27 | rs3823465rs4236176rs4716377 | s341s342s343 | 0.129 | 0.671 | 0.761 |
| WDR27 | rs3823465rs4236176rs4716377rs4716380 | s341s342s343s344 | 0.146 | 0.741 | 0.840 |
| WDR27 | rs4236176rs4716377 | s342s343 | 0.352 | 0.361 | 0.898 |
| WDR27 | rs4236176rs4716377rs4716380 | s342s343s344 | 0.173 | 0.584 | 0.826 |
| WDR27 | rs4716377rs4716380 | s343s344 | 0.173 | 0.568 | 0.852 |
| XBP1 | rs2097461rs2239815 | s714s715 | 0.731 | 0.841 | 0.664 |
| ZAP70 | rs7425883rs6736735 | s133s134 | 0.368 | 0.314 | 0.195 |
| ZAP70 | rs7425883rs6736735rs2289918 | s133s134s135 | 0.436 | 0.010 | 0.638 |
| ZAP70 | rs7425883rs6736735rs2289918rs6714710 | s133s134s135s136 | 0.014 | 0.923 | 0.039 |
| ZAP70 | rs6736735rs2289918 | s134s135 | 0.779 | 0.803 | 0.746 |
| ZAP70 | rs6736735rs2289918rs6714710 | s134s135s136 | 0.017 | 0.880 | 0.678 |
| ZAP70 | rs6736735rs2289918rs6714710rs3192177 | s134s135s136s137 | 0.025 | 0.070 | 0.487 |
| ZAP70 | rs2289918rs6714710 | s135s136 | 0.087 | 0.612 | 0.722 |
| ZAP70 | rs2289918rs6714710rs3192177 | s135s136s137 | 0.021 | 1.000 | 0.217 |
| ZAP70 | rs2289918rs6714710rs3192177rs2278699 | s135s136s137s138 | 0.202 | 0.677 | 0.964 |
| ZAP70 | rs6714710rs3192177 | s136s137 | 0.146 | 1.000 | 0.214 |
| ZAP70 | rs6714710rs3192177rs2278699 | s136s137s138 | 0.216 | 0.185 | 0.489 |
| ZAP70 | rs3192177rs2278699 | s137s138 | 0.410 | 0.225 | 0.776 |
